# The feasibility and acceptability of an app‐based intervention with brief behavioural support (APPROACH) to promote brisk walking in people diagnosed with breast, prostate and colorectal cancer in the UK

**DOI:** 10.1002/cam4.7124

**Published:** 2024-03-26

**Authors:** Phillippa Lally, Fiona Kennedy, Susan Smith, Rebecca J. Beeken, Caroline Buck, Chloe Thomas, Nicholas Counsell, Lynda Wyld, Charlene Martin, Sarah Williams, Anna Roberts, Diana M. Greenfield, Jacqui Gath, Henry W. W. Potts, Nicholas Latimer, Lee Smith, Abi Fisher

**Affiliations:** ^1^ Department of Psychological Sciences University of Surrey Guildford Surrey UK; ^2^ Leeds Institute of Health Sciences University of Leeds Leeds UK; ^3^ Department of Behavioural Science and Health University College London London UK; ^4^ School of Health and Related Research University of Sheffield Sheffield UK; ^5^ Cancer Research UK & Cancer Trials Centre, Cancer Institute University College London London UK; ^6^ Department of Oncology and Metabolism University of Sheffield Sheffield UK; ^7^ Sheffield Teaching Hospitals NHS FT Weston Park Hospital Sheffield UK; ^8^ Independent Cancer Patients' Voice (ICPV) London UK; ^9^ Institute of Health Informatics University College London London UK; ^10^ The Centre for Health, Performance, and Wellbeing Anglia Ruskin University Cambridge UK

**Keywords:** brisk walking, cancer survivors, habits, mobile apps, physical activity, pilot study

## Abstract

**Introduction:**

Increased moderate to vigorous physical activity (MVPA) can improve clinical and psychosocial outcomes for people living with and beyond cancer (LWBC). This study aimed to assess the feasibility and acceptability of trial procedures in a pilot randomised controlled trial (RCT) of a theory‐driven app‐based intervention with behavioural support focused on promoting brisk walking (a form of MVPA) in people LWBC (APPROACH).

**Methods:**

Participants diagnosed with breast, prostate or colorectal cancer were recruited from a single UK hospital site. Assessments at baseline and 3 months included online questionnaires, device‐measured brisk walking (activPAL accelerometer) and self‐reported weight and height. Participants were randomised to intervention or control (care as usual). The intervention comprised a non‐cancer‐specific app to promote brisk walking (National Health Service ‘Active 10’) augmented with print information about habit formation, a walking planner and two behavioural support telephone calls. Feasibility and acceptability of trial procedures were explored. Initial estimates for physical activity informed a power calculation for a phase III RCT. A preliminary health economics analysis was conducted.

**Results:**

Of those medically eligible, 369/577 (64%) were willing to answer further eligibility questions and 90/148 (61%) of those eligible were enrolled. Feasibility outcomes, including retention (97%), assessment completion rates (>86%) and app download rates in the intervention group (96%), suggest that the trial procedures are acceptable and that the intervention is feasible. The phase III RCT will require 472 participants to be randomised. As expected, the preliminary health economic analyses indicate a high level of uncertainty around the cost‐effectiveness of the intervention.

**Conclusions:**

This pilot study demonstrates that a large trial of the brisk walking intervention with behavioural support is both feasible and acceptable to people LWBC. The results support progression onto a confirmatory phase III trial to determine the efficacy and cost‐effectiveness of the intervention.

## 1 INTRODUCTION

Cancer is a leading cause of death worldwide and accounts for over 167,000 deaths in the United Kingdom every year.^1^ However, advances in the detection and treatment of cancer have led to increased survival rates in recent years, with a rising population of people living with the immediate and long‐term effects of a cancer diagnosis and its treatments.^2^ It is estimated that there are presently over 375,000 new cases of cancer in the United Kingdom each year, and this is predicted to rise to over 500,000 by 2040.^3^ Long‐term effects include fatigue, low mood, persistent emotional distress and anxiety states, trauma‐related responses, reductions in physical capabilities, being at increased risk for development of other cancers and other chronic conditions and experiencing lower quality of life.^4^, ^5^, ^6^, ^7^, ^8^, ^9^ Supportive interventions that can mitigate some of these effects are urgently required and need to be cost‐effective, easily accessible and scalable to large, diverse populations.

### 1.1 Physical activity and cancer

A large body of evidence demonstrates that physical activity can improve many outcomes for people living with and beyond a cancer diagnosis (LWBC).^10^, ^11^, ^12^, ^13^, ^14^ Exercise (one domain of physical activity) is safe and recommended for people who are still undergoing cancer treatment and improves multiple physical and psychosocial outcomes.^15^ While breast, prostate and colorectal cancer comprise three of the four most commonly diagnosed cancers in the United Kingdom, these cancer types also demonstrate the strongest evidence supporting a positive role of physical activity on health and psychosocial outcomes after a cancer diagnosis.^16^, ^17^ This includes several systematic reviews and meta‐analyses presenting evidence of an inverse association between physical activity and the risk of all‐cause and cancer‐specific mortality in these cancer populations.^12^, ^13^, ^18^, ^19^, ^20^ The importance of physical activity after diagnosis is highlighted by Schmid and Leitzmann's systematic review reporting that an increase in physical activity by *any* amount was associated with reduced total mortality risk in people diagnosed with breast or colorectal cancer.^21^ Furthermore, meta‐analyses of hundreds of interventional trials find that higher levels of physical activity in people LWBC are associated with reduced sleep disturbance and pain, and improved emotional well‐being and quality of life.^22^, ^23^, ^24^ Reflecting this evidence, as well as the more recent recognition of the benefits of jointly increasing physical activity while reducing time spent sedentary (i.e. sitting time), the World Cancer Research Fund recommends that adults LWBC should aim to engage in ≥150 min of at least moderate‐intensity physical activity per week if possible, or aim to ‘move more and sit less’.^25^, ^26^, ^27^ To support people LWBC in engaging with these recommendations, the Independent Cancer Taskforce recommend that every person diagnosed with cancer should receive physical activity guidance as part of their care.^28^

Despite these recommendations, people LWBC are rarely provided with physical activity advice from their care team.^29^, ^30^, ^31^, ^32^ Qualitative exploration with healthcare professionals (HCP) including general practitioners, oncology nurses and specialised physicians has identified barriers such as lack of time in appointments, lack of knowledge of resources to direct patients to and not self‐identifying as the right person to provide this advice to people LWBC.^33^, ^34^, ^35^ These findings highlight the need to develop low‐cost, widely accessible resources for people LWBC that are feasible to implement into the cancer care pathway with a low burden to the HCP.

### 1.2 Physical activity interventions in people LWBC

Digital interventions offer the possibility of remotely delivering large‐scale physical activity interventions to people LWBC.^36^ In a recent scoping review of 231 trials using digital health interventions for people LWBC, Lee et al. reported that web‐based digital health technology was the most commonly used type of digital intervention (50%) and this was followed by mobile apps (13%). The UK Office of Communications reported that over 90% of the population own a smartphone in 2022, with 68% of people aged over 65 reporting that they personally use them.^37^ ^, p. 203^ As smartphone apps can offer scalable behaviour change intervention to a wider population at a relatively low cost once developed, this presents a promising opportunity to target older age groups who are also at higher risk of a cancer diagnosis.^38^ Furthermore, Khoo et al. reported that personal contact complementary to a smartphone intervention may improve intervention efficacy, with Wallbank et al. suggesting that this contact may help address any lack of personalisation that is inherently associated with using technology‐based supports.^39^, ^40^

Our group conducted a meta‐analysis of 15 studies of digital interventions and identified that digital behaviour change interventions may successfully increase physical activity rates among people LWBC by up to 49 min per week.^41^ However, only two studies tested apps, most follow‐up periods were only 3 months and studies were generally of low quality, highlighting the need for investigation with larger randomised controlled trials (RCTs), using device‐based, rather than self‐reported physical activity and with longer follow‐up than 3 months. In a more recent review of 18 studies investigating digital physical activity interventions for people diagnosed with breast cancer, Kang and Moon^42^ reported that half of these used apps to deliver the intervention. Similar to our findings, their meta‐analysis of five studies revealed that digital physical activity interventions significantly improved physical activity duration with a medium effect size in people diagnosed with breast cancer. These results were also supported by qualitative findings.^42^

In their study of 627 Canadian adults diagnosed with cancer, Ester and colleagues reported widespread ownership of smartphones (88%) along with considerable use of physical activity/health‐related apps in this sample (32%).^43^ Additionally, over 80% of respondents rated physical activity/health apps as useful or very useful for supporting physical activity engagement, suggesting that incorporating such apps would be an effective strategy with this population. While there are many health and fitness apps available to download, few studies have investigated whether these are suitable for promoting physical activity among people LWBC.^44^, ^45^, ^46^ In preparation for the current study, along with the aforementioned meta‐analysis, we conducted qualitative user experience research in 32 people diagnosed with breast, prostate and colorectal cancer. Participants were given apps that promote physical activity that are designed for the general public rather than specifically for those LWBC and we sought to assess the acceptability of this approach. In line with previous research, participants reported that they found the idea of an app‐based intervention appealing for physical activity promotion and should focus on walking.^45^, ^47^ This preference for walking was also reported in two recent reviews of over 100 studies of physical activity participation across all cancer types and treatment stages.^48^, ^49^ Previous research conducted by our group and others suggests that people LWBC find that walking is the most achievable form of physical activity both during and after treatment.^45^, ^48^ While after treatment has been identified by people LWBC as the preferred time to start physical activity programmes,^48^ evidence suggests that limited awareness about the benefits of physical activity engagement during treatment may also play a role in these findings.^48^, ^50^ In their recommendations for cancer survivorship, the American Cancer Society reported that engaging in exercise during treatment is associated with a positive impact on quality of life in this population.^51^ Moreover, there is preliminary evidence to support that physical activity during cancer treatment may improve treatment response and tolerance.^51^, ^52^, ^53^ In a study of 279 women diagnosed with breast cancer, Phillips et al.^47^ reported that a technology‐supported exercise intervention was rated as somewhat/very helpful at all stages of the cancer care pathway, with high interest during (83%) and after treatment (90%–93%). Physical activity research with people LWBC has primarily been conducted in people diagnosed with early‐stage cancers. However, advancements in treatment have led to improved survival in patients with diagnosed metastatic disease^54^ and the available physical activity guidelines are applicable to all people LWBC across the continuum of care inclusive of those with metastatic disease, albeit with more supervision and support.^55^ However, due to the experience of higher burden of symptoms among this group, compliance and adherence to physical activity can be challenging, with high drop‐out rates reported in some studies.^56^, ^57^, ^58^ Despite this challenge, Wilk and colleagues noted the importance of including patients with metastatic disease in studies as evidence supports the beneficial role of physical activity in supporting improvements in health and psychosocial outcomes in this population.^59^ Collectively, this evidence highlights the importance of conducting research to explore the acceptability of implementing physical activity interventions at all stages of the cancer care continuum and recognises the need for designing interventions that can be applied in practical contexts and delivered as part of routine contact and care.

The importance of physical activity guidance coming from a trusted source is well documented within the literature.^48^, ^60^, ^61^ In our qualitative research study, participants expressed a preference for the intervention being recommended by direct members of their care team (ideally their cancer nurse), badged under a recognised organisation (such as the UK National Health Service [NHS]).^45^, ^47^ This preference was also demonstrated in a qualitative study of 14 patients with breast cancer, where participants indicated that their belief in the credibility of the app would increase if it was recommended or validated by their healthcare professional.^62^ We conducted qualitative interviews with 19 cancer nurses and found willingness to embed app‐based referral programmes into care so long as there was evidence of efficacy.^63^

### 1.3 Objectives

Informed by habit theory,^64^ we developed an intervention that implements a multitude of behavioural change techniques that have shown promise in promoting physical activity.^65^, ^66^, ^67^ This complex intervention includes a publicly available app with additional brief behavioural support to promote brisk walking (as a form of MVPA) after a cancer diagnosis [APPROACH].^68^ The Medical Research Council published seminal guidance on the development and evaluation of complex interventions, and continuously emphasised the importance of assessing the feasibility and acceptability of interventions with pilot studies before progressing to larger‐scale evaluations of interventions.^69^, ^70^ The feasibility study should assess the criteria that will be necessary for the evaluation design (e.g. trial procedures) as well as the intervention itself.^70^ The guidance also asserted the importance of including economic considerations surrounding intervention effectiveness and recommended including an assessment of the likelihood of cost‐effectiveness at the feasibility stage of intervention development.^70^ Preliminary economic modelling is important to determine if the anticipated benefits of the intervention justify the costs involved, including the costs of additional research and this is essential for guiding the decision to proceed with larger‐scale evaluations.^71^ In addition to preliminary economic modelling, this feasibility study will allow for planning of a larger‐scale trial and inform on any necessary refinements to the intervention to improve engagement.^70^ Following this guidance, this paper describes a pilot study assessing the feasibility and acceptability of the outcome measures and trial procedures to assist in the planning of a confirmatory phase III RCT. This larger trial will determine the efficacy and cost‐effectiveness of the intervention. This pilot study also aimed to inform the larger RCT by obtaining estimates for the parameters required in the sample size calculation for the intended future primary outcome (such as estimates of the variability in each arm and dropout rate), and by implementing a preliminary health economic analysis.

## 2 METHODS

### 2.1 Design

The full protocol for the current pilot has been previously published.^68^ This was a single‐centre, two‐arm pilot RCT comparing an app‐based brisk walking intervention with behavioural support against a control (usual care) arm in people diagnosed with localised or metastatic breast, prostate or colorectal cancer. After completion of baseline assessments, participants were randomised using minimisation (1:1 allocation), stratified by cancer type and disease status (local vs. metastatic disease), to either the control or intervention arm.

### 2.2 Participants

Participants were individuals living with localised or metastatic breast, prostate or colorectal cancer recruited from a single hospital site in Yorkshire (UK). All participants were smartphone owners, able to provide informed consent, willing to answer online questionnaires and had access to a computer and email address. Patients who met any of the following criteria were excluded: had localised disease and it had been more than 6 months since completion of radical treatment (i.e. surgery to remove cancer, radiotherapy, systemic therapy with curative intent), were unable to understand spoken/written English, had an Eastern Cooperative Oncology Group (ECOG) performance status ≥3, a diagnosed cognitive impairment (e.g. dementia), a cognitive and/or physical impairment that prevents participation in brisk walking, a clinician‐estimated life expectancy of <6 months, or were receiving end of life care, due to having surgery to remove cancer in the next 5 months, were <6 weeks after surgery to remove cancer, reported already achieving 150 min of at least moderate‐intensity physical activity weekly, reported previous/current use of the intervention app (Active 10), or reported current or recent (<6 months) participation in a health behaviour change study. Hormone therapy was not considered a radical treatment as it is not a treatment with curative intent. A timeframe of within 6 months was selected based on previous research reporting a preference for receiving information from their clinical care team.^48^, ^60^, ^61^ This timeframe aligns with the assumption that people would still be receiving support within the NHS at this stage, rather than having transitioned into long‐term survivorship.

### 2.3 Procedure

Medical records (lists of patients seen at multidisciplinary team meetings) were screened for potential participants against a set of initial eligibility criteria. This included having a diagnosis of breast, prostate or colorectal cancer, being more than 6 weeks post‐surgery, being less than 6 months after finishing treatment (localised disease), not due surgery in the next 5 months, being able to provide consent, understanding English, having no diagnosis of cognitive impairment, not having an ECOG ≥3 and having clinician‐estimated life expectancy of over 6 months and/or not receiving end of life care. Identified patients were then sent a brief information letter about the study and could indicate their interest via telephone or email.

Further eligibility was assessed by telephone where potential participants were asked if they were able to understand and complete the assessments in English, if they had any health conditions that would prevent them from walking, what treatment they had completed and plans for future treatment. Their ECOG status was confirmed (based on hospital records). Their physical activity levels were assessed using the screening question ‘As a rule, do you do at least half an hour of moderate or vigorous exercise (that makes you breathe faster and feel warmer) on five or more days of the week?’ (ineligible if yes).^72^, ^73^ They were asked if they had taken part in a health behaviour study in the past 6 months (ineligible if yes), whether they owned a smartphone (ineligible if no), had access to a computer (ineligible if no), and if they have ever used an app for tracking activity before (ineligible if they named Active 10). If eligible, participants were sent an email with a link to the online participant information sheet and consent form. This was hosted on the electronic data capture tool REDCap.^74^, ^75^

At baseline, participants were sent a weighing scale (Seca 803 if they weighed less than 150 kg and Seca 813 if they weighed over 150 kg) and tape measure (Seca 201) with instructions on how to complete assessments. Participants were also sent an activPAL accelerometer (PAL Technologies Ltd., Glasgow, UK) to wear for 7 days and a log sheet to track their waking/sleeping times. Two links were sent to participants. One was to complete the main online baseline questionnaire and the other was to input their measurements in the anthropometrics questionnaire, both of which were hosted on REDCap.^75^ If participants found this challenging, they could contact the research team to enter their data over the phone. Table 1 presents the schedule of assessments and the measures included in the online questionnaire.

**TABLE 1:** Schedule of study assessments.

Assessment	Baseline (T0)	12–16 weeks from T0 (T1)
Demographics	X	
Medical Information	X	
Physical activity (GLTEQ)	X	X
Anthropometrics (height, weight, waist circumference)	X	X
Health‐related quality of life (EQ‐5D‐5L)	X	X
Cancer‐specific quality of life (FACT‐G)	X	X
Fatigue (FACIT‐F)	X	X
Sleep Quality (PSQI)	X	X
Anxiety (GAD‐7)	X	X
Depression (PHQ‐9)	X	X
Physical activity self‐efficacy (PAAI)	X	X
Self‐efficacy to manage cancer (CS‐SES)	X	X
Habit strength for walking (‘Going for a walk’ and ‘Walking briskly’) (SRBAI)	X	X
Health and social care service usage (CSRI)	X	X
Question about usage of any physical activity app		X
Question about usage of Active 10 app		X
Intervention engagement (DBCI Engagement Scale)		X
Chronotype (MEQ)		X

Abbreviations: CSRI, Client Service Receipt Inventory^76^; CS‐SES, Cancer Survivors Self‐Efficacy Scale^77^; DBCI Engagement Scale, digital behaviour change intervention Engagement Scale^78^; EQ‐5D‐5L, Five‐level EuroQol‐5D^79^; FACIT‐F, Functional Assessment of Chronic Illness Therapy‐Fatigue^80^; FACT‐G, Functional Assessment of Cancer‐General^81^; GAD‐7, General Anxiety Disorder Assessment^82^; GLTEQ, Godin Leisure‐Time Exercise Questionnaire^83^; MEQ, Morning‐Eveningness Questionnaire^84^; PAAI, Physical Activity Appraisal Inventory^85^; PHQ‐9, Patient Health Questionnaire‐9^86^; PSQI, Pittsburgh Sleep Quality Index^87^; SRBAI, Self‐Report Behavioural Automaticity Index.^88^

Participants in the intervention group were mailed an intervention pack containing a leaflet, a walking planner and a letter from their clinical care team. The leaflet provided information on the benefits of physical activity after a cancer diagnosis with a focus on brisk walking. Information on forming walking habits was also provided in the leaflet, along with instructions to download the freely available NHS Active 10 app. The Active 10 app encourages users to do 10 min of brisk walking (known as one ‘Active 10’) and at the time of the pilot study, allowed users the flexibility to set their own goal of completing between one and three Active 10 s each day. This was to support users to reach 30 min of at least moderate‐intensity physical activity each day. The app tracks activity and distinguishes between total walking and brisk walking. Users could see how many minutes per day they spent in each walking type. Brisk walking was captured by Active 10 when participants walked at a cadence of approximately 100 steps per minute or more.^89^ The weekly walking planner was designed to allow participants to engage in action planning and monitor their walking. The letter from their care team endorsed physical activity participation and provided an appointment time for their first intervention behavioural support video/telephone call. The first intervention call involved the facilitator discussing the physical activity guidelines for people LWBC, talking through the benefits of physical activity, using the intervention materials, setting goals and forming habits. Intervention participants were subsequently invited to a second call approximately 4 weeks after the first call to check if they are using the Active 10 app and if they are increasing their brisk walking, as well as talking through their goals and recapping the information provided in the first call. A detailed description of the behavioural change techniques employed across the intervention components is described in the published protocol.^68^ Participants in the control group were informed of their group allocation by telephone and continued with their standard care without any additional support. Three months after their randomisation date (T1), all participants were asked to complete the assessments and online questionnaires again.

### 2.4 Measures

#### 2.4.1 Sociodemographic and medical information

Participants' cancer diagnosis (date and type) and stage, treatment, prior cancer diagnoses and other health conditions (osteoporosis; osteoarthritis/degenerative arthritis; rheumatoid arthritis; type 1 diabetes; type 2 diabetes; asthma; a mental health condition; Parkinson's disease; dementia; heart disease; high blood pressure; lung disease; back pain; irregular heart rhythm) were collected from hospital records. Participants also self‐reported any comorbid health conditions from the same predefined list of conditions. Data from both sources were combined and where a comorbid condition was identified in either the medical records or by self‐report, this was coded as having this health condition. Similarly, participants were asked to self‐report any prior cancer diagnoses to their most recent diagnosis of breast, prostate or colorectal cancer (date and type) and where a prior cancer diagnosis was identified in either the medical records or by self‐report, this was coded as having had a prior cancer diagnosis. The type (surgery; radiotherapy; chemotherapy; hormone therapy; biological therapy) and stage of treatment (due to start; undergoing; completed; not had/having) were collected from the medical records. This was recorded at the time the participant was sent the baseline assessment pack, although this was difficult for researchers to confirm from records due to the possibility of attending other hospital sites for treatment(s). Participants self‐reported their age (years), gender (male; female), employment status (employed full‐time; employed part‐time; full‐time education; unemployed; retired; unable or too ill to work), education level (7 levels ranging from ‘no formal qualifications’ to ‘Masters/PhD/PGCE or equivalent’), marital status (married/in a relationship; single/divorced/separated; widowed), living arrangements (alone; with partner only; with family; with friends; in a residential care/nursing home) and ethnicity (White; Asian/Asian British; Black/African/Caribbean/Black British; Mixed/Multiple ethnic groups; other ethnic group). Socioeconomic position was determined from participants' postcodes and the English Index of Multiple Deprivation (IMD).^90^

#### 2.4.2 Feasibility outcomes

The feasibility and acceptability outcomes (listed in Table 2) were used to investigate the potential for this study design to be used in a phase III trial and to further inform the final sample size calculation. We pre‐specified that a study enrolment rate < 30% or a 3‐month retention rate < 65% would require a reconsideration of trial procedures to make them more acceptable to participants.^68^

**TABLE 2:** Feasibility outcomes.

Feasibility outcomes	Detail of specific outcome
Interest	% of medically eligible interested/willing to answer eligibility questions
Enrolment	% fully eligible patients enrolled
Acceptability of randomisation	% of participants who withdraw post‐randomisation (within 1 week of being informed)
	% potential participants who state that randomisation is their reason for declining
Feasibility of administering intervention	% of intervention group who received a behavioural support call
	% of intervention group who self‐reported downloading the app
Acceptability of intervention	% of participants who reported that no aspect of the intervention was useful
	% of participants in the intervention group who report using the app for less than a month
	% of withdrawals from the intervention group compared to control group
	% of reasons for withdrawal relating to the intervention
Retention rate	% of participants, in each group, who complete any of the T1 follow‐up assessment
Acceptability of outcome assessments	% of participants who consent who complete any baseline assessments
	Completion rates, in each group, for each of the assessments at baseline and follow‐up
Willingness to consent to linkage with HES/NCRAS registries for long‐term follow‐up	% of participants who consent for this aspect of the study
Acceptability of online assessments	% of participants who required help to complete the questionnaires % of potential participants who give this method of data collection as a reason for declining to participate
Acceptability of providing informed consent online	% of participants who give online informed consent as a reason for declining
Proportion of screened participants ineligible and reasons for ineligibility	Number of participants screened and deemed ineligible for each inclusion/exclusion criteria
Potential sociodemographic biases in recruitment	Comparison of sample demographics with hospital level data on patients with breast, prostate and colorectal cancer
Fidelity of intervention delivery in telephone/video calls	Average % of required behaviour change techniques covered in intervention calls
Contamination of the control group	% of participants who report using the Active 10 app or that a health professional recommended it to them, during the study period

Abbreviations: HES, Hospital Episode Statistics; NCRAS, National Cancer Registration and Analysis Service.

#### 2.4.3 Intervention feasibility

During their first behavioural support call, the researcher recorded if participants in the intervention group had downloaded the Active 10 app (before the call, during the call) or had not downloaded it. Intervention participants were also asked how long they had used the app for (once; 1 week; 2 weeks; 1 month; 2 months; 3 months) in the follow‐up online questionnaire. Participants were asked to rate how useful they found the intervention using a Likert scale (not at all useful; slightly useful; somewhat useful; very useful; extremely useful).

##### Linking to UK cancer registries

The consent form included an optional additional consent to access Hospital Episode Statistics (HES) and National Cancer Registration and Analysis Service (NCRAS) data about participants. This was to assess *willingness* to give this consent, as we may wish to explore the impact of the intervention on longer‐term cancer outcomes in the RCT, but this data was not accessed in the pilot.

##### Potential sociodemographic biases

We intended to collect anonymous sociodemographic data on patients who were potentially eligible to participate but who did not participate. This was not possible due to data protection concerns. The hospital site was, however, able to provide aggregate anonymous data on cancer type, sex, ethnicity, age and IMD scores for all patients who were diagnosed with breast, prostate or colorectal cancer (due to how the data was stored this included those diagnosed with localised breast, prostate or colorectal cancer and those diagnosed with metastatic breast or colorectal cancer) between August 2021 and August 2022, regardless of participation, to allow identification of any recruitment bias.

##### Fidelity of intervention calls

The content of the intervention calls is outlined in our published protocol.^68^ Intervention calls were designed to include 25 behaviour change techniques (BCTs) from the Behaviour Change Technique Taxonomy v1.^67^ A 25‐item checklist was created by the researchers based on these BCTs. Each BCT was coded as either delivered or not delivered by examining the intervention call transcripts. One researcher (SW) carried out the coding of the intervention calls with a second researcher (SS) coding a subset of calls (*n* = 5). It was agreed that an 80% level of agreement would be acceptable. Any discrepancies that exceeded 20% were discussed among the researchers until consensus was reached. This occurred for 20% of the transcripts that were double‐coded (*n* = 1/5).

##### Contamination

At T1, all participants were asked if they used any physical activity app to help them do physical activity during the study period (yes; no) and if they answered yes, they were asked to name the app.

#### 2.4.4 App engagement

It was not possible to retrieve actual app use data from NHS Digital as the data were not stored in a way that could link with our trial data. In the T1 questionnaire, intervention participants were asked if they ever used the Active 10 app to track their walking (Yes and I'm still using it; Yes but I'm not using it any more; No). Participants who reported still using it were asked how often they used the app (less than monthly; monthly; fortnightly; weekly; three to four times per week; almost every day or every day). Participants who had ceased using the app were asked how long they had used it for (once; Less than a week; 1 week; 2 weeks; 1 month; 2 months; 3 months). The Digital Behaviour Change Intervention Scale was used to assess engagement with the app.^78^ Participants were asked questions exploring their first use and their most recent use of the app for tracking their walking. Participants were asked how strongly they remembered experiencing feelings from a specified list (interest, fatigue, focus, inattention, distraction, enjoyment, annoyance, pleasure) while using the app (7‐point scale from not at all to extremely), how much time they spent on the app (minutes per day) and what components in the app they remembered using from a specified list (e.g. viewing today's walks). The full set of questions is presented in the Supporting Information.

#### 2.4.5 Physical activity

Physical activity was measured using an activPAL4 micro accelerometer worn on the midline of the thigh. The activPAL was waterproofed in specialist nitrile sleeves and waterproof dressing and was supplied with adhesive for attaching to the thigh. The sampling frequency was programmed at the default setting of 20 Hz. Participants were asked to wear the activPAL continuously for 7 days and to complete log sheets to record when they got up and went to bed across these 7 days and if they removed the device at all. Wearing the activPAL monitor was implemented to assess the feasibility and acceptability of using this outcome measure but this was not a mandatory requirement for participation in the study.

A valid day of wear was defined where the activPAL was worn for the full 24 h and 3 days of valid wear were necessary to be included in the analysis.^91^ The collected data were processed using the Processing PAL software V1.3.^92^ The previously validated default settings were applied,^93^ apart from setting the minimum number of steps to delineate waking to wear time to 200 steps as this was more suited to our patient population. ‘Sleep’ encompassed all time spent in bed and was not subclassified into time spent asleep by biological definitions and/or other time spent in bed.^93^, ^94^ This broad definition included brief periods out of bed inclusive of trips to the bathroom during the night. Heat maps were created to visualise periods of ‘sleep’ versus waking wear time for each participant, at each time‐point. These were compared to participant log sheets to identify possible scenarios where the algorithm may have incorrectly coded ‘sleep’ and waking time.^91^ Where discrepancies were identified (e.g. approximately 1 hour of data was inaccurately coded) corrections were made to reclassify periods of time as ‘sleep’ or wake time as appropriate. Brisk walking was defined as >100 steps per minute as this is the threshold identified to elicit the sufficient walking intensity for MVPA in adults.^95^, ^96^ Total minutes of brisk walking per day were derived from the data as this is the intended primary outcome for the definitive trial. Total minutes walking at any pace was also derived to compare groups at baseline.

#### 2.4.6 Trial experience interviews

Semi‐structured interviews were conducted with participants in both arms by two researchers (FK and SS) to explore experiences of all aspects of trial participation. Engagement with the app and intervention materials were explored with intervention arm participants and are reported briefly here with more detail reported in a separate process evaluation paper (in preparation for publication).

### 2.5 Statistical analysis

The target sample size was based on a minimum of 30 participants per arm required for estimating parameters in a feasibility study^97^, ^98^ and a conservative drop‐out rate of up to 33%. Analyses of all data, including feasibility outcomes and physical activity are descriptive in nature. The sample size calculation for the phase III confirmatory trial was carried out in PASS 2023 Power Analysis and Sample Size Software (2023).

### 2.6 Qualitative analysis

Coding of the interviews was completed by a single researcher (SS) due to time constraints, which impacted the availability of resources for data analysis. However, any uncertainties surrounding participant responses were resolved with a second researcher (FK). Content analysis was used to systematically explore participants' experience of taking part in the study and to quantify responses related to the feasibility and acceptability of study procedures.^99^

### 2.7 Cost‐effectiveness analysis

An exploratory health economic analysis was carried out to provide preliminary cost‐effectiveness estimates and to inform the design of the larger trial and economic analyses. A Markov‐style health economic model was developed that linked increases in physical activity to reductions in cancer and other cause of mortality over a lifetime horizon. The model baseline population was a cohort of individuals with characteristics taken from the APPROACH pilot participant data. Intervention effectiveness data from the trial was converted into metabolic equivalent tasks (METs) to enable stepping at different rates to be represented within a single metric.^100^, ^101^ The model took an NHS perspective on costs and health benefits. Intervention costs were calculated at £62.52 per person based on resources used in the trial. This included printing and posting materials which were costed directly, and nurse time for training and to deliver the intervention, which were costed using PSSRU unit costs.^102^ It was assumed that a mid‐Band 7 hospital nurse would deliver the intervention on an individual basis to 200 patients per year, taking 55 min per patient; whilst a Band 8a hospital nurse would deliver a day of training to ten Band 7 nurses, which would be valid for 3 years. As the Active 10 app is a publicly available app developed by the NHS that exists outside of this intervention, the cost of the app per person was not included as an intervention cost. Quality‐adjusted life years (QALYs) were estimated based on patient‐reported EQ5D scores at baseline, projected over the patient's lifetime. Full details of the model methodology are reported in the Supporting Information.

Probabilistic sensitivity analysis (PSA) was used to estimate mean lifetime costs, QALYs and cost‐effectiveness, with a discount rate of 3.5% applied for costs and QALYs in line with National Institute for Health and Care Excellence (NICE) guidelines.^103^ Expected value of perfect information (EVPI) and perfect parameter information (EVPPI) were estimated.^104^ Structural uncertainties were investigated through scenario analyses.

### 2.8 Ethical considerations

This pilot study was approved by the Yorkshire & The Humber‐South Yorkshire Research Ethics Committee (21/YH/0029) and the Health Research Authority.

## 3 RESULTS

### 3.1 Overview

**FIGURE 1:**
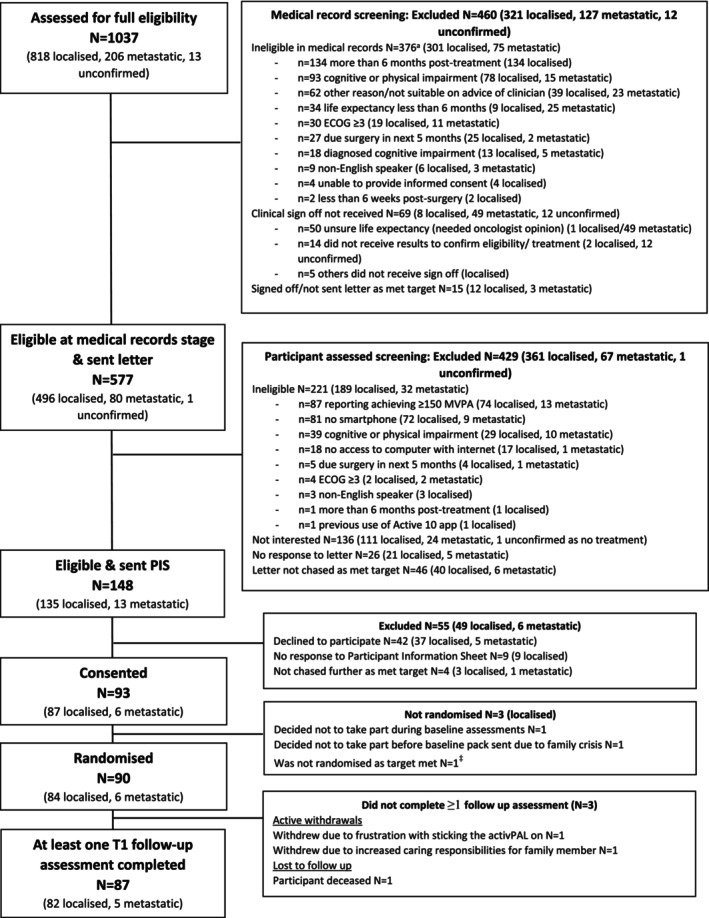
Full Consolidated Standards of Reporting Trials (CONSORT) diagram^†^. ^†^Non‐eligibility reasons could be ≥1. ^‡^This potential participant was informed by telephone that we had met our recruitment target and did not have the sufficient extra resources to include them in the study.

Figure 1 presents the flow of participants from initial screening to enrolment and participation. Of the 1037 patients diagnosed with breast, prostate or colorectal cancer that were assessed for eligibility, 460 (44%) were excluded at the medical records stage. A further 577 patients were sent the initial letter about the study and 429 (74%) were excluded either due to not being interested in participating or based on follow‐up eligibility screening, as outlined below. The Study Information Sheet was sent to 148 patients, with 93 (63%) consenting to participate and 90 (61%) being randomised. Reasons given for declining to participate are presented in the Supporting Information but include finding that the study would be ‘too much’ currently (*n* = 7), that they had too much already going on with treatment (*n* = 6) and that they were too busy (*n* = 4).

### 3.2 Sample characteristics

Table 3 presents sociodemographic and clinical factors, as well as physical activity outcomes at baseline in the sample. Participants were mainly breast (*n* = 36, 40%) and prostate (*n* = 36, 40%) cancer patients, with fewer colorectal cancer patients (*n* = 18, 20%). The mean age of participants was 63 (*SD* = 11, range = 40–85), with a similar number of males (*n* = 47, 52%) to females.

**TABLE 3:** Descriptive statistics for sociodemographic and clinical factors, and physical activity outcomes at baseline.

	Total (*N* = 90)	Intervention (*n* = 44)	Control (*n* = 46)
Age (years): mean (range)	63 (40–85)	63 (40–85)	62 (41–78)
Sex *n* (%)			
Male	47 (52)	22 (50)	25 (54)
Female	43 (48)	22 (50)	21 (46)
Ethnicity *n* (%)			
White	87 (97)	42 (96)	45 (98)
Asian/Asian British	2 (2)	1 (2)	1 (2)
Other^a^	1 (1)	1 (2)	0
Education level *n* (%)			
No formal qualifications	11 (12)	5 (11)	6 (13)
High school/secondary school	31 (34)	15 (34)	16 (35)
AS & A levels or equivalent	13 (14)	8 (18)	5 (11)
Level 4–5 vocational qualifications	12 (13)	2 (5)	10 (22)
Bachelor's degree or equivalent	14 (16)	11 (25)	3 (7)
Master's degree, PGCE, PhD or equivalent	9 (10)	3 (7)	6 (13)
Employment *n* (%)			
Employed full‐time	19 (21)	8 (18)	11 (24)
Employed part‐time	15 (17)	9 (21)	6 (13)
Unemployed	2 (2)	2 (5)	0
Retired	47 (52)	22 (50)	25 (54)
Unable/too ill to work	7 (8)	3 (7)	4 (9)
Marital status *n* (%)			
Married/in a relationship	75 (83)	37 (84)	38 (83)
Single/divorced/separated	8 (9)	3 (7)	5 (11)
Widowed	7 (8)	4 (9)	3 (7)
Living arrangements *n* (%)			
Alone	12 (13)	5 (11)	7 (15)
With partner only	53 (59)	25 (57)	28 (61)
With family	25 (28)	14 (32)	11 (24)
Index of multiple deprivation quintile *n* (%)			
1 (most deprived)	18 (20)	8 (18)	10 (22)
2	15 (17)	6 (14)	9 (20)
3	17 (19)	9 (21)	8 (17)
4	27 (30)	16 (36)	11 (24)
5 (least deprived)	13 (14)	5 (11)	8 (17)
Cancer type *n* (%)			
Breast	36 (40)	18 (41)	18 (39)
Prostate	36 (40)	18 (41)	18 (39)
Colorectal	18 (20)	8 (18)	10 (22)
Cancer stage *n* (%)			
1	29 (32)	15 (34)	14 (30)
2	30 (33)	14 (32)	16 (35)
3	24 (27)	12 (27)	12 (26)
4	7 (8)	3 (7)	4 (9)
Treatment type and stage^b^ *n* (%)			
Surgery *n* (%)			
Underwent surgery	55 (61)	25 (57)	30 (65)
Not had/having surgery	35 (39)	19 (43)	16 (35)
Radiotherapy *n* (%)			
Due to start of radiotherapy	26 (29)	13 (30)	13 (28)
Currently undergoing radiotherapy	2 (2)	0 (0)	2 (4)
Completed radiotherapy	19 (21)	9 (21)	10 (22)
Not had/having radiotherapy	43 (48)	22 (50)	21 (46)
Chemotherapy *n* (%)			
Due to the start chemotherapy	0 (0)	0 (0)	0 (0)
Currently undergoing chemotherapy	10 (11)	3 (7)	7 (15)
Completed chemotherapy	14 (16)	8 (18)	6 (13)
Not had/having chemotherapy	66 (73)	33 (75)	33 (72)
Hormone therapy *n* (%)			
Due to start of hormone therapy	4 (4)	2 (5)	2 (4)
Currently undergoing hormone therapy	39 (43)	20 (46)	19 (41)
Completed hormone therapy	6 (7)	2 (5)	4 (9)
Not had/having hormone therapy	41 (46)	20 (46)	21 (46)
Biological therapy *n* (%)			
Due to the start biological therapy	0 (0)	0 (0)	0 (0)
Currently undergoing biological therapy	7 (8)	3 (7)	4 (9)
Completed biological therapy	2 (2)	2 (5)	0 (0)
Not had/having biological therapy	81 (90)	39 (89)	42 (91)
Months since diagnosis^c^: median (IQR)	5 (4–8)	6 (4–8)	5 (4–7)
Previous cancer diagnoses *n* (%)			
Previously diagnosed with one other cancer^d^	12 (13)	8 (18)	4 (9)
No previous diagnosis of cancer	78 (87)	36 (82)	42 (91)
Comorbid health conditions *n* (%)			
None	28 (31)	13 (30)	15 (33)
1 condition	34 (38)	16 (36)	18 (39)
2+ conditions	28 (31)	15 (34)	13 (28)
Body Mass Index^e^: median (IQR)	28 (25–33)^e^	27 (24–31)^e^	28 (25–34)
Minutes spent brisk walking per week^f^: median (IQR)	181 (116–363)	211 (126–374)	171 (105–255)
Minutes spent walking at any pace per week^f^: median (IQR)	607 (433–784)	626 (493–912)	557 (396–751)
Hours spent sitting per day^f^: median (IQR)	10 (9–11)	10 (9–11)	10 (9–11)
Hours spent standing per day^f^: median (IQR)	3 (3–4)	4 (3–4)	3 (2–4)

Abbreviations: IQR, interquartile range; PGCE, postgraduate certificate of education; PhD, Doctor of Philosophy.

^a^ Participants could specify their ethnicity in the textbox.

^b^ At the date when the baseline assessment pack was sent to the participant.

^c^ At the date of randomisation.

^d^ No participants had received a diagnosis of more than one other cancer.

^e^ When cleaning the data, the BMI of one participant was removed from the analysis due to an outlier weight value that was deemed implausible.

^f^ 88 participants consented to wearing and received the activPAL and 85 participants' activPAL data are reported as three participants did not provide data for the specified sufficient number of days to be included (3 days^36^, ^55^).

### 3.3 Feasibility outcomes

Table 4 presents the results of the feasibility outcomes. The trial procedures were acceptable to participants with no participants giving randomisation as their reason for declining (0%) or withdrawing (0%), high completion rates (>86%) and a 96% participant retention rate. Delivery of the intervention was feasible with 98% of the intervention group receiving the behavioural support call and 96% downloading the app.

**TABLE 4:** Results of the pre‐specified feasibility outcomes.

Feasibility outcomes	Detail of specific outcome	Result
Interest	% of eligible interested/willing to answer eligibility questions	64% (369/577)
Enrolment	% eligible patients enrolled	61% (90/148)
Acceptability of randomisation	% of participants who withdraw post‐randomisation (within 1 week of being informed)	None
	% potential participants who state that randomisation is their reason for declining	None
Feasibility of administering intervention	% of intervention group who received a behavioural support call	98% (43/44)^a^
	% of intervention group who self‐reported downloading the app	96% (42/44)
Acceptability of intervention	% of participants who reported that no aspect of the intervention was useful	None
	% of participants in the intervention group who report using the app for less than a month	5% (2/39^b^)
	% of withdrawals from the intervention group compared to control group	5% (2/44) in intervention group. None in control group
	% of reasons for withdrawal relating to the intervention	None
Retention rate	% of participants, in each group, who complete any of the T1 follow‐up assessment	97% (87/90) completed any follow‐up assessments, and there were similar rates between study groups^c^
Acceptability of outcome assessments	% of participants who consented completed any baseline assessments	100% (91/91^d^)
	Completion rates, in each group, for each of the assessments at baseline and follow‐up	Completion rates were high for all assessments (>86%) and similar between study groups^c^
Willingness to consent to linkage with HES/NCRAS registries for long‐term follow‐up	% of participants who consent for this aspect of the study	100% (90/90)
Acceptability of online assessments	% of participants who required help to complete the questionnaires online	4% (4/90) participants required partial help completing questionnaires
	% of potential participants who give this method of data collection as a reason for declining to participate	None
Acceptability of providing informed consent online	% of participants who give online informed consent as a reason for declining	None
Potential sociodemographic biases in recruitment	Comparison of sample demographics with hospital level data on patients with breast, prostate and colorectal cancer	The sample was similar in terms of age, gender, ethnicity, IMD and cancer type to potentially eligible participants at the recruiting NHS site^e^
Fidelity of intervention delivery in telephone/video calls	Average % of required behaviour change techniques (BCT) covered in intervention calls	96% of the 25 BCTs^f^
Contamination of the control group	% of participants who report using the Active10 app or that a health professional recommended it to them	None^g^

^a^ 97.7% received the first support call (43/44); 88.6% received the second support call (39/44).

^b^ Five intervention participants did not provide data for this outcome. Two participants withdrew several weeks after randomisation, and one did not complete this intervention feedback section of the questionnaire. The further two participants who stated they did not download the app were not shown this question.

^c^ See Table S2.

^d^ Of the 93 participants who consented, two of these were not sent the questionnaire link due to (1) choosing not to take part due to family crisis and (2) as the study had met its recruitment target and did not have sufficient resources to recruit this participant. The other participant completed the baseline questionnaires but withdrew to focus on their treatment, prior to wearing the activPAL.

^e^ See Table 5.

^f^ Most intervention participant calls were coded (42/43), except where there was a recording error (*n* = 1). One participant did not receive any call (*n* = 1). In total, 81 intervention calls (42 first calls and 39 second calls) from 42 participants were included.

^g^ Eight participants from the control group reported using an app to help them with physical activity since beginning their participation in the study and the named apps are presented in Table S3.

#### 3.3.1 Potential sociodemographic biases

Table 5 presents a descriptive comparison of enrolled participants to the aggregate data of the population of people diagnosed with breast, prostate and colorectal cancer at the recruiting NHS Trust. Accounting for the small sample size, enrolled participants were similar in terms of gender, age, ethnicity and IMD quintile. There was a more equal ratio of men to women in this study, but a lower proportion of colorectal cancer patients and a greater proportion of prostate cancer patients were recruited than what would be representative of the population at the site.

**TABLE 5:** Comparison of recruited participants in the pilot study and anonymised aggregate data at hospital site to examine potential recruitment bias.

	Pilot study participants (*N* = 90)	Aggregate site data (*N* = 1072)
Age (years): mean	63	66
Sex, *n* (%)		
Male	47 (52)	435 (41)
Female	43 (48)	637 (59)
Cancer type, *n* (%)		
Breast	36 (40)	405 (38)
Prostate	36 (40)	71 (7)
Colorectal	18 (20)	596 (56)
Ethnicity, *n* (%)		
White	87 (97)	977 (91)
Other	3 (3)	95 (9)
Index of multiple deprivation quintile, *n* (%)		
1	18 (20)	271 (25)
2	15 (17)	225 (21)
3	17 (19)	203 (19)
4	27 (30)	270 (25)
5	13 (14)	103 (10)

### 3.4 App engagement

Two participants withdrew several weeks after randomisation, and one did not complete this intervention feedback section of the questionnaire. Two participants reported not downloading the app and weren't shown any further questions on app use. Out of 39 participants asked if they ever used Active 10 to track their walking, 85% reported using and still using the app (*n* = 33). Out of these participants, 82% reported using it almost every day or every day (*n* = 27) and 18% reported using it three to four times per week (*n* = 6). Fewer participants reported using the app but were no longer using it (*n* = 5, 13%). Of those who said that they had stopped using the app, they reported using the app for the following time periods: 1 week (*n* = 1), 2 weeks (*n* = 1), 1 month (*n* = 1), 2 months (*n* = 1), 3 months (*n* = 1). One participant reported not using the app at all.

Results from the DBCI assessing engagement with the app are presented in Table 6. The mean reported time spent using the app on their first day of use was 19.6 min (range 2–60, SD = 16.0). On their most recent day of use, the mean reported time spent using the was 17.1 min (range 1–60, SD = 16.7). The proportion of app components used was relatively high with participants reporting a mean use of 67.5% of the six key components on their first use of the app and a mean use of 46.3% of the components on their most recent use. The most frequently reported components used by participants at first use of the app were ‘Setting or reviewing targets’ (*n* = 35), ‘Viewing today's walks’ (*n* = 34) and ‘Viewing my walks’ (*n* = 33). On their most recent use of the app, the most frequently reported components used by participants were ‘Viewing today's walks’ (*n* = 34), ‘Viewing my walks’ (*n* = 30) and ‘Viewing rewards’ (*n* = 19). Results of the use of all the available components are presented in Table S4.

**TABLE 6:** Results of the Digital Behaviour Change Intervention Scale assessing engagement with the app (*N* = 38^a^).

	First use ratings, mean (standard deviation)	Last use ratings, mean (standard deviation)
Interest^b^	5.9 (1.0)	5.5 (1.3)
Intrigue^b^	5.3 (1.3)	4.1 (1.9)
Focus^b^	5.7 (1.1)	5.0 (1.7)
Inattention^b^ ^,^ ^c^	6.2 (1.1)	6.2 (1.2)
Distraction^a^ ^,^ ^c^	6.1 (1.1)	6.2 (1.2)
Enjoyment^b^	5.3 (1.3)	5.2 (1.5)
Annoyance^b^ ^,^ ^c^	6.70 (0.65)	6.5 (0.9)
Pleasure^b^	5.1 (1.5)	4.8 (1.8)
How long (in min) do you roughly think that you spent on the app that day?	19.6 (16.0)	17.1 (16.7)
Which of the app's components do you remember visiting (tick all that apply)?^d^	67.5% (28.1)	46.3% (26.7)

^a^ Two participants withdrew several weeks after randomisation, and one did not complete this intervention feedback section of the questionnaire. Two participants reported not downloading the app and weren't shown any further questions on app use.

^b^ Possible range 1–7, with 7 being more engagement.

^c^ Reverse scored.

^d^ Presented as the proportion (%) of components that participants reported using (out of a possible 6 components).

### 3.5 Intended primary outcome: physical activity

Table 7 presents the time spent brisk walking derived from the ActivPAL data for the 82 participants (91%) who provided data at both timepoints (intervention *n* = 40; control *n* = 42). Due to the small sample size, the data are reported for descriptive purposes only, with median and interquartile ranges presented due to the skewness of the data.

**TABLE 7:** Minutes spent brisk walking per week at T0 and T1 (*N* = 82).

Experimental group	T0	T1
Intervention (*n* = 40): median (IQR)	211 (124–378)	276 (179–427)
Control (*n* = 42): median (IQR)	167 (103–269)	192 (91–310)

Abbreviation: IQR, interquartile range.

### 3.6 Main trial power calculation

A total of *N* = 472 participants are required in the larger RCT to detect an effect size of 0.10 h per day of activity at 100 steps per minute, with 90% power and two‐sided 5% significance level, after allowing for up to 10% dropout. This is equivalent to a difference of 6 min per day (42 min per week) between the experimental and control arms. This calculation assumes a standard deviation of 0.20 h per day in the control group with a variance ratio of 1:4 (control:intervention) and is supported by the data observed at both timepoints.

### 3.7 Trial experience interviews

All participants who remained in the study at T1 were approached about taking part in the end of study interviews (*n* = 87; *n* = 2 withdrawn, *n* = 1 deceased). In total, 72 participants completed trial experience interviews. Seven participants provided no reason for declining to participate. Other reasons for not taking part included: not responding to the invitation to interview (*n* = 3); not feeling up to it due to illness‐related side effects (*n* = 2); not feeling confident speaking on the phone (*n* = 1); not feeling like they had much to offer (*n* = 1); being too busy (*n* = 1). Overall participants were generally happy with the trial procedures and a more detailed presentation of the feedback from the qualitative interviews is presented in the Supporting Information. Participants reported mixed feelings about randomisation, with some indicating indifference, and others sharing views that related to their experimental group allocation (Table S5). Participants generally found the completion of study assessments at both timepoints to be acceptable, including wearing the activPAL, completing their body measurements and completing the online questionnaires (Table S6). Most participants expressed that the timing of being approached to take part was reasonable, despite being at different points of their cancer care plan (Table S7). All participants reported a willingness to consent linkage to HES/NCRAS registries for long‐term follow‐up, describing an understanding of why this data would be important and a willingness for the data to be used to help others (Table S8).

### 3.8 Preliminary cost‐effectiveness analysis

**FIGURE 2:**
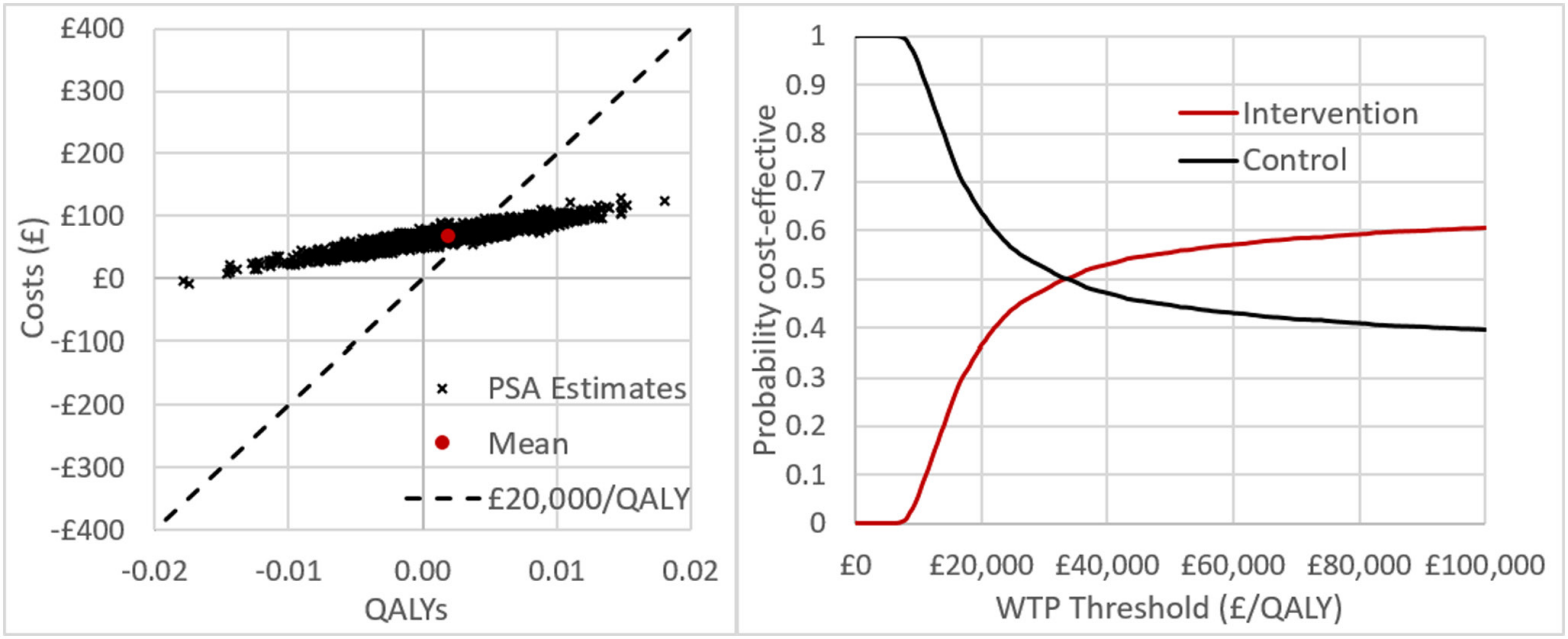
Base‐case analysis cost‐effectiveness results. Left: Spread of probabilistic sensitivity analysis (PSA) results on the cost‐effectiveness plane. Right: Cost‐effectiveness acceptability curve showing the probability the intervention would be cost‐effective at different willingness to pay thresholds.

As expected, there was high uncertainty around the results of the preliminary cost‐effectiveness analysis, given that the feasibility study had not been designed to produce statistically significant effectiveness data. The base‐case health economic analysis suggests that based on the study results, APPROACH would cost £69 (95% credible intervals: £34; £102) and produce 0.0019 (−0.0078; 0.111) QALYs over the lifetime of the average participant compared with no intervention, resulting in an incremental cost‐effectiveness ratio (ICER) of £36,475 and a net monetary benefit of −£31 (−£195; £124) at a willingness to pay threshold of £20,000 per QALY. Whether or not the intervention is cost‐effective is highly uncertain, with a 37% probability that the intervention is cost‐effective at this threshold, and a 63% probability that it is not (Figure 2). EVPI analysis suggests that it could be worth spending up to £18.83 per person likely to be affected by the decision (that is, whether to make the intervention available in the NHS) to remove parameter uncertainty and ensure that the correct decision is made. This is equivalent to a value of approximately £2.8m across all patients diagnosed with breast, prostate and colorectal cancer in the United Kingdom each year. 95% of this value comes from uncertainty around the physical activity intervention effectiveness parameters, particularly changes in stepping at a rate lower than 100 steps per minute.

Given the small sample size in this pilot study, no definitive inferences could be drawn about the effect of the intervention and the durability of the effect. However, scenario analysis indicates that the intervention would have a strong likelihood of being cost‐effective if one or more of the following were true: (a) intervention effectiveness is higher than observed in this small pilot study; (b) duration of intervention effect is longer than 7 years; (c) intervention costs are reduced; (d) NHS resource use is reduced by a small % in the intervention arm; (e) the selected population have a higher baseline mortality risk (e.g. older, more advanced cancer stage or lower baseline physical activity) (see Table S6). A definitive trial should help to inform these parameters more accurately.

## 4 DISCUSSION

The results of this pilot study suggest that an app‐based intervention with brief behavioural support is a feasible and acceptable way to promote brisk walking in people LWBC. The data provided in this study informed the design of a larger, funded, efficacy trial that is powered to determine the impact of the intervention in terms of brisk walking and the cost‐effectiveness of this intervention.

### 4.1 Interest in and acceptability of the study

Previous research reports that people LWBC have a strong desire to receive physical activity advice but are often not provided with it as part of their care.^29^, ^30^ This reported desire is supported by the high interest in taking part in this study (64%) and supports the need to develop physical activity interventions that can be delivered and are accessible to people LWBC. Although many of these interested patients were not enrolled due to exclusion criteria, this was expected and does not undermine the feasibility of the recruitment strategy going forward. Furthermore, participants in this study were similar to the population of people diagnosed with breast, prostate and colorectal cancer at the hospital site. Although there was a higher percentage of white participants than that observed in the aggregate population data, this can be attributed to the small sample size and the location of the pilot site. Additionally, the final sample included proportionally fewer colorectal cancer patients and more prostate cancer patients than the aggregate data. This is likely due to differences in engagement from the clinical staff involved in the care of these patient populations at the single hospital site where the pilot was undertaken. This should be overcome by involving more sites in the confirmatory RCT, as well as monitoring recruitment closely and adapting strategies if needed to increase engagement with clinical staff.

The relatively high enrolment rate (61%) and very high retention rate (97%) show that the trial is feasible. Despite previous research suggesting that randomisation may be unacceptable to some participants, no participants in the present study withdrew directly after randomisation and no potential participants gave randomisation as their reason for declining to take part.^105^, ^106^ Despite some reported disappointment related to control group allocation, the qualitative interviews indicated that participants found randomisation acceptable and being disappointed did not lead to any withdrawals. The outcome assessments were acceptable to participants and there were high completion rates (over 86%) for all assessments at baseline and at follow‐up. This is in line with high retention and assessment completion rates reported in other studies in similar samples with similar follow‐up times and provides a good premise for the potential of sufficient retention rates in a larger trial with more participants and longer follow‐ups.^107^, ^108^ These results informed the power calculation for such a trial and suggested that 472 participants would be required for the larger trial to allow for similar retention rates.

This study recruited participants across the cancer care continuum and included patients with localised and metastatic disease, as well as those still receiving treatment and those within 6 months of radical treatment completion. This inclusive approach was a key consideration at this pilot stage, considering previous research highlighting varying preferences in the timing of the delivery of physical activity interventions.^47^, ^48^ In their qualitative research, Ijsbrandy and colleagues reported how some participants felt that during treatment felt too soon to begin rehabilitation, while others felt that it should have been offered earlier.^109^ Similarly, some participants felt that they would prefer to avoid the hospital after appointments, while others felt it should be integrated into hospital care. Most participants in the current study felt that the timing of being approached was suitable and this aligns with the proposed integration of the intervention into standard NHS care while patients still have contact with their clinical care team. By including a diverse range of participants, we aimed to capture the complexities and challenges associated with delivering a physical activity intervention across different disease contexts and aimed to replicate the implementation of this type of intervention in a realistic setting as closely as possible. This allows for a more inclusive approach that aims to maximise the reach of the intervention to patients at different stages of the cancer care pathway, while the randomisation strategy helps mitigate the potential confounding effects resulting from heterogeneity across treatment and disease stages. When participants are randomly allocated to the intervention and control group, it is assumed that the distribution of patients across these factors is balanced, reducing the risk of confounding bias.^110^

### 4.2 Cost‐effectiveness uncertainty

As expected, preliminary investigations into the cost‐effectiveness of the intervention indicate a high level of uncertainty driven by the physical activity intervention effectiveness parameters. While this is partly due to the small study size, it is compounded by the outcome measures used in the study which are relatively crude (weekly minutes spent walking >100 steps per minute vs weekly minutes spent walking at any pace). The economic analysis converted this measure to METs and used this single metric, as this enabled changes in physical activity to be linked to mortality. However, this required some assumptions about how many METs are represented by each of the primary outcome measures, introducing further uncertainty. Furthermore, there was uncertainty in the physical activity parameters, where the studies used for linking physical activity and mortality in people LWBC included both self‐report and objective measures of physical activity. Previous research suggests that self‐report may significantly underestimate the effect of physical activity on risk reduction, compared to objective measures.^111^ Future research in the planned main trial should adopt a more comprehensive approach to estimating METs with more precision from the accelerometer data, as well as reducing uncertainty by accounting for the potential differences in the measurement of physical activity across studies. Taking these steps will not only improve accuracy in the estimates of physical activity change but will also reduce uncertainties surrounding the cost‐effectiveness of the intervention. Scenario analyses demonstrate the need for a larger RCT, not only to reduce uncertainty around intervention effectiveness but also to capture potential differences in NHS resource use between arms, which could make a large impact on model results. A larger trial would also enable more comprehensive subgroup data to be collected. In the economic modelling, a uniform effect was assumed across all population subgroups due to the small sample size prohibiting the analysis of subgroups. However, data exploration suggested that the intervention may be more cost‐effective in people who are older, with increased morbidities, or less active at baseline. Our EVPI analyses suggest that the value of conducting the larger RCT is likely to be high.

### 4.3 Potential of the intervention

The results suggest that intervention delivery in a future larger‐scale trial can continue as per the pilot study with some refinement and optimisation.^68^ A second paper reports the process evaluation of the intervention as per the Medical Research Council guidance to improve the implementation of complex interventions.^70^ This has allowed for refinement of the intervention for the larger trial, based on both qualitative interview feedback and questionnaire feedback from pilot participants. Adherence to physical activity interventions is a key challenge in healthy populations and this challenge is heightened in people LWBC due to several factors including treatment effects, fatigue and comorbid conditions.^112^ However, adherence can be improved with well‐designed physical activity interventions that employ behaviour‐change techniques and encourage habit formation.^113^ Supporting the intervention design, participant engagement with the app was very high with most of the intervention participants reporting that they were still using Active 10 after 1 month (95%). This may be attributed to the promotion of habit formation in the intervention, inviting an exploration of the habit scores in a larger scale trial with a longer follow‐up. The results of the DBCI also demonstrated good engagement with the app and participants reported a high proportion of use of the app's key features and demonstrated continuing to use these during their most recent use of the app (e.g. viewing today's walks).

While the intervention demonstrates potential for improving MVPA, it is important to note that device‐based measures of physical activity suggest that participants in this study were already relatively active. Participants were screened before taking part, and this already higher level of MVPA could be attributed to discrepancies in device‐based versus self‐reported recall of physical activity.^114^, ^115^, ^116^ While people typically perceive their participation in physical activity in relation to a total duration of purposeful physical activity (e.g. 30 min of walking), accelerometers can fragment the movement behaviours further (e.g. 5 min of brisk walking during a 30‐min duration walk).^115^, ^117^, ^118^ However, the screening tool employed was validated and appropriate for our clinical population, given that it would not be feasible to objectively measure physical activity at this early stage of recruitment and the eligibility questions mirrored the physical activity recommendations, which are based on self‐report.^72^, ^73^, ^115^ In any case, the intervention group demonstrated a larger increase in the primary outcome than the control group when using the device‐based measure of physical activity. This supports the appropriate use of the activPAL to accurately capture our primary outcome in the phase III trial physical activity measurement.

### 4.4 Strengths and limitations

Strengths of this study included that the sample was similar in terms of gender, ethnicity, age and IMD quintile to the population diagnosed with the relevant cancers at the participating hospital site. This intervention was designed and developed based on data collected from people LWBC and drew on behavioural change theory and habit theory to promote brisk walking.^45^, ^64^, ^67^ The concept development considered the practical implementation of the intervention beyond the trial and therefore is a low‐cost, scalable, time‐effective intervention that could be incorporated into routine care in people LWBC and potentially delivered by cancer specialist nurses.^63^ The use of accelerometers to measure physical activity is favourable to self‐report and the activPAL has shown strong reliability and validity in the measurement of walking at different paces.^119^

Limitations of this study include that participants were recruited from a single site, and thus may not be demographically and ethnically representative of the larger population of people LWBC. The larger, confirmatory trial will recruit from multiple sites. This study also required participants to have a smartphone which may have excluded participants of older age and lower socioeconomic position.^120^ Despite this being a reported exclusion reason for 81 individuals (although non‐eligibility reasons could be >1 and therefore some individuals may have been ineligible on other criteria as well), smartphone ownership is still increasing.^37^ Particularly given the lasting effects of the COVID‐19 pandemic on remote delivery of cancer care, an app‐based behavioural intervention such as APPROACH may be preferable as it can support a wide population while still incorporating the proposed benefit of personal contact in effectively changing behaviour.^39^, ^121^ It is however important to note that app usage was collected via self‐report which may be impacted by recall errors and recency biases.^122^ However, it was not possible to access direct apps using analytics. Another limitation of the present study was the limited availability of resources which prevented the involvement of additional coders in the qualitative interview analysis. Despite this constraint, it is widely acknowledged that including qualitative data in pilot studies provides important insights that would have been otherwise overlooked if the data had been excluded completely due to this limitation.^123^ Lastly, as expected, the health economic analysis was limited by the uncertainty surrounding the economic modelling, due to the small study size and crude effectiveness data collected.

## 5 CONCLUSION

This pilot study demonstrates that the APPROACH intervention is feasible and acceptable to people living with and beyond a diagnosis of breast, prostate or colorectal cancer. This supports the progression of a confirmatory phase III trial with a larger sample to determine the clinical effectiveness of the intervention and to evaluate its cost‐effectiveness.

## AUTHOR CONTRIBUTIONS

**Phillippa Lally:** Conceptualization (equal); data curation (equal); formal analysis (equal); funding acquisition (equal); investigation (equal); methodology (equal); writing – review and editing (equal). **Fiona Kennedy:** Conceptualization (equal); data curation (equal); formal analysis (equal); investigation (equal); methodology (equal); writing – review and editing (equal). **Susan Smith:** Data curation (equal); investigation (equal); methodology (equal); writing – original draft (equal); writing – review and editing (equal). **Rebecca J. Beeken:** Conceptualization (equal); funding acquisition (equal); writing – review and editing (equal). **Caroline Buck:** Investigation (equal); writing – review and editing (equal). **Chloe Thomas:** Conceptualization (equal); formal analysis (equal); funding acquisition (equal); methodology (equal); writing – review and editing (equal). **Nicholas Counsell:** Conceptualization (equal); formal analysis (equal); methodology (equal); writing – review and editing (equal). **Lynda Wyld:** Writing – review and editing (equal). **Charlene Martin:** Data curation (equal); investigation (equal); methodology (equal); writing – review and editing (equal). **Sarah Williams:** Data curation (equal); methodology (equal); writing – review and editing (equal). **Anna Roberts:** Conceptualization (equal); funding acquisition (equal); writing – review and editing (equal). **Diana M. Greenfield:** Conceptualization (equal); funding acquisition (equal); writing – review and editing (equal). **Jacqui Gath:** Funding acquisition (supporting); writing – review and editing (equal). **Henry W. W. Potts:** Conceptualization (equal); funding acquisition (equal); writing – review and editing (equal). **Nicholas Latimer:** Conceptualization (equal); funding acquisition (equal); writing – review and editing (equal). **Lee Smith:** Conceptualization (equal); writing – review and editing (equal). **Abi Fisher:** Conceptualization (equal); funding acquisition (equal); writing – review and editing (equal).

## CONFLICT OF INTEREST STATEMENT

HWWP has paid consultancy roles for two digital health companies, Thrive Therapeutic Software Limited and Flo Health UK Limited. He has a PhD student who works at and has fees paid by AstraZeneca, and another who works at and has fees paid by Patients Know Best.

## ETHICAL APPROVAL

This study was approved by the Yorkshire & The Humber‐South Yorkshire Research Ethics Committee (21/YH/0029) and the Health Research Authority.

## CLINICAL TRIAL REGISTRATION NUMBER

ISRCTN registry, ISRCT N1806 3498. Registered 16 April 2021.

## Supporting information


Data S1.

## Data Availability

The data sets generated during and/or analysed during this study are available from the corresponding author on reasonable request.

## References

[1] Cancer Research UK . Cancer Mortality Statistics. Accessed March 03, 2023. https://www.cancerresearchuk.org/health‐professional/cancer‐statistics/mortality

[2] Cancer Research UK . Cancer survival statistics for all cancers combined 2023. Accessed September 29, 2023. https://www.cancerresearchuk.org/health‐professional/cancer‐statistics/survival/all‐cancers‐combined

[3] Cancer Research UK . Cancer incidence for all cancers combined 2023. Accessed October 01, 2023. https://www.cancerresearchuk.org/health‐professional/cancer‐statistics/incidence/all‐cancers‐combined#ref‐6

[4] Frikkel J , Götte M , Beckmann M , et al. Fatigue, barriers to physical activity and predictors for motivation to exercise in advanced cancer patients. BMC Palliat Care. 2020;19(1):43. doi:10.1186/s12904-020-00542-z 32234027 PMC7110817

[5] Maass S , Boerman LM , Verhaak PFM , Du J , de Bock GH , Berendsen AJ . Long‐term psychological distress in breast cancer survivors and their matched controls: a cross‐sectional study. Maturitas. 2019;130:6‐12. doi:10.1016/j.maturitas.2019.09.003 31706438

[6] Demoor‐Goldschmidt C , de Vathaire F . Review of risk factors of secondary cancers among cancer survivors. Br J Radiol. 2018;92:20180390. doi:10.1259/bjr.20180390 30102558 PMC6435077

[7] Annunziata MA , Muzzatti B , Flaiban C , et al. Long‐term quality of life profile in oncology: a comparison between cancer survivors and the general population. Support Care Cancer. 2018;26(2):651‐656. doi:10.1007/s00520-017-3880-8 28918552

[8] Roy S , Vallepu S , Barrios C , Hunter K . Comparison of comorbid conditions between cancer survivors and age‐matched patients without cancer. J Clin Med Res. 2018;10(12):911‐919. doi:10.14740/jocmr3617w 30425764 PMC6225860

[9] Neo J , Fettes L , Gao W , Higginson IJ , Maddocks M . Disability in activities of daily living among adults with cancer: a systematic review and meta‐analysis. Cancer Treat Rev. 2017;61:94‐106. doi:10.1016/j.ctrv.2017.10.006 29125982

[10] World Cancer Research Fund/American Institute for Cancer Research . Continuous Update Project Expert Report. Diet, nutrition, physical activity and breast cancer. 2018.

[11] Salam A , Woodman A , Chu A , et al. Effect of post‐diagnosis exercise on depression symptoms, physical functioning and mortality in breast cancer survivors: a systematic review and meta‐analysis of randomized control trials. Cancer Epidemiol. 2022;77:102111. doi:10.1016/j.canep.2022.102111 35091272

[12] Cariolou M , Abar L , Aune D , et al. Postdiagnosis recreational physical activity and breast cancer prognosis: global Cancer update Programme (CUP global) systematic literature review and meta‐analysis. Int J Cancer. 2023;152(4):600‐615. doi:10.1002/ijc.34324 36279903 PMC10091720

[13] Friedenreich CM , Stone CR , Cheung WY , Hayes SC . Physical activity and mortality in cancer survivors: a systematic review and meta‐analysis. JNCI Cancer Spectr. 2020;4(1):pkz080. doi:10.1093/jncics/pkz080 32337494 PMC7050161

[14] Buffart LM , Kalter J , Sweegers MG , et al. Effects and moderators of exercise on quality of life and physical function in patients with cancer: an individual patient data meta‐analysis of 34 RCTs. Cancer Treat Rev. 2017;52:91‐104. doi:10.1016/j.ctrv.2016.11.010 28006694

[15] Christensen JF , Simonsen C , Hojman P . Exercise training in cancer control and treatment. Compr Physiol. 2018;9(1):165‐205. doi:10.1002/cphy.c180016 30549018

[16] McTiernan A , Friedenreich CM , Katzmarzyk PT , et al. Physical activity in cancer prevention and survival: a systematic review. Med Sci Sports Exerc. 2019;51(6):1252‐1261. doi:10.1249/mss.0000000000001937 31095082 PMC6527123

[17] Cancer Research UK . Cancer incidence for common cancers 2023. Accessed October 01, 2023. https://www.cancerresearchuk.org/health‐professional/cancer‐statistics/incidence/common‐cancers‐compared#~:text=Breast%20cancer%20is%20the%20most,%2C%20and%20bowel%20(11%25)

[18] Geidl W , Schlesinger S , Mino E , Miranda L , Pfeifer K . Dose–response relationship between physical activity and mortality in adults with noncommunicable diseases: a systematic review and meta‐analysis of prospective observational studies. Int J Behav Nutr Phys Act. 2020;17(1):109. doi:10.1186/s12966-020-01007-5 32843054 PMC7448980

[19] Benke IN , Leitzmann MF , Behrens G , Schmid D . Physical activity in relation to risk of prostate cancer: a systematic review and meta‐analysis. Ann Oncol. 2018;29(5):1154‐1179. doi:10.1093/annonc/mdy073 29788165

[20] Qiu S , Jiang C , Zhou L . Physical activity and mortality in patients with colorectal cancer: a meta‐analysis of prospective cohort studies. Eur J Cancer Prev. 2020;29(1):15‐26. doi:10.1097/cej.0000000000000511 30964753

[21] Schmid D , Leitzmann MF . Association between physical activity and mortality among breast cancer and colorectal cancer survivors: a systematic review and meta‐analysis. Ann Oncol. 2014;25(7):1293‐1311. doi:10.1093/annonc/mdu012 24644304

[22] Mishra SI , Scherer RW , Geigle PM , et al. Exercise interventions on health‐related quality of life for cancer survivors. Cochrane Database Syst Rev. 2012;8:CD007566. doi:10.1002/14651858.CD007566.pub2 PMC7387117 22895961

[23] Mishra SI , Scherer RW , Snyder C , Geigle PM , Berlanstein DR , Topaloglu O . Exercise interventions on health‐related quality of life for people with cancer during active treatment. Cochrane Database Syst Rev. 2012;8:CD008465. doi:10.1002/14651858.CD008465.pub2 PMC7389071 22895974

[24] Aune D , Markozannes G , Abar L , et al. Physical activity and health‐related quality of life in women with breast cancer: a meta‐analysis. JNCI Cancer Spectrum. 2022;6(6):pkac072. doi:10.1093/jncics/pkac072 36474321 PMC9727071

[25] World Cancer Research Fund . Survivors of breast and other cancers. 2018.

[26] World Cancer Research Fund . Move More 2023. Accessed August 01, 2023. https://www.wcrf‐uk.org/preventing‐cancer/our‐cancer‐prevention‐recommendations/move‐more/

[27] Du Y , Liu B , Sun Y , Snetselaar LG , Wallace RB , Bao W . Trends in adherence to the physical activity guidelines for Americans for aerobic activity and time spent on sedentary behavior among US adults, 2007 to 2016. JAMA Netw Open. 2019;2(7):e197597. doi:10.1001/jamanetworkopen.2019.7597 31348504 PMC6661709

[28] Independent Cancer Taskforce . Achieving World‐Class Cancer Outcomes – A Strategy for England 2015–2020. 2015. Accessed July 15, 2021. https://www.cancerresearchuk.org/sites/default/files/achieving_world‐class_cancer_outcomes_‐_a_strategy_for_england_2015‐2020.pdf

[29] Koutoukidis DA , Beeken RJ , Lopes S , Knobf MT , Lanceley A . Attitudes, challenges and needs about diet and physical activity in endometrial cancer survivors: a qualitative study. Eur J Cancer Care. 2016;26:e12531. doi:10.1111/ecc.12531 27324208

[30] Smith L , Croker H , Fisher A , Williams K , Wardle J , Beeken RJ . Cancer survivors' attitudes towards and knowledge of physical activity, sources of information, and barriers and facilitators of engagement: a qualitative study. Eur J Cancer Care (Engl). 2017;26(4):e12641. doi:10.1111/ecc.12641 28135016

[31] Smith SK , Wiltshire G , Brown FF , et al. ‘You're kind of left to your own devices’: a qualitative focus group study of patients with breast, prostate or blood cancer at a hospital in the south West of England, exploring their engagement with exercise and physical activity during cancer treatment and in the months following standard care. BMJ Open. 2022;12(3):e056132. doi:10.1136/bmjopen-2021-056132 PMC8961148 35351718

[32] Cantwell M , Walsh D , Furlong B , et al. Physical activity across the cancer journey: experiences and recommendations from people living with and beyond cancer. Phys Ther. 2019;100(3):575‐585. doi:10.1093/ptj/pzz136 31588506

[33] Koutoukidis DA , Lopes S , Fisher A , Williams K , Croker H , Beeken RJ . Lifestyle advice to cancer survivors: a qualitative study on the perspectives of health professionals. BMJ Open. 2018;8(3):e020313. doi:10.1136/bmjopen-2017-020313 PMC5875617 29593021

[34] Haussmann A , Gabrian M , Ungar N , et al. What hinders healthcare professionals in promoting physical activity towards cancer patients? The influencing role of healthcare professionals' concerns, perceived patient characteristics and perceived structural factors. Eur J Cancer Care (Engl). 2018;27(4):e12853. doi:10.1111/ecc.12853 29741781

[35] Robinson R , Crank H , Humphreys H , Fisher P , Greenfield DM . Time to embed physical activity within usual care in cancer services: a qualitative study of cancer healthcare professionals' views at a single centre in England. Disabil Rehabil. 2022;45:1‐9. doi:10.1080/09638288.2022.2134468 36369938

[36] Alfano CM , Bluethmann SM , Tesauro G , et al. NCI funding trends and priorities in physical activity and energy balance research among cancer survivors. J Natl Cancer Inst. 2016;108(1):djv285. doi:10.1093/jnci/djv285 26547926

[37] Ofcom . Technology Tracker 2022. 2022.

[38] Cancer Research UK . Cancer Incidence by Age 2023. Accessed May 24, 2023. https://www.cancerresearchuk.org/health‐professional/cancer‐statistics/incidence/age%23heading‐Zero

[39] Khoo S , Mohbin N , Ansari P , Al‐Kitani M , Müller AM . mHealth interventions to address physical activity and sedentary behavior in cancer survivors: a systematic review. Int J Environ Res Public Health. 2021;18(11):5798. doi:10.3390/ijerph18115798 34071342 PMC8198944

[40] Wallbank G , Sherrington C , Hassett L , et al. Acceptability and feasibility of an online physical activity program for women over 50: a pilot trial. Transl Behav Med. 2022;12(2):225‐236. doi:10.1093/tbm/ibab161 35020938

[41] Roberts AL , Fisher A , Smith L , Heinrich M , Potts HWW . Digital health behaviour change interventions targeting physical activity and diet in cancer survivors: a systematic review and meta‐analysis. J Cancer Surviv. 2017;11(6):704‐719. doi:10.1007/s11764-017-0632-1 28779220 PMC5671545

[42] Kang H , Moon M . Effects of digital physical activity interventions for breast cancer patients and survivors: a systematic review and meta‐analysis. Healthc Inform Res. 2023;29(4):352‐366. doi:10.4258/hir.2023.29.4.352 37964457 PMC10651404

[43] Ester M , McNeely ML , McDonough MH , Culos‐Reed SN . A survey of technology literacy and use in cancer survivors from the Alberta Cancer exercise program. Digit Health. 2021;7:20552076211033426. doi:10.1177/20552076211033426 34422280 PMC8370891

[44] Short CE , Finlay A , Sanders I , Maher C . Development and pilot evaluation of a clinic‐based mHealth app referral service to support adult cancer survivors increase their participation in physical activity using publicly available mobile apps. BMC Health Serv Res. 2018;18(1):27. doi:10.1186/s12913-017-2818-7 29338722 PMC5771037

[45] Roberts AL , Potts HW , Koutoukidis DA , Smith L , Fisher A . Breast, prostate, and colorectal cancer survivors' experiences of using publicly available physical activity mobile apps: qualitative study. JMIR Mhealth Uhealth. 2019;7(1):e10918. doi:10.2196/10918 30609982 PMC6329432

[46] Martín Payo R , Harris J , Armes J . Prescribing fitness apps for people with cancer: a preliminary assessment of content and quality of commercially available apps. J Cancer Surviv. 2019;13(3):397‐405. doi:10.1007/s11764-019-00760-2 31030308

[47] Phillips SM , Conroy DE , Keadle SK , et al. Breast cancer survivors' preferences for technology‐supported exercise interventions. Support Care Cancer. 2017;25(10):3243‐3252. doi:10.1007/s00520-017-3735-3 28470368 PMC5832636

[48] Elshahat S , Treanor C , Donnelly M . Factors influencing physical activity participation among people living with or beyond cancer: a systematic scoping review. Int J Behav Nutr Phys Act. 2021;18(1):50. doi:10.1186/s12966-021-01116-9 33823832 PMC8025326

[49] Gildea GC , Spence RR , Jones TL , et al. Barriers, facilitators, perceptions and preferences influencing physical activity participation, and the similarities and differences between cancer types and treatment stages – a systematic rapid review. Prev Med Rep. 2023;34:102255. doi:10.1016/j.pmedr.2023.102255 37273528 PMC10236469

[50] Smaradottir A , Smith AL , Borgert AJ , Oettel KR . Are we on the same page? Patient and provider perceptions about exercise in cancer care: a focus group study. J Natl Compr Canc Netw. 2017;15(5):588‐594. 28476738 10.6004/jnccn.2017.0061

[51] Rock CL , Thomson CA , Sullivan KR , et al. American cancer society nutrition and physical activity guideline for cancer survivors. CA Cancer J Clin. 2022;72(3):230‐262. doi:10.3322/caac.21719 35294043

[52] Yang L , Morielli AR , Heer E , et al. Effects of exercise on cancer treatment efficacy: a systematic review of preclinical and clinical studies. Cancer Res. 2021;81(19):4889‐4895. 34215623 10.1158/0008-5472.CAN-21-1258 PMC9397632

[53] Bland KA , Zadravec K , Landry T , Weller S , Meyers L , Campbell KL . Impact of exercise on chemotherapy completion rate: a systematic review of the evidence and recommendations for future exercise oncology research. Crit Rev Oncol Hematol. 2019;136:79‐85. 30878132 10.1016/j.critrevonc.2019.02.005

[54] Hudock NL , Mani K , Khunsriraksakul C , et al. Future trends in incidence and long‐term survival of metastatic cancer in the United States. Commun Med. 2023;3(1):76. doi:10.1038/s43856-023-00304-x 37244961 PMC10224927

[55] Sanft T , Denlinger CS , Armenian S , et al. NCCN guidelines insights: survivorship, version 2.2019. J Natl Compr Canc Netw. 2019;17(7):784‐794. doi:10.6004/jnccn.2019.0034 31319383 PMC7094216

[56] Oldervoll LM , Loge JH , Lydersen S , et al. Physical exercise for cancer patients with advanced disease: a randomized controlled trial. Oncologist. 2011;16(11):1649‐1657. doi:10.1634/theoncologist.2011-0133 21948693 PMC3233301

[57] Scott JM , Iyengar NM , Nilsen TS , et al. Feasibility, safety, and efficacy of aerobic training in pretreated patients with metastatic breast cancer: a randomized controlled trial. Cancer. 2018;124(12):2552‐2560. doi:10.1002/cncr.31368 29624641 PMC5990447

[58] Avancini A , Sartori G , Gkountakos A , et al. Physical activity and exercise in lung cancer care: will promises be fulfilled? Oncologist. 2020;25(3):e555‐e569. doi:10.1634/theoncologist.2019-0463 32162811 PMC7066706

[59] Wilk M , Kepski J , Kepska J , Casselli S , Szmit S . Exercise interventions in metastatic cancer disease: a literature review and a brief discussion on current and future perspectives. BMJ Support Palliat Care. 2020;10(4):404‐410. doi:10.1136/bmjspcare-2020-002487 32943468

[60] Wong JN , McAuley E , Trinh L . Physical activity programming and counseling preferences among cancer survivors: a systematic review. Int J Behav Nutr Phys Act. 2018;15(1):48. doi:10.1186/s12966-018-0680-6 29879993 PMC5992647

[61] Joseph R , Hart NH , Bradford N , et al. Diet and exercise advice and referrals for cancer survivors: an integrative review of medical and nursing perspectives. Support Care Cancer. 2022;30(10):8429‐8439. doi:10.1007/s00520-022-07152-w 35616734 PMC9512858

[62] Monteiro‐Guerra F , Signorelli GR , Rivera‐Romero O , Dorronzoro‐Zubiete E , Caulfield B . Breast cancer survivors' perspectives on motivational and personalization strategies in mobile app‐based physical activity coaching interventions: qualitative study. JMIR Mhealth Uhealth. 2020;8(9):e18867. doi:10.2196/18867 32955446 PMC7536602

[63] Roberts AL , Potts HWW , Stevens C , Lally P , Smith L , Fisher A . Cancer specialist nurses' perspectives of physical activity promotion and the potential role of physical activity apps in cancer care. J Cancer Surviv. 2019;13:815‐828. doi:10.1007/s11764-019-00801-w 31475306 PMC6828618

[64] Lally P , Gardner B . Promoting habit formation. Health Psychol Rev. 2013;7(sup1):S137‐S158. doi:10.1080/17437199.2011.603640

[65] Howlett N , Trivedi D , Troop NA , Chater AM . Are physical activity interventions for healthy inactive adults effective in promoting behavior change and maintenance, and which behavior change techniques are effective? A systematic review and meta‐analysis. Transl Behav Med. 2019;9(1):147‐157. doi:10.1093/tbm/iby010 29506209 PMC6305562

[66] Bourke L , Homer KE , Thaha MA , et al. Interventions for promoting habitual exercise in people living with and beyond cancer. Cochrane Database Syst Rev. 2013;9:CD010192. 10.1002/14651858.CD010192.pub2 24065550

[67] Michie S , Richardson M , Johnston M , et al. The behavior change technique taxonomy (v1) of 93 hierarchically clustered techniques: building an international consensus for the reporting of behavior change interventions. Ann Behav Med. 2013;46(1):81‐95. doi:10.1007/s12160-013-9486-6 23512568

[68] Lally P , Miller N , Roberts A , et al. An app with brief behavioural support to promote physical activity after a cancer diagnosis (APPROACH): study protocol for a pilot randomised controlled trial. Pilot Feasibility Stud. 2022;8(1):74. doi:10.1186/s40814-022-01028-w 35351187 PMC8961486

[69] Craig P , Dieppe P , Macintyre S , Michie S , Nazareth I , Petticrew M . Developing and evaluating complex interventions: the new Medical Research Council guidance. Br Med J. 2008;337(7676):a1655. doi:10.1136/bmj.a1655 18824488 PMC2769032

[70] Skivington K , Matthews L , Simpson SA , et al. A new framework for developing and evaluating complex interventions: update of Medical Research Council guidance. BMJ. 2021;374:n2061. doi:10.1136/bmj.n2061 34593508 PMC8482308

[71] Somers TJ , Abernethy AP , Edmond SN , et al. A pilot study of a mobile health pain coping skills training protocol for patients with persistent cancer pain. J Pain Symptom Manage. 2015;50(4):553‐558. doi:10.1016/j.jpainsymman.2015.04.013 26025279 PMC4592787

[72] Elley CR , Kerse NM , Arroll B . Why target sedentary adults in primary health care? Baseline results from the Waikato heart, health, and activity study. Prev Med. 2003;37(4):342‐348. doi:10.1016/S0091-7435(03)00142-7 14507491

[73] Rose SB , Elley CR , Lawton BA , Dowell AC . A single question reliably identifies physically inactive women in primary care. N Z Med J. 2008;121(1268):U2897. 18256708

[74] Harris PA , Taylor R , Minor BL , et al. The REDCap consortium: building an international community of software partners. J Biomed Inform. 2019;95:9. doi:10.1016/j.jbi.2019.103208 PMC7254481 31078660

[75] Harris PA , Taylor R , Thielke R , Payne J , Gonzalez N , Conde JG . Research electronic data capture (REDCap) – a metadata‐driven methodology and workflow process for providing translational research informatics support. J Biomed Inform. 2009;42(2):377‐381. 18929686 10.1016/j.jbi.2008.08.010 PMC2700030

[76] Byford S , Leese M , Knapp M , et al. Comparison of alternative methods of collection of service use data for the economic evaluation of health care interventions. Health Econ. 2007;16(5):531‐536. doi:10.1002/hec.1175 17001749

[77] Foster C , Breckons M , Cotterell P , et al. Cancer survivors' self‐efficacy to self‐manage in the year following primary treatment. J Cancer Surviv. 2015;9(1):11‐19. doi:10.1007/s11764-014-0384-0 25028218 PMC4341005

[78] Perski O , Blandford A , Garnett C , Crane D , West R , Michie S . A self‐report measure of engagement with digital behavior change interventions (DBCIs): development and psychometric evaluation of the “DBCI engagement scale”. Transl Behav Med. 2019;10(1):267‐277. doi:10.1093/tbm/ibz039 PMC8411853 30927357

[79] Herdman M , Gudex C , Lloyd A , et al. Development and preliminary testing of the new five‐level version of EQ‐5D (EQ‐5D‐5L). Qual Life Res. 2011;20(10):1727‐1736. doi:10.1007/s11136-011-9903-x 21479777 PMC3220807

[80] Yellen SB , Cella DF , Webster K , Blendowski C , Kaplan E . Measuring fatigue and other anemia‐related symptoms with the functional assessment of cancer therapy (FACT) measurement system. J Pain Symptom Manage. 1997;13(2):63‐74. doi:10.1016/S0885-3924(96)00274-6 9095563

[81] Cella DF , Tulsky DS , Gray G , et al. The functional assessment of Cancer‐Therapy Scale – development and validation of the general measure. J Clin Oncol. 1993;11(3):570‐579. 8445433 10.1200/JCO.1993.11.3.570

[82] Spitzer RL , Kroenke K , Williams JB , Löwe B . A brief measure for assessing generalized anxiety disorder: the GAD‐7. Arch Intern Med. 2006;166(10):1092‐1097. doi:10.1001/archinte.166.10.1092 16717171

[83] Godin G , Shephard RJ . A simple method to assess exercise behavior in the community. Can J Appl Sport Sci. 1985;10(3):141‐146. 4053261

[84] Horne JA , Ostberg O . A self‐assessment questionnaire to determine morningness‐eveningness in human circadian rhythms. Int J Chronobiol. 1976;4(2):97‐110. 1027738

[85] Haas BK , Northam S . Measuring self‐efficacy: development of the physical activity assessment inventory. South Online J Nurs Res. 2010;10(4):35‐51.

[86] Kroenke K , Spitzer RL , Williams JB . The PHQ‐9: validity of a brief depression severity measure. J Gen Intern Med. 2001;16(9):606‐613. doi:10.1046/j.1525-1497.2001.016009606.x 11556941 PMC1495268

[87] Buysse DJ , Reynolds CF , Monk TH , Berman SR , Kupfer DJ . The Pittsburgh sleep quality index – a new instrument for psychiatric practice and research. Psychiatry Res. 1989;28(2):193‐213. doi:10.1016/0165-1781(89)90047-4 2748771

[88] Gardner B , Abraham C , Lally P , de Bruijn GJ . Towards parsimony in habit measurement: testing the convergent and predictive validity of an automaticity subscale of the self‐report habit index. Int J Behav Nutr Phys Act. 2012;9:102. doi:10.1186/1479-5868-9-102 22935297 PMC3552971

[89] Stamatakis E , Kelly P , Strain T , Murtagh EM , Ding D , Murphy MH . Self‐rated walking pace and all‐cause, cardiovascular disease and cancer mortality: individual participant pooled analysis of 50,225 walkers from 11 population British cohorts. Br J Sports Med. 2018;52(12):761‐768. doi:10.1136/bjsports-2017-098677 29858463

[90] Ministry of Housing CLG . The English Indices of Deprivation 2019 (IoD2019). Ministry of Housing CLG; 2019.

[91] Edwardson CL , Winkler EAH , Bodicoat DH , et al. Considerations when using the activPAL monitor in field‐based research with adult populations. J Sport Health Sci. 2017;6(2):162‐178. doi:10.1016/j.jshs.2016.02.002 30356601 PMC6188993

[92] Edwardson C , Ette S . Processing PAL – V1.3 06092019 2019. https://github.com/UOL‐COLS/ProcessingPAL/releases/tag/v1.3.

[93] Winkler EA , Bodicoat DH , Healy GN , et al. Identifying adults' valid waking wear time by automated estimation in activPAL data collected with a 24 h wear protocol. Physiol Meas. 2016;37(10):1653‐1668. doi:10.1088/0967-3334/37/10/1653 27652827

[94] Hamer M , Stamatakis E , Chastin S , et al. Feasibility of measuring sedentary time using data from a thigh‐worn accelerometer. Am J Epidemiol. 2020;189(9):963‐971. doi:10.1093/aje/kwaa047 32219368 PMC7443760

[95] Tudor‐Locke C , Aguiar EJ , Han H , et al. Walking cadence (steps/min) and intensity in 21–40 year olds: CADENCE‐adults. Int J Behav Nutr Phys Act. 2019;16(1):8. doi:10.1186/s12966-019-0769-6 30654810 PMC6337834

[96] Tudor‐Locke C , Ducharme SW , Aguiar EJ , et al. Walking cadence (steps/min) and intensity in 41 to 60‐year‐old adults: the CADENCE‐adults study. Int J Behav Nutr Phys Act. 2020;17(1):137. doi:10.1186/s12966-020-01045-z 33168018 PMC7654058

[97] Browne RH . On the use of a pilot sample for sample‐size determination. Stat Med. 1995;14(17):1933‐1940. doi:10.1002/sim.4780141709 8532986

[98] Lancaster GA , Dodd S , Williamson PR . Design and analysis of pilot studies: recommendations for good practice. J Eval Clin Pract. 2004;10(2):307‐312. doi:10.1111/j.0.2002.384.doc.x 15189396

[99] Schreier M . Qualitative content analysis in practice. Sage Publications Ltd; 2012:1‐280.

[100] Tudor‐Locke C , Han H , Aguiar EJ , et al. How fast is fast enough? Walking cadence (steps/min) as a practical estimate of intensity in adults: a narrative review. Br J Sports Med. 2018;52(12):776‐788. doi:10.1136/bjsports-2017-097628 29858465 PMC6029645

[101] Jetté M , Sidney K , Blümchen G . Metabolic equivalents (METS) in exercise testing, exercise prescription, and evaluation of functional capacity. Clin Cardiol. 1990;13(8):555‐565. doi:10.1002/clc.4960130809 2204507

[102] Unit Costs of Health and Social Care Personal Social Services Research Unit (PSSRU). 2021. Accessed November 1. https://www.pssru.ac.uk/project‐pages/unit‐costs/unit‐costs‐of‐health‐and‐social‐care‐2021/

[103] National Institute of Health and Care Excellence (NICE) . Guide to the methods of technology appraisal. 2013. 27905712

[104] Strong M , Oakley JE , Brennan A . Estimating multiparameter partial expected value of perfect information from a probabilistic sensitivity analysis sample: a nonparametric regression approach. Med Decis Making. 2014;34(3):311‐326. doi:10.1177/0272989x13505910 24246566 PMC4819801

[105] Kerr C , Robinson E , Stevens A , Braunholtz D , Edwards S , Lilford R . Randomisation in trials: do potential trial participants understand it and find it acceptable? J Med Ethics. 2004;30(1):80‐84. doi:10.1136/jme.2002.001123 14872081 PMC1757143

[106] Applebaum AJ , Lichtenthal WG , Pessin HA , et al. Factors associated with attrition from a randomized controlled trial of meaning‐centered group psychotherapy for patients with advanced cancer. Psychooncology. 2012;21(11):1195‐1204. 21751295 10.1002/pon.2013 PMC3827859

[107] Backman M , Wengström Y , Johansson B , et al. A randomized pilot study with daily walking during adjuvant chemotherapy for patients with breast and colorectal cancer. Acta Oncol. 2014;53(4):510‐520. doi:10.3109/0284186X.2013.873820 24460069

[108] Livingston PM , Craike MJ , Salmon J , et al. Effects of a clinician referral and exercise program for men who have completed active treatment for prostate cancer: a multicenter cluster randomized controlled trial (ENGAGE). Cancer. 2015;121(15):2646‐2654. doi:10.1002/cncr.29385 25877784 PMC4654333

[109] IJsbrandy C , Hermens R , Boerboom LWM , Gerritsen WR , Van Harten WH , Ottevanger PB . Implementing physical activity programs for patients with cancer in current practice: patients' experienced barriers and facilitators. J Cancer Surviv. 2019;13(5):703‐712. doi:10.1007/s11764-019-00789-3 31347009 PMC6828940

[110] Sil A , Kumar P , Kumar R , Das NK . Selection of control, randomization, blinding, and allocation concealment. Indian Dermatol Online J. 2019;10(5):601‐605. doi:10.4103/idoj.IDOJ_149_19 31544090 PMC6743387

[111] Ekelund U , Tarp J , Steene‐Johannessen J , et al. Dose–response associations between accelerometry measured physical activity and sedentary time and all cause mortality: systematic review and harmonised meta‐analysis. BMJ. 2019;366:l4570. doi:10.1136/bmj.l4570 31434697 PMC6699591

[112] Fisher A , Wardle J , Beeken RJ , Croker H , Williams K , Grimmett C . Perceived barriers and benefits to physical activity in colorectal cancer patients. Support Care Cancer. 2016;24(2):903‐910. doi:10.1007/s00520-015-2860-0 26268781 PMC4689774

[113] Turner RR , Steed L , Quirk H , et al. Interventions for promoting habitual exercise in people living with and beyond cancer. Cochrane Database Syst Rev. 2018;9:CD010192. doi:10.1002/14651858.CD010192.pub3 30229557 PMC6513653

[114] Troiano RP , Berrigan D , Dodd KW , Masse LC , Tilert T , McDowell M . Physical activity in the United States measured by accelerometer. Med Sci Sports Exerc. 2008;40(1):181‐188. doi:10.1249/mss.0b013e31815a51b3 18091006

[115] Troiano RP , McClain JJ , Brychta RJ , Chen KY . Evolution of accelerometer methods for physical activity research. Br J Sports Med. 2014;48(13):1019‐1023. doi:10.1136/bjsports-2014-093546 24782483 PMC4141534

[116] Gorzelitz J , Peppard PE , Malecki K , Gennuso K , Nieto FJ , Cadmus‐Bertram L . Predictors of discordance in self‐report versus device‐measured physical activity measurement. Ann Epidemiol. 2018;28(7):427‐431. doi:10.1016/j.annepidem.2018.03.016 29681429 PMC6500726

[117] Smith L , Lee JA , Mun J , et al. Levels and patterns of self‐reported and objectively‐measured free‐living physical activity among prostate cancer survivors: a prospective cohort study. Cancer. 2019;125(5):798‐806. doi:10.1002/cncr.31857 30516839 PMC6378115

[118] Chastin SFM , Dall PM , Tigbe WW , et al. Compliance with physical activity guidelines in a group of UK‐based postal workers using an objective monitoring technique. Eur J Appl Physiol. 2009;106(6):893‐899. doi:10.1007/s00421-009-1090-x 19488779

[119] Ryan CG , Grant PM , Tigbe WW , Granat MH . The validity and reliability of a novel activity monitor as a measure of walking. Br J Sports Med. 2006;40(9):779‐784. doi:10.1136/bjsm.2006.027276 16825270 PMC2564393

[120] Western MJ , Armstrong MEG , Islam I , Morgan K , Jones UF , Kelson MJ . The effectiveness of digital interventions for increasing physical activity in individuals of low socioeconomic status: a systematic review and meta‐analysis. Int J Behav Nutr Phys Act. 2021;18(1):148. doi:10.1186/s12966-021-01218-4 34753490 PMC8576797

[121] Greenwood E , Swanton C . Consequences of COVID‐19 for cancer care – a CRUK perspective. Nat Rev Clin Oncol. 2021;18(1):3‐4. doi:10.1038/s41571-020-00446-0 33097915 PMC7582444

[122] Schwarz N , Oyserman D . Asking questions about behavior: cognition, communication, and questionnaire construction. Am J Eval. 2001;22(2):127‐160. doi:10.1177/109821400102200202

[123] Baldeh T , MacDonald T , Kosa SD , et al. More pilot trials could plan to use qualitative data: a meta‐epidemiological study. Pilot Feasibility Stud. 2020;6(1):164. doi:10.1186/s40814-020-00712-z 33292715 PMC7597013

