# Supplementary material for: The feasibility and acceptability of an app‐based intervention with brief behavioural support (APPROACH) to promote brisk walking in people diagnosed with breast, prostate and colorectal cancer in the UK
Source: Cancer Med. 2024 Mar 26;13(6):e7124. doi: 10.1002/cam4.7124 (PMC10964176; doi:10.1002/cam4.7124)
Supplement: Supplementary file 1 — Data S1. [file CAM4-13-e7124-s001.zip › Supplementary Information Health Economic Modelling Methods_05.03.24.docx]

Promoting physical activity via a smartphone application in people living with cancer (APPROACH): Pilot Study

Supplementary Information

Health Economic Modelling Methods

Contents

[Model Structure 3](#_Toc130547200)

[Model Baseline Population 3](#_Toc130547201)

[Physical Activity Trajectories 4](#_Toc130547202)

[Physical Activity Risk Functions 6](#_Toc130547203)

[Literature Review 6](#_Toc130547204)

[Constructing continuous risk functions 8](#_Toc130547205)

[Cancer Mortality 9](#_Toc130547206)

[Other Cause Mortality 10](#_Toc130547207)

[Utilities 10](#_Toc130547208)

[Disease Costs 12](#_Toc130547209)

[Intervention 12](#_Toc130547210)

[Effectiveness 12](#_Toc130547211)

[Duration of effect 13](#_Toc130547212)

[Lag time 13](#_Toc130547213)

[Intervention cost 14](#_Toc130547214)

[Model Running and Sensitivity Analysis 14](#_Toc130547215)

[Value of Information Analysis 16](#_Toc130547216)

[References 17](#_Toc130547217)

[Appendix A: Review of Studies Linking Physical Activity & Mortality 20](#_Toc130547218)

[Appendix B: Cancer-specific Mortality Parameters 25](#_Toc130547219)

[Appendix C: Other-Cause Mortality Parameters 28](#_Toc130547220)

[Appendix D: Model Parameters and their Distributions 31](#_Toc130547221)

# Model Structure

The APPROACH model was developed as an adaptation of the health economic model used for the U@Uni study [1, 2]. This is an individual patient Markov-style model written in the R programming language, which links health behaviours directly to mortality outcomes. There are effectively only two health states in the U@Uni model: Alive and Dead, with transition from the Alive to the Dead state being dependent upon patient characteristics, health behaviours and interventions. However, in the APPROACH model, the Dead stage is split into either cancer death or other cause death as shown in Figure 1. The model has annual cycles and a lifetime horizon.

Figure 1: Diagram showing the structure of the APPROACH health economic model


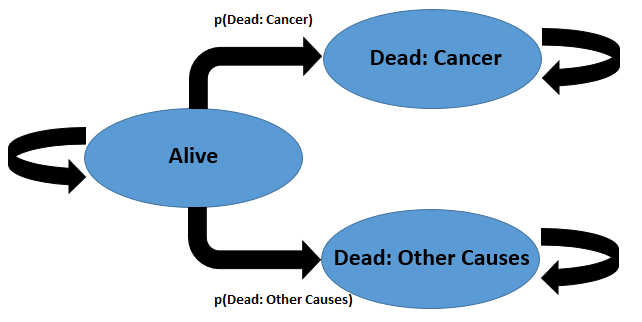


# Model Baseline Population

The model baseline population is selected at random (with replacement) from the 87 individuals who were enrolled in the APPROACH pilot (intervention and control arms combined) and who had complete baseline physical activity data. Only a small subset of the population characteristics gathered in the APPROACH pilot were used in the model to inform the baseline population. These include age, sex, cancer type (either breast cancer, prostate cancer or colorectal cancer), cancer stage (from 1 at the least severe to 4/metastatic at the most severe), baseline physical activity data, and responses to the Euroqol 5-dimensions (EQ-5D) 5 level questionnaire which enables health-related quality of life to be assessed [3]. Summary information for the baseline population from the APPROACH pilot is presented in Table 1.

Table 1: Summary information for the individuals enrolled in the APPROACH pilot (n=87 with full physical activity data)

| CHARACTERISTICS | MEAN | STANDARD DEVIATION |
| --- | --- | --- |
| Age | 62.5 | 10.4 |
| EQ-5D | 0.858 | 0.160 |
| Daily Total Stepping Minutes | 90.3 | 35.4 |
| Daily Stepping Minutes Above 100 Steps/Minute | 31.9 | 20.7 |
| Calculated METS (hours per week) | 30.39 | 12.39 |
|  | **Percentage** |  |
| Male | 51.7% |  |
| Breast Cancer | 40.2% |  |
| Prostate Cancer | 39.1% |  |
| Colorectal Cancer | 20.7% |  |
| Stage 1 Cancer | 32.2% |  |
| Stage 2 Cancer | 33.3% |  |
| Stage 3 Cancer | 26.4% |  |
| Stage 4/Metastatic Cancer | 8.0% |  |

# Physical Activity Trajectories

Whilst the U@Uni model included four different health behaviours (smoking, alcohol consumption, physical activity and fruit & vegetable consumption) [1, 2], the APPROACH model includes only physical activity. The APPROACH study collected physical activity information using two metrics – total time spent stepping and time spent stepping at a pace that is above or equal to 100 steps per minute (both in hours per day), with the primary outcome for the APPROACH study being the second of these. From this, time spent stepping at a lower rate could be calculated by subtracting the time spent stepping over 100 steps/minute from the total time spent stepping. Note that the accelerometers used in the study were able to pick up data on types of physical activity other than walking, but these would all be conceptualised in terms of steps, so more vigorous activity (e.g. running) would likely fall into the over 100 steps/minute category.

We converted these measurements into Metabolic Equivalents (known as METS), which are defined as a ratio of energy expenditure for a given task compared with energy expenditure when resting [1, 2]. There were several reasons for using METS. Firstly, the data linking physical activity to risk of mortality (see below section) primarily uses METS to define different levels of physical activity; secondly, it enables changes in both the total amount and level of physical activity to be included in the modelling, as both will have mortality benefits. Evidence suggests that a stepping rate of 100 steps per minute is roughly equivalent to 3 METS which represents the boundary between moderate and light physical activity [4], although this varies by physical activity type and personal characteristics. In the APPROACH trial, the average stepping rate ≥100 steps per minute was approximately 110 steps per minute, whilst the average rate <100 steps per minute was not collected. In the basecase analysis, it was assumed that stepping at a rate <100 steps per minute was equivalent to 2.55 METs, the midpoint between male and female confidence intervals for light activity from a 1990 paper which calculated MET values for a large range of different activities [5], whilst stepping at ≥100 steps per minute (mean 110 steps per minute) was assumed to be approximately equivalent to 3.5 METs. Note that these were parameterised as non-overlapping distributions to ensure METS ≥100 steps per minute would always be greater than METS <100 steps per minute. Total METs (in minutes per week) were calculated for each baseline individual using these values and distributions.

Physical activity varies over the life course, with the total amount declining as people age. Individual trajectories of physical activity over time in METs were estimated based on the percentile method. This assigns each individual at baseline to an activity percentile compared to the distribution in activity in a representative group of the same age. For example, someone who is very active might be on the 95^th^ percentile, meaning that 95% of individuals of their age do less physical activity and only 5% do more physical activity. Individuals are then assumed to stay on the same percentile as they age, unless intervention moves them to a different percentile. This means that their weekly METs will vary over their life course in a realistic way.

Health Survey for England (HSE) 2014 individual level data was used to obtain physical activity distributions for each age [6]. HSE was chosen as it is fairly large and representative sample of the English population. Data from three variables was selected: TotmVigWk (time spent on vigorous physical activity in the last week in minutes), TotmModWk (time spent on moderate physical activity in the last week in minutes) and TotmWalWk (time spent walking in the last week in minutes). HSE individuals not eligible for exercise data to be collected were removed from the sample (mainly those aged under 16), and missing data was recoded (assumed to be zero). Total METs (hours per week) were then calculated for each remaining person in the population, using the method described above and assuming that time spent walking corresponded to light activity as previously defined. For light, moderate and vigorous activity, MET values of 2.55 (95% CI: 1.2 - 3.9), 4.35 (95% CI: 2.8 - 5.9) and 6.15 (95% CI: 4.4 – 7.9) were chosen as the midpoint between male and female confidence intervals for light, moderate and heavy activity respectively, from a 1990 paper which calculated MET values for a large range of different activities [5].

Figure 2 shows a graph plotting change in METs over time by decile of the HSE 2014 population.

Figure 2: Calculated mean physical activity (MET hours per week) for each decile of the HSE 2014 population.


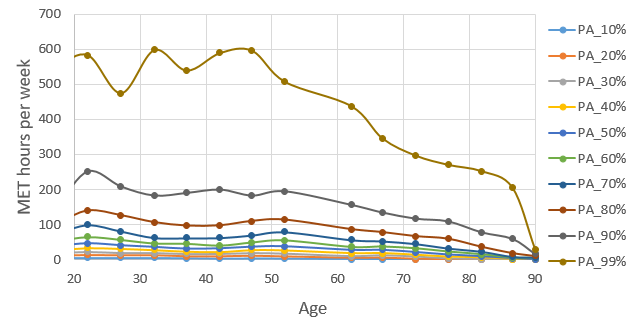


# Physical Activity Risk Functions

## Literature Review

A rapid review was carried out to identify published literature sources that linked post-diagnosis physical activity in people with breast, prostate or colorectal cancer to mortality outcomes. Searches were carried out in a single database (PubMed). Search terms included terms for cancer, physical activity (“exercise” or “physical activit*” or “sport” or “walking” or “steps”) and mortality (“mortality” or “survival” or “death” or “fatality”). In total 623 citations were identified through searches. An additional 20 citations were identified through citation searching of a systematic review produced by the 2018 Physical Activity Guidelines Advisory Committee Scientific Report [7]. After duplicates were removed this resulted in a total of 630 citations.

Inclusion and exclusion criteria were specified as given in Table 2. Using these criteria, 603 citations were excluded and the remaining 27 titles were evaluated at full text level. Three full texts were excluded because they combined survivors of different types of cancer into a mixed population for analysis and four were excluded because they didn’t include mortality outcomes. The remaining 20 studies included 11 that considered breast cancer only [8-18], five that considered prostate cancer only [19-23], two that considered colorectal cancer only [24, 25], one study that looked at breast and colorectal cancer separately [26], and one study that looked at all three cancer types separately [27] (see Appendix A for a summary). All studies indicated a significant impact of physical activity on both all-cause mortality and cancer-specific mortality (Appendix A). Most studies used weekly METS as the measure of physical activity and considered multiple MET cut-off points.

Table 2: Inclusion and exclusion criteria for a review of literature linking post-diagnosis physical activity in people with cancer to mortality outcomes.

| Selection Criteria | Inclusion | Exclusion |
| --- | --- | --- |
| Population | Adults (aged 16 years or over) with breast, prostate, or colorectal cancer at any stage | Children (aged under 16)  Adults without breast, prostate, or colorectal cancer |
| Intervention | Post-diagnosis physical activity | Pre-diagnosis physical activity |
| Comparators | No or less physical activity than intervention. | None |
| Outcomes | All-cause mortality.  Cancer specific mortality. | Outcomes for primary prevention of cancer.  Pooled mortality estimates for unspecified cancer subtypes. |
| Study type | Any type of study | None |
| Language | Studies published in English | Studies published in other languages |

Separate studies were identified for each of the three cancer types [17, 23, 26], based on a series of criteria including study type (meta-analysis preferred, followed by clinical trials and then observational studies), population (representative of all cancer stages preferred), study sample size, study location (UK or Europe preferred), study date, and reporting of outcomes (outcomes from multiple, clearly defined physical activity categories preferred). Data from the three chosen studies is shown in Table 2.

Table 3: Data used to construct continuous risk functions linking physical activity and mortality

| Continuous Risk Functions Data | | | | | | Ref |
| --- | --- | --- | --- | --- | --- | --- |
| Breast Cancer Mortality | MET hrs per week | <3 | 2.5-8.9 | ≥7.5 | ≥14.9 | [17] |
|  | Hazard Ratio | 1 | 0.68 | 0.59 | 0.62 |  |
| Breast Cancer: Other Cause Mortality | MET hrs per week | <3 | 2.5-8.9 | ≥7.5 | ≥14.9 |  |
|  | Hazard Ratio | 1 | 0.69 | 0.59 | 0.52 |  |
| Prostate Cancer Mortality | MET hrs per week | <3.5 | 3.5-8.75 | 8.75-17.5 | ≥17.5 | [23] |
|  | Hazard Ratio | 1.13 | 1 | 0.81 | 0.69 |  |
| Colorectal Cancer Mortality | MET hrs per week | <3 | +5 | +10 | +15 | [26] |
|  | Hazard Ratio | 1 | 0.86 | 0.75 | 0.65 |  |
| Colorectal Cancer: Other Cause Mortality | MET hrs per week | <3 | +5 | +10 | +15 |  |
|  | Hazard Ratio | 1 | 0.85 | 0.72 | 0.62 |  |

In general, the studies indicated that impacts on cancer-specific and all-cause mortality were fairly similar; so whilst the chosen prostate cancer study only reported impacts on cancer-specific mortality [23], it was assumed that there would be the same impact on all-cause mortality, and for all three cancer types it was assumed that the impact on other (non-cancer) causes of mortality would be similar to that for all-cause mortality. There was a further limitation with the prostate cancer study only recruiting patients with non-metastatic cancer [23]; however it was assumed that similar benefits would also accrue to patients with metastatic cancer.

## Constructing continuous risk functions

The data suggests that the relationship between physical activity and reduced mortality risk is continuous, meaning that even small changes in physical activity are likely to have some impact on mortality risk. Continuous risk functions were constructed to enable the relationship between mortality hazard ratios and physical activity to be represented. Most of the mortality hazard ratios given by the studies use the category with lowest physical activity as the reference, with higher physical activity categories having hazard ratios of below one. It was therefore necessary to adjust the hazard ratios so that a value of one corresponded to the population mean level of physical activity, enabling individuals with lower physical activity levels to have greater mortality than average, and those with higher physical activity levels to have lower mortality than average. Ideally, data from individuals with cancer would be used to adjust hazard ratios; however, the APPROACH pilot does not include sufficient individuals to do this, so HSE 2014 METs data (calculated as described above) [6], from individuals aged 40 and over (no-one enrolled in APPROACH was aged under 40), was used instead. This is a minor limitation because individuals with cancer have lower levels of physical activity than people in the general population on average, which means that more than half are likely to end up with hazard ratios over one, and this might lead to a slight overestimate of total cancer and other cause mortality in the population.

For each continuous risk function, adjusted hazard ratios for each MET category were estimated in reference to all other MET categories using the following equation:

Adj_HR^catA^ = (HR^catA^ * HSEcount ^catA^ + ... HR^catX^ * HSEcount ^catX^)/( HSEcount ^catA^ + ... HSEcount ^catX^)

Where Adj_HR = adjusted hazard ratio; HR = published hazard ratio; HSEcount = number of individuals in that MET category; catA/catX = MET categories given in published data.

For each MET category, mean METS was calculated using HSE data, and then linear regression of log mean METS against adjusted HRs was carried out to obtain a slope and intercept parameter for each continuous risk function. In general the curves fitted reasonably well with an R^2^ for each one of around 0.9-0.93. A graphical representation of the continuous risk function for prostate cancer mortality is shown in Figure 3, whilst the slope and intercept parameters for each continuous risk function are shown in Table 3.

Figure 3: A graph showing the continuous risk function for prostate cancer mortality. Average METS are plotted against adjusted hazard ratios for each physical activity category (red diamonds) and the logarithmic curve fitted to the data (black line), together with its equation and R^2^ (top right).


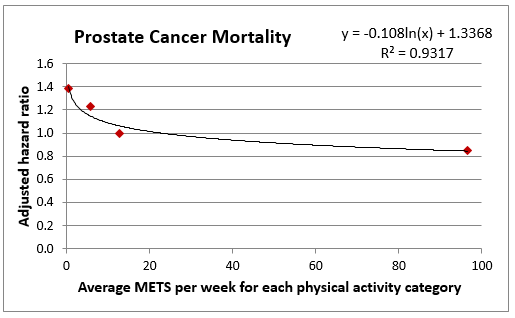


Table 4: Parameter values for continuous risk functions linking physical activity with mortality

| Continuous Risk Function | Slope | Intercept | R^2^ |
| --- | --- | --- | --- |
| Breast Cancer Mortality | -0.0958 | 1.2914 | 0.9108 |
| Breast Cancer: Other Cause Mortality | -0.1076 | 1.3198 | 0.9289 |
| Prostate Cancer Mortality | -0.1077 | 1.3368 | 0.9317 |
| Prostate Cancer: Other Cause Mortality | -0.1077 | 1.3368 | 0.9317 |
| Colorectal Cancer Mortality | -0.1087 | 1.3117 | 0.9047 |
| Colorectal Cancer: Other Cause Mortality | -0.1214 | 1.3451 | 0.8991 |

Note that the continuous risk function gives a value of infinity for anyone with 0 METS of physical activity (which is the case for many individuals particularly at older ages). To avoid this causing an error in the model all values of 0 METS were replaced with 0.1 METS. This meant that the greatest adjusted hazard ratios for people doing the least physical activity ranged between 1.51 and 1.62.

# Cancer Mortality

Cancer mortality was informed using Office for National Statistics (ONS) publicly available net survival data, based on adults diagnosed with cancer between 2013 and 2017, and followed up until 2018 [28]. Net survival data by cancer type, stage, sex and age group for one and five years after diagnosis, was extracted from the pivot table. Year one mortality was calculated directly from the year one survival data. Probability of mortality in each of years’ two to five was assumed to be constant and was calculated by converting the mortality difference between year five and year one into an annual rate and back into an annual probability. Unfortunately 10-year survival data was not available by stage and age, although there is some further cancer-specific mortality expected in this time period. The mortality rate was assumed to be half of that in years 2-5. It was assumed that there would be no further death from cancer ten years or more after diagnosis. Full cancer mortality parameters can be found in Appendix B.

Each modelled patient was assigned a risk of cancer mortality depending upon their cancer type, stage, sex and age group and time since diagnosis. It was assumed that year one mortality would correspond to the first modelled year, given that patients enrolled in APPROACH had recently finished their acute treatment. Physical activity hazard ratios obtained using the continuous risk functions described above were then applied to modify risk of cancer mortality by physical activity level for each patient.

# Other Cause Mortality

ONS life tables for the United Kingdom (2018-2020) were used to inform all-cause mortality rates by age and sex (based on the qx variable) [29]. Given that cancer mortality was estimated separately, it was necessary to subtract this from the all-cause mortality rates to get other cause mortality. This was done separately for each of the three included cancer types. To do this, publicly available ONS death certificate data for 2019 by underlying cause, age and sex was used [30]. For colorectal cancer C18 Malignant neoplasm of colon and C19 Malignant neoplasm of rectum were combined to represent colorectal cancer death. For breast cancer C50 Malignant neoplasm of breast was selected, and for prostate cancer C61 Malignant neoplasm of prostate was selected. Total deaths from all causes was also extracted. The proportion of death due to each of the three cancers by age was calculated and these values were subtracted from the all-cause mortality rate to get the other cause mortality rate. It was assumed that anyone aged beyond 100 would have 100% mortality from other causes. Each modelled patient was assigned a risk of other cause mortality for each year of their life depending upon their cancer type, sex and age. Physical activity hazard ratios obtained using the continuous risk functions described above were then applied to modify risk of other cause mortality by physical activity level for each patient. Full other cause mortality parameters can be found in Appendix C.

# Utilities

Each modelled individual has an EQ-5D score at baseline which represents their health-related quality of life. Given that patients enrolled in APPROACH are living with cancer and within 6 months of radical treatment, it is likely that many of them are still suffering symptoms or recovering from treatment. Baseline EQ-5D is therefore likely to be considerably lower than a general population of the same age. There are various studies available that assess health-related quality of life in people with cancer. These tend to indicate that quality of life is poorer in people with higher stage cancer, and often is worse in the first year after diagnosis than in subsequent years, due to the impact of treatments [31-33]. It is therefore important in the modelling to reflect any improvements in utility that are likely to occur in subsequent years compared to the first year.

For the modelling, three different sources were chosen for cancer utilities through rapid Google search and review, each one representing one of the cancer types included. For breast cancer, a 2007 Swedish study of 361 patients was chosen as this was one of the few breast cancer utility studies that provided a general estimate for first year and subsequent years, with a separate estimate given for metastatic disease [33]. For prostate cancer, utility estimates were taken from a 2014 modelling study for prostate-specific antigen screening, which combined utilities from various sources to get values to inform model health states [32]. For year one utility (all stages), an average of utility at 2-12 months after radiation therapy or prostatectomy was used; for subsequent year utility for stage 1-3, utility in the post-recovery period was taken; and for subsequent year utility for metastatic disease, utility of patients in palliative care was taken. For colorectal cancer, a 2014 review and meta-analysis of CRC utilities was used [31], which provided a linear fixed effects model for utility based on various characteristics. Utilities chosen for the APPROACH model all included the intercept and the EQ-5D coefficient, with year one utilities also including the ‘3 months after surgery’ coefficient and stage 4 utilities including the ‘stage 4’ coefficient.

It was assumed in the modelling that the baseline EQ-5D values represented the health-related quality of life in the first year after diagnosis. Utility multipliers were calculated to represent the change in health-related quality of life in subsequent years by cancer type and stage. An age decrement of 0.00432 was also applied for each subsequent year of the patients’ lives, to represent the gradual decline in quality of life due to other causes as people age [34].

Model utilities were discounted by 3.5% in line with National Institute of Health and Care Excellence (NICE) guidelines [35].

Table 5: Utilities taken from the selected studies

| Health State Informed by Utility | Mean | Lower 95% CI | Upper 95% CI | Source |
| --- | --- | --- | --- | --- |
| Breast Cancer Year 1, Stage 1-3 | 0.696 | 0.634 | 0.747 | [33] |
| Breast Cancer Year 1, Stage 4 | 0.685 | 0.62 | 0.735 |  |
| Breast Cancer Year 2+, Stage 1-3 | 0.779 | 0.7 | 0.849 |  |
| Breast Cancer Year 2+, Stage 4 | 0.685 | 0.62 | 0.735 |  |
| Prostate Cancer Year 1, Stage 1-3 | 0.775 | 0.655 | 0.895 | [32] |
| Prostate Cancer Year 1, Stage 4 | 0.775 | 0.655 | 0.895 |  |
| Prostate Cancer Year 2+, Stage 1-3 | 0.95 | 0.93 | 1 |  |
| Prostate Cancer Year 2+, Stage 4 | 0.6 | 0.24 | 0.86 |  |
| Colorectal Cancer Year 1, Stage 1-3 | 0.87 | 0.74 | 0.99 | [31] |
| Colorectal Cancer Year 1, Stage 4 | 0.68 | 0.54 | 0.81 |  |
| Colorectal Cancer Year 2+, Stage 1-3 | 0.92 | 0.8 | 1.04 |  |
| Colorectal Cancer Year 2+, Stage 4 | 0.73 | 0.61 | 0.86 |  |
| Age (additional year) | -0.00432 | -0.00460 | -0.00404 | [34] |

# Disease Costs

The model takes an NHS perspective. Some information about utilisation of NHS resources (including appointments and medications) was obtained from patients in the APPROACH pilot using the CSRI questionnaire. However, with such a small number of patients enrolled in the pilot, costs were likely to be highly variable and any differences between arms would be unlikely to be statistically significant but could be highly skewed due to chance events. We therefore chose not to use the CSRI data for this pilot analysis and instead decided to parameterise disease costs in the model using literature-based costs for cancer patients.

A single UK costing study from 2016 was identified (through previous review of cost sources during our other cancer modelling work), which covered all three cancer types [36]. This study reports NHS costs for people from up to three years prior to cancer diagnosis, to nine years after diagnosis. Costs are reported for two age groups (<65 and ≥65) and for breast and colorectal cancer are also split into early stage (1-2) and late stage (3-4). It was assumed in the modelling that individuals would incur the year one cost in the first modelled year, and subsequently incur the year 2-9 costs over the following eight years. From year 10 onwards, no disease costs were assumed to be incurred. Costs for all years were assigned in the model based on the age at diagnosis. Disease costs were inflated to 2021 costs using the NHS Cost Inflation Indices [37]. Model costs were discounted by 3.5% in line with NICE guidelines [35]. Full details of costs used in the modelling can be found in the parameters table in Appendix D.

# Intervention

## Effectiveness

Intervention effectiveness was taken from the APPROACH pilot. The pilot ran for three months, after which average total time spent stepping and time spent stepping at a rate greater or equal to 100 steps per minute was re-measured. The average difference in stepping time for each of these two measurements at 3 months compared to the baseline measurements was taken for each arm, and then the intervention effects were calculated by the trial statistician as the difference between arms (Table 5). For the >100 steps per minute variable, a separate calculation of intervention effect was performed excluding two individuals with extreme stepping values, which resulted in a much higher intervention effect. This was used in a sensitivity analysis. Note that with the larger sample of the main trial it will be possible to include individual level variation to enable intervention effectiveness to vary by cancer type, stage and other personal characteristics if there are significant differences by subgroup. However, the pilot study sample was too small to make this worthwhile for the preliminary health economic model. All individuals in the intervention arm were therefore assumed to have the mean intervention effect. As the model runs on annual cycles, the three-month intervention effect was implemented at baseline rather than with a delay.

Table 6: Effectiveness data from the APPROACH pilot study

| Parameter | Mean | 95% CI |
| --- | --- | --- |
| Change in total stepping time, intervention vs control | 0.01 | -0.2; 0.22 |
| Change in stepping time >100 steps per minute, intervention vs control | 0.09 | -0.04; 0.23 |
| Change in stepping time >100 steps per minute, intervention vs control excluding extreme values | 0.15 | 0.02; 0.27 |

## Duration of effect

No information is yet available from the APPROACH study to inform duration of intervention effect. However, a UK study of walking interventions in a general population did report data at one and three or four years of follow-up for three slightly different interventions [38]. Data for the nurse-based PACE-UP intervention, which recruited people aged 45-75, was thought to be most relevant to APPROACH. The data showed that the intervention effect almost halved at 12 months compared to 3 months; however the 12 month effect was then maintained until the end of the follow-up period at three years. Based on this, an equivalent reduction in intervention effectiveness at 12 months was implemented in our model, followed by a constant hold until a user-defined duration of effect time period had elapsed, after which no further effect was assumed to apply. This user-defined duration of effect was set to three years in the basecase analysis to reflect the follow-up time from the PACE-UP data, but was varied in sensitivity analysis to investigate the impact of the considerable uncertainty around this parameter (see below).

## Lag time

The U@Uni model included a lag time to mortality impact, which was derived from expert elicitation [1, 2]. This was required in the U@Uni model because individuals were very young at baseline, and a lack of physical activity would not affect health and mortality until they reached older ages. Evidence from the studies linking physical activity to mortality in cancer patients however, indicate that the benefits of physical activity for preventing mortality occur within a reasonably short time period (most have been measured over a small number of years), and therefore it was appropriate to assume that there would be no lag in implementation of the mortality benefits. Equally it was assumed that there would be no lag in the reduction or removal of mortality benefits once the intervention duration of effect had passed.

## Intervention cost

The intervention was costed directly from resources used in the APPROACH pilot. This included printing and posting materials which were costed directly, and nurse time for training and to deliver the intervention, which were costed using PSSRU unit costs [37]. It was assumed that a mid-Band 7 hospital nurse would deliver the intervention on an individual basis to 200 patients per year, taking 55 minutes per patient; whilst a Band 8a hospital nurse would deliver a day of training to ten Band 7 nurses, which would be valid for three years. This resulted in a total cost of £62.52 to deliver the intervention to each patient. Cost breakdowns are shown in Table 6.

Table 7: Intervention resource use and costs

| Item | Unit Cost | N Patients | Cost per Patient |
| --- | --- | --- | --- |
| Printing Materials | £127.00 | 45 | £2.82 |
| Posting Materials | £2.00 | 1 | £2.00 |
| Band 7 Nurse to deliver intervention (55 minutes) | £56.83 | 1 | £56.83 |
| Band 7 Nurse training (7.5 hours) | £465.00 | 600 | £0.78 |
| Band 8a Nurse to deliver training (7.5 hours) | £525.00 | 6000 | £0.09 |
| TOTAL | | | **£62.52** |

# Model Running and Sensitivity Analysis

Cost-effectiveness modelling was carried out using probabilistic sensitivity analysis (PSA), which produces a joint distribution of costs and QALYs for intervention and comparator arms. PSA involves running the model multiple times, each time using different parameter values randomly drawn from their probability distributions. A full table of all parameter values and their distributions for PSA can be found in Appendix C. PSA enables a true estimate of the mean output values to be obtained, taking into account model non-linearity and also enables the impact of parameter uncertainty to be assessed. For each modelling analysis, 5000 PSA runs were carried out for the cohort of 87 individuals, each with a different set of sampled parameter values.

In addition, a set of scenario analyses were carried out (each using PSA) to investigate structural uncertainties in the modelling.

1. **Intervention Effectiveness:** Different scenarios around effectiveness were implemented in sensitivity analysis. Firstly, the alternative estimate produced by excluding the two individuals with extreme values was used. Secondly, the pilot study is not sufficiently powered to detect significant impacts on physical activity, meaning that uncertainty around the mean result could be higher than that implied by the distribution implemented in the PSA. To investigate this uncertainty further, sensitivity analysis was carried out using alternative mean effectiveness values corresponding to the 95% confidence intervals for both the >100 steps per minute and the total steps per minute measures (note that in these two sensitivity analyses the mean physical activity was kept at the same value in all PSA runs, but all other parameters, including METS conversion parameters varied as normal).
2. **Intervention Costs:** An analysis was carried out to determine what level of intervention costs would change the decision based on a willingness to pay threshold of £20,000 per QALY.
3. **Duration of effect:** Sensitivity analyses were carried out to investigate uncertainty around duration of effect. Alternate values were utilised assuming either immediate return to baseline after the first year (the most pessimistic scenario), or longer return to baseline of up to ten years representing an optimistic scenario. Additional analyses were carried out to find at which point the decision changed based on a willingness to pay threshold of £20,000 per QALY.
4. **Disease costs:** Healthcare costs for people with cancer have been included in the model from external sources as there is insufficient data gathered as part of the APPROACH pilot to inform these costs. However, whilst in practice increasing physical activity is likely to reduce healthcare costs, this cannot be informed from the pilot, so the only impact the intervention has on these costs is to increase them indirectly through people living for longer as a result of doing more physical activity. This may lead to the intervention appearing less cost-effective than it actually is. Alternative scenarios were modelled where; a) disease costs were not included in the model at all; b) disease costs were assumed to be reduced by 1% in all individuals due to intervention.
5. **Population:** Sensitivity analysis was carried out around the level of baseline physical activity in the population, age, and cancer type and stage to see whether the intervention is more cost-effective in more active versus less active individuals, or in individuals of different cancer types and stages. Alternative scenarios were modelled where baseline physical activity measurements were either halved or doubled, where all patients were assumed to be stage 4 (metastatic) or early stages (stage 1 or 2), where all patients were assumed to represent only one of the cancer types (with relevant sex also selected where necessary) and where patients were aged either under 65 or over 65. This approach was taken rather than modelling each population subgroup separately, as the sensitivity analyses aimed to assess the impact of each characteristic being examined in turn, and not other characteristics that may have been correlated in the sample. For each of these analyses the intervention effect was kept at the basecase level, although it is likely that at least some of them would impact on effectiveness, even though the pilot is insufficiently powered to detect this.
6. **Discounting:** Alternative discount rates for costs and QALYs of either 1.5% or 5% were used to investigate uncertainty around discounting.

# Value of Information Analysis

Value of information analysis was carried out by applying the Sheffield Accelerated Value of Information (SAVI) tool [39] to the basecase results. This is an online tool which takes PSA parameter samples and modelled costs and QALYs corresponding to each sample as inputs, and calculates expected value of parameter information (EVPI), which enables an estimate of the expected monetary value of removing all parameter uncertainty from the decision. In addition, SAVI can perform expected value of perfect parameter information (EVPPI) analysis, providing estimates of the expected value of removing uncertainty from single or groups of parameters, and thereby enabling identification of key parameters that contribute to decision uncertainty. Note that EVPI does not estimate the value of other model uncertainties (such as structural uncertainty).

# References

1. Kruger, J., et al., *The cost-effectiveness of a theory-based online health behaviour intervention for new university students: an economic evaluation.* BMC Public Health, 2014. **14**: p. 1011.

2. Thomas, C., et al., *The cost-effectiveness of an updated theory-based online health behavior intervention for new university students: U@Uni2.* Journal of Public Health and Epidemiology, 2016. **8**(10): p. 191-203.

3. *EQ-5D*. EuroQol [cited 2022 1st November]; Available from: <https://euroqol.org/>.

4. Tudor-Locke, C., et al., *How fast is fast enough? Walking cadence (steps/min) as a practical estimate of intensity in adults: a narrative review.* Br J Sports Med, 2018. **52**(12): p. 776-788.

5. Jette, M., K. Sidney, and G. Blumchen, *Metabolic equivalents (METS) in exercise testing, exercise prescription, and evaluation of functional capacity.* Clin Cardiol, 1990. **13**(8): p. 555-65.

6. *Health Survey for England*. NHS Digital 2014 [cited 2018 1st January]; Available from: <https://digital.nhs.uk/data-and-information/publications/statistical/health-survey-for-england>.

7. *2018 Physical Activity Guidelines Advisory Committee Scientific Report*. 2018 [cited 2023 24th March]; Available from: <https://health.gov/sites/default/files/2019-09/PAG_Advisory_Committee_Report.pdf>.

8. Ammitzboll, G., et al., *Physical activity and survival in breast cancer.* Eur J Cancer, 2016. **66**: p. 67-74.

9. Beasley, J.M., et al., *Meeting the physical activity guidelines and survival after breast cancer: findings from the after breast cancer pooling project.* Breast Cancer Res Treat, 2012. **131**(2): p. 637-43.

10. Delrieu, L., et al., *Analysis of the StoRM cohort reveals physical activity to be associated with survival in metastatic breast cancer.* Sci Rep, 2020. **10**(1): p. 10757.

11. Ibrahim, E.M. and A. Al-Homaidh, *Physical activity and survival after breast cancer diagnosis: meta-analysis of published studies.* Med Oncol, 2011. **28**(3): p. 753-65.

12. Johnsson, A., et al., *Physical activity and survival following breast cancer.* Eur J Cancer Care (Engl), 2019. **28**(4): p. e13037.

13. Jones, L.W., et al., *Exercise and Prognosis on the Basis of Clinicopathologic and Molecular Features in Early-Stage Breast Cancer: The LACE and Pathways Studies.* Cancer Res, 2016. **76**(18): p. 5415-22.

14. Jung, A.Y., et al., *Pre- to postdiagnosis leisure-time physical activity and prognosis in postmenopausal breast cancer survivors.* Breast Cancer Res, 2019. **21**(1): p. 117.

15. Kehm, R.D., et al., *Recreational Physical Activity and Outcomes After Breast Cancer in Women at High Familial Risk.* JNCI Cancer Spectr, 2021. **5**(6).

16. Lahart, I.M., et al., *Physical activity, risk of death and recurrence in breast cancer survivors: A systematic review and meta-analysis of epidemiological studies.* Acta Oncol, 2015. **54**(5): p. 635-54.

17. Salam, A., et al., *Effect of post-diagnosis exercise on depression symptoms, physical functioning and mortality in breast cancer survivors: A systematic review and meta-analysis of randomized control trials.* Cancer Epidemiol, 2022. **77**: p. 102111.

18. Spei, M.E., et al., *Physical activity in breast cancer survivors: A systematic review and meta-analysis on overall and breast cancer survival.* Breast, 2019. **44**: p. 144-152.

19. Bonn, S.E., et al., *Physical activity and survival among men diagnosed with prostate cancer.* Cancer Epidemiol Biomarkers Prev, 2015. **24**(1): p. 57-64.

20. Dickerman, B.A., et al., *Guideline-Based Physical Activity and Survival Among US Men With Nonmetastatic Prostate Cancer.* Am J Epidemiol, 2019. **188**(3): p. 579-586.

21. Friedenreich, C.M., et al., *Physical Activity and Survival After Prostate Cancer.* Eur Urol, 2016. **70**(4): p. 576-585.

22. Kenfield, S.A., et al., *Physical activity and survival after prostate cancer diagnosis in the health professionals follow-up study.* J Clin Oncol, 2011. **29**(6): p. 726-32.

23. Wang, Y., et al., *Recreational Physical Activity in Relation to Prostate Cancer-specific Mortality Among Men with Nonmetastatic Prostate Cancer.* Eur Urol, 2017. **72**(6): p. 931-939.

24. Ratjen, I., et al., *Postdiagnostic physical activity, sleep duration, and TV watching and all-cause mortality among long-term colorectal cancer survivors: a prospective cohort study.* BMC Cancer, 2017. **17**(1): p. 701.

25. Wu, W., et al., *Pre- and post-diagnosis physical activity is associated with survival benefits of colorectal cancer patients: a systematic review and meta-analysis.* Oncotarget, 2016. **7**(32): p. 52095-52103.

26. Schmid, D. and M.F. Leitzmann, *Association between physical activity and mortality among breast cancer and colorectal cancer survivors: a systematic review and meta-analysis.* Annals of Oncology, 2014. **25**(7): p. 1293-1311.

27. Friedenreich, C.M., et al., *Physical Activity and Cancer Outcomes: A Precision Medicine Approach.* Clin Cancer Res, 2016. **22**(19): p. 4766-4775.

28. *Cancer Survival in England: adults diagnosed between 2013 and 2017 and followed up to 2018*. Office for National Statistics 2019 [cited 2022 22nd September]; Available from: <https://www.ons.gov.uk/peoplepopulationandcommunity/healthandsocialcare/conditionsanddiseases/datasets/cancersurvivalratescancersurvivalinenglandadultsdiagnosed>.

29. *National Life Tables 2018-2022*. Office for National Statistics 2022 [cited 2022 22nd September]; Available from: <https://www.ons.gov.uk/peoplepopulationandcommunity/birthsdeathsandmarriages/lifeexpectancies/datasets/nationallifetablesunitedkingdomreferencetables>.

30. *Mortality Statistics: Underlying Cause, Sex and Age*. Office for National Statistics 2019 [cited 2022 13th September]; Available from: <https://www.nomisweb.co.uk/>.

31. Djalalov, S., et al., *A Review and Meta-analysis of Colorectal Cancer Utilities.* Med Decis Making, 2014. **34**(6): p. 809-18.

32. Heijnsdijk, E.A., et al., *Quality-of-life effects of prostate-specific antigen screening.* N Engl J Med, 2012. **367**(7): p. 595-605.

33. Lidgren, M., et al., *Health related quality of life in different states of breast cancer.* Qual Life Res, 2007. **16**(6): p. 1073-81.

34. Ara, R. and J. Brazier, *Using health state utility values from the general population to approximate baselines in decision analytic models when condition-specific data are not available.* Value in Health, 2011. **2011**(4): p. 539-45.

35. *Guide to the methods of technology appraisal*. National Institute of Health and Care Excellence (NICE) 2013; Available from: <https://www.nice.org.uk/process/pmg9/resources/guide-to-the-methods-of-technology-appraisal-2013-pdf-2007975843781>.

36. Laudicella, M., et al., *Cost of care for cancer patients in England: evidence from population-based patient-level data.* Br J Cancer, 2016. **114**(11): p. 1286-92.

37. *Unit Costs of Health and Social Care* Personal Social Services Research Unit (PSSRU) 2021 [cited 2022 1st November]; Available from: <https://www.pssru.ac.uk/project-pages/unit-costs/unit-costs-of-health-and-social-care-2021/>.

38. Harris, T., et al., *Physical activity levels in adults and older adults 3-4 years after pedometer-based walking interventions: Long-term follow-up of participants from two randomised controlled trials in UK primary care.* PLoS Med, 2018. **15**(3): p. e1002526.

39. Strong, M., J.E. Oakley, and A. Brennan, *Estimating multiparameter partial expected value of perfect information from a probabilistic sensitivity analysis sample: a nonparametric regression approach.* Med Decis Making, 2014. **34**(3): p. 311-26.

# Appendix A: Review of Studies Linking Physical Activity & Mortality

Table 8: A summary of included citations identified in a review of studies linking post-diagnosis physical activity in people with cancer to mortality.

| **Study** | **Study Design** | **Setting** | **Population (N)** | **Type of physical activity** | **Follow-up** | **Survival Outcomes** | | | |
| --- | --- | --- | --- | --- | --- | --- | --- | --- | --- |
|  |  |  |  |  |  | **Outcome** | **Reference Group** | **Intervention Group** | **Hazard Ratio /Relative Risk**  **(95% CI)** |
| **Breast Cancer** | | | | | | | | | |
| Salam 2022 [17] | SR and meta-analysis of RCTs | Multiple | Breast cancer patients (18,214) | Various including walking and recreational activities | 1-11 years median | Cancer-specific mortality | <3 MET-h/wk | 2.5-<8.9 MET-h/wk  ≥7.5 MET-h/wk  ≥14.9 MET-h/wk | 0.68 (0.56-0.80)  0.59 (0.48-0.71)  0.62 (0.41-0.82) |
|  |  |  |  |  |  | All-cause mortality | <3 MET-h/wk | 2.5-<8.9 MET-h/wk  ≥7.5 MET-h/wk  ≥14.9 MET-h/wk | 0.69 (0.59-0.79)  0.59 (0.50-0.68)  0.53 (0.42-0.64) |
| Kehm 2021 [15] | Prospective cohort | Australia, Canada, USA, NZ | Patients having first invasive breast cancer (4,610) | Recreational physical activity | 11 years median | All-cause mortality | No recreational  physical activity (RPA) | Any RPA  Moderate RPA: 150-300 mins/wk  Strenuous RPA: 75-150 mins/wk | 0.84 (0.72-0.98)  0.82 (0.70-0.97)  0.86 (0.73-1.02) |
| Delrieu 2020 [10] | Non randomised, prospective cohort | France | Metastatic breast cancer patients within 1 year of diagnosis (833) | All activity (including walking, recreation, transport & work) | 3 years | All-cause mortality | <10 MET-h/wk | 10-50 MET-h/wk  >50 MET-h/wk | 0.95 (0.70-1.29)  0.76 (0.53-1.10) |
| Johnsson 2019 [12] | Prospective cohort | Sweden | Breast cancer patients (847) | Various including walking and recreational activities | 4-24 years | Cancer-specific mortality | PA score = 0 (no PA) | PA score = 1  PA score = 2  PA score = 3  PA score = 4  PA score = 5  PA score = 6 | 0.32 (0.05-1.95)  0.71 (0.20-2.52)  0.56 (0.16-2.04)  0.59 (0.19-1.84)  0.48 (0.15-1.55)  0.25 (0.03-2.29) |
|  |  |  |  |  |  | All-cause mortality | PA score = 0 (no PA) | PA score = 1  PA score = 2  PA score = 3  PA score = 4  PA score = 5  PA score = 6 | 0.71 (0.30-1.63)  0.47 (0.22-0.99)  0.45 (0.22-0.93)  0.43 (0.22-0.82)  0.35 (0.18-0.67)  0.29 (0.09-0.90) |
| Jung 2019 [14] | Prospective cohort | Germany | Patients with invasive breast cancer aged 50-74 (3,813) | Walking & cycling | 5.8 years median | Cancer-specific mortality | 0 MET-h/wk | >0 -<7.5 MET-h/wk  ≥7.5 MET-h/wk | 0.65 (0.37-1.16)  0.48 (0.25-0.91) |
|  |  |  |  |  |  | All-cause mortality | 0 MET-h/wk | >0 -<7.5 MET-h/wk  ≥7.5 MET-h/wk | 0.71 (0.48-1.06)  0.43 (0.26-0.72) |
| Spei 2019 [18] | SR and meta-analysis of observational studies | Multiple | Breast cancer patients (23,041) | Various including walking | 3.5-12.7 years median | Cancer-specific mortality | The lowest category in each study | The overall effect of PA | 0.87 (0.48-1.58) |
|  |  |  |  |  |  | All-cause mortality | The lowest category in each study | The overall effect of PA | 0.76 (0.48-1.20) |
| Friedenreich 2016a [27] | SR and meta-analysis of prospective cohort studies | Multiple | Breast cancer patients (17,666) | Various | 4.3-22 years median | Cancer-specific mortality | The least active participants | The most active participants | 0.62 (0.48-0.80) |
| Jones 2016 [13] | Prospective cohort | USA | Patients with early breast cancer (6,211) | Recreational physical activity | 7.2 years median | Cancer-specific mortality | <2 MET-h/wk | 2-10 MET-h/wk  >10-25 MET-h/wk  >25 MET-h/wk | 0.90 (0.68-1.19)  0.82 (0.62-1.10)  1.00 (0.74-1.34) |
| Ammitzbøll 2016 [8] | Prospective cohort | Denmark | Breast cancer patients (959) | Various including household, walking and recreational activities | 11-17 years | All-cause mortality | 0-41 MET-h/wk | >41-63 MET-h/wk  >63-97 MET-h/wk  >97 MET-h/wk  >41 MET-h/wk  Continuous (per 10 MET-h/wk) | 0.98 (0.61-1.58)  0.80 (0.48-1.31)  0.74 (0.42-1.28)  0.85 (0.57-1.29)  0.98 (0.94-1.02) |
| Lahart 2015 [16] | SR and meta-analysis of prospective cohort studies | Multiple | Breast cancer patients (123,574) | Recreational physical activity meeting guidelines | 4.3-12.7 years median | Cancer-specific mortality | <8 MET-h/wk Lowest PA | ≥8 MET-h/wk Highest PA | 0.67 (0.50-0.90)  0.59 (0.45-0.78) |
|  |  |  |  |  |  | All-cause mortality | <8 MET-h/wk Lowest PA | ≥8 MET-h/wk Highest PA | 0.54 (0.38-0.76)  0.52 (0.43-0.64) |
| Schmid 2014 [26] | SR and meta-analysis of prospective cohort studies | Multiple | Breast cancer patients (21,975) | Various | 3.3-10 years median | Cancer-specific mortality | 0 MET-h/wk  Least active | Inc of 5 MET-h/wk  Inc of 10 MET-h/wk  Inc of 15 MET-h/wk  Most active | 0.94 (0.92-0.97)  0.89 (0.85-0.94)  0.84 (0.78-0.91)  0.72 (0.60-0.85) |
|  |  |  |  |  |  | All-cause mortality | 0 MET-h/wk  Least active | Inc of 5 MET-h/wk  Inc of 10 MET-h/wk  Inc of 15 MET-h/wk  Most active | 0.87 (0.80-0.94)  0.76 (0.64-0.89)  0.66 (0.52-0.84)  0.58 (0.48-0.70) |
| Beasley 2012 [9] | Prospective cohort | UK, China, USA | Breast cancer patients (13,302) | Recreational physical activity | 2-28 years | Cancer-specific mortality | Lowest quintile (Q1)  ≤10 MET-h/wk | Q2  Q3  Q4  Q5  >10 ΜΕΤ-h/wk | 1.00 (0.83-1.21)  0.87 (0.71-1.06)  0.74 (0.60-0.91)  0.73 (0.59-0.91)  0.75 (0.65-0.85) |
|  |  |  |  |  |  | All-cause mortality | Lowest quintile (Q1)  ≤10 MET-h/wk | Q2  Q3  Q4  Q5  >10 ΜΕΤ-h/wk | 0.90 (0.77-1.04)  0.77 (0.66-0.90)  0.71 (0.60-0.84)  0.60 (0.51-0.72)  0.73 (0.66-0.82) |
| Ibrahim 2011 [11] | SR and meta-analysis | Multiple | Breast cancer patients (12,108) | Various | Not stated | Cancer-specific mortality | <3 MET-h/wk | 2.8 -<8.9 MET-h/wk  ≥8 MET-h/wk  ≥15 MET-h/wk  Overall effect of PA | 0.76 (0.61-0.95)  0.54 (0.40-0.73)  0.61 (0.46-0.81)  0.66 (0.57-0.77) |
|  |  |  |  |  |  | All-cause mortality | <3 MET-h/wk | 2.8 -<8.9 MET-h/wk  ≥8 MET-h/wk  ≥15 MET-h/wk  Overall effect of PA | 0.65 (0.55-0.76)  0.54 (0.45-0.66)  0.54 (0.45-0.66)  0.59 (0.53-0.65) |
| **Prostate Cancer** | | | | | | | | | |
| Dickerman 2019 [20] | Prospective cohort model | USA | Patients with non-metastatic prostate cancer (2,299) | Not specified | 10 years median | All-cause mortality | No PA intervention | ≥1.25 h/wk vig. PA  ≥2.5 h/wk vig. PA  ≥3.75 h/wk vig. PA  ≥2.5 h/wk mod. PA  ≥5 h/wk mod. PA  ≥7.5 h/wk mod. PA | 0.84 (0.75-0.94)  0.72 (0.58-0.88)  0.68 (0.53-0.85)  0.90 (0.84-0.94)  0.81 (0.73-0.88)  0.79 (0.71-0.86) |
| Wang 2017 [23] | Prospective cohort | USA | Patients with non-metastatic prostate cancer (5,319) | Various including walking and recreational activities | 1-20 years | Cancer-specific mortality | 3.5- <8.75 MET-h/wk | <3.5 MET-h/wk  8.75- <17.5 MET-h/wk  ≥17.5 MET-h/wk | 1.13 (0.77–1.66)  0.81 (0.58–1.15)  0.69 (0.49–0.95) |
| Friedenreich 2016a [27] | SR and meta-analysis of prospective cohort studies | Multiple | Patients with prostate cancer (8,158) | Various | 4.3-22 years median | Cancer-specific mortality | ≤42 MET-h/wk | >42-73 MET-h/wk  >73-119 MET-h/wk  >119 MET-h/wk | 0.66 (0.42-1.05)  1.02 (0.64-1.61)  0.65 (0.37-1.13) |
|  |  |  |  |  |  | All-cause mortality | ≤42 MET-h/wk | >42-73 MET-h/wk  >73-119 MET-h/wk  >119 MET-h/wk | 0.72 (0.56-0.93)  0.74 (0.57-0.97)  0.58 (0.42-0.79) |
| Friedenreich 2016b [21] | Prospective case control | Canada | Patients with stage II-IV invasive prostate cancer (988) | All activity (including walking, recreation, transport & work) | 14-17 years | Cancer-specific mortality | The least active participants | The most active participants | 0.62 (0.47-0.82) |
| Bonn 2015 [19] | Retrospective cohort | Sweden | Patients with localised prostate cancer (4,623) | Various including walking, recreational and household activities | 10-15 years | Cancer-specific mortality | <5 MET-h/d | >=5 MET-h/d | 0.78 (0.55-1.11) |
|  |  |  |  |  |  | All-cause mortality | <5 MET-h/d | >=5 MET-h/d | 0.63 (0.52-0.77) |
| Kenfield 2011 [22] | Prospective cohort | USA | Patients with non-metastatic prostate cancer (2,705) | Various including walking and recreational activities | 7.8-9.7 years median | Cancer-specific mortality | <3 MET-h/wk | 3- <9 MET-h/wk  9- <24 MET-h/wk  24- <48 MET-h/wk  >48 MET-h/wk | 0.91 (0.48-1.73)  0.6 (0.32-1.11)  0.83 (0.44-1.55)  0.42 (0.20-0.88) |
|  |  |  |  |  |  | All-cause mortality | <3 MET-h/wk | 3- <9 MET-h/wk  9- <24 MET-h/wk  24- <48 MET-h/wk  >48 MET-h/wk | 0.80 (0.61-1.06)  0.69 (0.53-0.90)  0.65 (0.49-0.86)  0.38 (0.27-0.53) |
| **Colorectal Cancer** | | | | | | | | | |
| Ratjen 2017 [24] | Prospective cohort | Germany | Patients with CRC (1,376) | Various including walking, recreational household activities | 7 years median | All-cause mortality | <64.5 MET-h/wk | >64.5-99.7 MET-h/wk  >99.7-144.9 MET-h/wk  >144.9 MET-h/wk | 0.65 (0.45-0.94)  0.52 (0.34-0.79)  0.53 (0.36-0.80) |
| Wu 2016 [25] | SR and meta-analysis of observational studies | Multiple | Patients with CRC (11,289) | Various | 3.8-11.9 years median | Cancer-specific mortality | No post-diagnosis PA | Post-diagnosis PA | 0.77 (0.63-0.94) |
|  |  |  |  |  |  | All-cause mortality | No post-diagnosis PA | Post-diagnosis PA | 0.71 (0.63-0.81) |
| Friedenreich 2016a [27] | SR and meta-analysis of prospective cohort studies | Multiple | Patients with CRC (9,698) | Various | 4.3-22 years median | Cancer-specific mortality | Least active participants | Most active participants | 0.62 (0.45-0.86) |
| Schmid 2014 [26] | SR and meta-analysis of prospective cohort studies | Multiple | Patients with CRC (6,862) | Various | 4.9-11.9 years median | Cancer-specific mortality | 0 MET-h/wk  Least active | Inc of 5 MET-h/wk  Inc of 10 MET-h/wk  Inc of 15 MET-h/wk  Most active | 0.86 (0.81-0.92)  0.75 (0.65-0.85)  0.65 (0.53-0.79)  0.61 (0.40-0.92) |
|  |  |  |  |  |  | All-cause mortality | 0 MET-h/wk  Least active | Inc of 5 MET-h/wk  Inc of 10 MET-h/wk  Inc of 15 MET-h/wk  Most active | 0.85 (0.81, 0.90)  0.72 (0.65, 0.80)  0.62 (0.53, 0.72)  0.58 (0.48-0.70) |

# Appendix B: Cancer-specific Mortality Parameters

Table 9: Breast cancer annual probabilities of mortality by age group, stage and time since diagnosis based on English breast cancer survival data from 2013-2017[28]. Note that no breast cancer survival data is available for men with breast cancer.

| Stage | Sex | Age group | Mortality Rate (years post-diagnosis) | | |
| --- | --- | --- | --- | --- | --- |
|  |  |  | **Year 1** | **Year 2-5** | **Year 6-10** |
| Stage 1 | Female | 15-44 | 0.001 | 0.006061 | 0.00303 |
|  |  | 45-54 | 0 | 0.003519 | 0.001759 |
|  |  | 55-64 | 0 | 0.002006 | 0.001003 |
|  |  | 65-74 | 0 | 0.000751 | 0.000375 |
|  |  | 75-99 | 0.004 | 0.011752 | 0.005876 |
| Stage 2 | Female | 15-44 | 0.004 | 0.023399 | 0.011699 |
|  |  | 45-54 | 0.003 | 0.014086 | 0.007043 |
|  |  | 55-64 | 0.003 | 0.015923 | 0.007961 |
|  |  | 65-74 | 0.006 | 0.017295 | 0.008647 |
|  |  | 75-99 | 0.029 | 0.042253 | 0.021127 |
| Stage 3 | Female | 15-44 | 0.026 | 0.051599 | 0.0258 |
|  |  | 45-54 | 0.016 | 0.04631 | 0.023155 |
|  |  | 55-64 | 0.02 | 0.048284 | 0.024142 |
|  |  | 65-74 | 0.032 | 0.062111 | 0.031055 |
|  |  | 75-99 | 0.093 | 0.102232 | 0.051116 |
| Stage 4 | Female | 15-44 | 0.165 | 0.152387 | 0.076193 |
|  |  | 45-54 | 0.201 | 0.141509 | 0.070755 |
|  |  | 55-64 | 0.268 | 0.179971 | 0.089985 |
|  |  | 65-74 | 0.337 | 0.213262 | 0.106631 |
|  |  | 75-99 | 0.473 | 0.255395 | 0.127697 |

Table 10: Prostate cancer annual probabilities of mortality by age group, stage and time since diagnosis based on English prostate cancer survival data from 2013-2017 [28]

| Stage | Sex | Age group | Mortality Rate (years post-diagnosis) | | |
| --- | --- | --- | --- | --- | --- |
|  |  |  | **Year 1** | **Year 2-5** | **Year 6-10** |
| Stage 1 | Male | 15-54 | 0.004 | 0.001257 | 0.000629 |
|  |  | 55-64 | 0 | 0 | 0 |
|  |  | 65-74 | 0 | 0 | 0 |
|  |  | 75-84 | 0 | 0 | 0 |
|  |  | 85-99 | 0 | 0.021696 | 0.010848 |
| Stage 2 | Male | 15-54 | 0 | 0 | 0 |
|  |  | 55-64 | 0 | 0.0005 | 0.00025 |
|  |  | 65-74 | 0 | 0 | 0 |
|  |  | 75-84 | 0 | 0 | 0 |
|  |  | 85-99 | 0 | 0.026809 | 0.013404 |
| Stage 3 | Male | 15-54 | 0 | 0.007842 | 0.003921 |
|  |  | 55-64 | 0 | 0.003519 | 0.001759 |
|  |  | 65-74 | 0 | 0.004531 | 0.002265 |
|  |  | 75-84 | 0 | 0.01067 | 0.005335 |
|  |  | 85-99 | 0.006 | 0.080031 | 0.040015 |
| Stage 4 | Male | 15-54 | 0.072 | 0.126199 | 0.0631 |
|  |  | 55-64 | 0.068 | 0.121941 | 0.060971 |
|  |  | 65-74 | 0.093 | 0.120374 | 0.060187 |
|  |  | 75-84 | 0.168 | 0.157089 | 0.078545 |
|  |  | 85-99 | 0.321 | 0.222598 | 0.111299 |

Table 11: Colorectal cancer annual probabilities of mortality by age group, sex, stage and time since diagnosis based on English colorectal cancer survival data from 2013-2017 [28]

| Stage | Sex | Age group | Mortality Rate (years post-diagnosis) | | |
| --- | --- | --- | --- | --- | --- |
|  |  |  | **Year 1** | **Year 2-5** | **Year 6-10** |
| Stage 1 | Male | 15-44 | 0.006 | 0.004813 | 0.002407 |
|  |  | 45-54 | 0.01 | 0.007405 | 0.003703 |
|  |  | 55-64 | 0.008 | 0.009458 | 0.004729 |
|  |  | 65-74 | 0.022 | 0.009332 | 0.004666 |
|  |  | 75-99 | 0.053 | 0.040397 | 0.020199 |
|  | Female | 15-44 | 0.003 | 0.00241 | 0.001205 |
|  |  | 45-54 | 0.004 | 0.003709 | 0.001855 |
|  |  | 55-64 | 0.009 | 0.008431 | 0.004215 |
|  |  | 65-74 | 0.012 | 0.0051 | 0.00255 |
|  |  | 75-99 | 0.041 | 0.030285 | 0.015142 |
| Stage 2 | Male | 15-44 | 0.019 | 0.032937 | 0.016469 |
|  |  | 45-54 | 0.013 | 0.026627 | 0.013314 |
|  |  | 55-64 | 0.029 | 0.024024 | 0.012012 |
|  |  | 65-74 | 0.049 | 0.027393 | 0.013697 |
|  |  | 75-99 | 0.115 | 0.037669 | 0.018834 |
|  | Female | 15-44 | 0.015 | 0.018261 | 0.00913 |
|  |  | 45-54 | 0.018 | 0.024567 | 0.012283 |
|  |  | 55-64 | 0.024 | 0.019519 | 0.00976 |
|  |  | 65-74 | 0.051 | 0.025171 | 0.012585 |
|  |  | 75-99 | 0.122 | 0.031654 | 0.015827 |
| Stage 3 | Male | 15-44 | 0.057 | 0.071789 | 0.035895 |
|  |  | 45-54 | 0.055 | 0.062176 | 0.031088 |
|  |  | 55-64 | 0.055 | 0.061855 | 0.030928 |
|  |  | 65-74 | 0.078 | 0.07159 | 0.035795 |
|  |  | 75-99 | 0.178 | 0.114671 | 0.057335 |
|  | Female | 15-44 | 0.033 | 0.049586 | 0.024793 |
|  |  | 45-54 | 0.044 | 0.051427 | 0.025713 |
|  |  | 55-64 | 0.045 | 0.0521 | 0.02605 |
|  |  | 65-74 | 0.09 | 0.064491 | 0.032246 |
|  |  | 75-99 | 0.241 | 0.101804 | 0.050902 |
| Stage 4 | Male | 15-44 | 0.403 | 0.251888 | 0.125944 |
|  |  | 45-54 | 0.4 | 0.290677 | 0.145339 |
|  |  | 55-64 | 0.434 | 0.294775 | 0.147387 |
|  |  | 65-74 | 0.52 | 0.281097 | 0.140548 |
|  |  | 75-99 | 0.689 | 0.373213 | 0.186606 |
|  | Female | 15-44 | 0.387 | 0.271136 | 0.135568 |
|  |  | 45-54 | 0.388 | 0.278329 | 0.139165 |
|  |  | 55-64 | 0.446 | 0.279856 | 0.139928 |
|  |  | 65-74 | 0.556 | 0.281999 | 0.141 |
|  |  | 75-99 | 0.765 | 0.324256 | 0.162128 |

# Appendix C: Other-Cause Mortality Parameters

Table 12: Annual probability of other cause mortality for people with breast, prostate or colorectal cancer calculated from UK life tables 2018-2020 [29] and death certificate data 2019 [30] (note that there are some men with breast cancer in death certificate data)

| Age | Male:  Prostate Cancer | Male:  Colorectal Cancer | Female: Colorectal Cancer | Male:  Breast Cancer | Female: Breast Cancer |
| --- | --- | --- | --- | --- | --- |
| 1 | 0.000229 | 0.000229 | 0.000214 | 0.000229 | 0.000214 |
| 2 | 0.000127 | 0.000127 | 0.000114 | 0.000127 | 0.000114 |
| 3 | 0.000102 | 0.000102 | 0.000095 | 0.000102 | 0.000095 |
| 4 | 0.000086 | 0.000086 | 0.000064 | 0.000086 | 0.000064 |
| 5 | 0.000074 | 0.000074 | 0.000074 | 0.000074 | 0.000074 |
| 6 | 0.000085 | 0.000085 | 0.000071 | 0.000085 | 0.000071 |
| 7 | 0.000067 | 0.000067 | 0.000055 | 0.000067 | 0.000055 |
| 8 | 0.000069 | 0.000069 | 0.000058 | 0.000069 | 0.000058 |
| 9 | 0.00006 | 0.00006 | 0.000051 | 0.00006 | 0.000051 |
| 10 | 0.000078 | 0.000078 | 0.000066 | 0.000078 | 0.000066 |
| 11 | 0.000077 | 0.000077 | 0.000055 | 0.000077 | 0.000055 |
| 12 | 0.000102 | 0.000102 | 0.000057 | 0.000102 | 0.000057 |
| 13 | 0.000116 | 0.000116 | 0.000087 | 0.000116 | 0.000087 |
| 14 | 0.000129 | 0.000129 | 0.000096 | 0.000129 | 0.000096 |
| 15 | 0.000172 | 0.000171 | 0.000113 | 0.000172 | 0.000113 |
| 16 | 0.000205 | 0.000204 | 0.000131 | 0.000205 | 0.000131 |
| 17 | 0.000311 | 0.00031 | 0.000158 | 0.000311 | 0.000158 |
| 18 | 0.000402 | 0.0004 | 0.000218 | 0.000402 | 0.000218 |
| 19 | 0.000454 | 0.000452 | 0.000212 | 0.000454 | 0.000212 |
| 20 | 0.000525 | 0.000525 | 0.000187 | 0.000525 | 0.000186 |
| 21 | 0.000507 | 0.000507 | 0.00021 | 0.000507 | 0.00021 |
| 22 | 0.000497 | 0.000497 | 0.000244 | 0.000497 | 0.000244 |
| 23 | 0.000524 | 0.000524 | 0.000214 | 0.000524 | 0.000214 |
| 24 | 0.000556 | 0.000556 | 0.000222 | 0.000556 | 0.000222 |
| 25 | 0.000601 | 0.000594 | 0.000258 | 0.000601 | 0.000254 |
| 26 | 0.000607 | 0.0006 | 0.000255 | 0.000607 | 0.000251 |
| 27 | 0.000629 | 0.000622 | 0.000307 | 0.000629 | 0.000303 |
| 28 | 0.000681 | 0.000673 | 0.000311 | 0.000681 | 0.000307 |
| 29 | 0.000728 | 0.00072 | 0.000335 | 0.000728 | 0.000331 |
| 30 | 0.000771 | 0.000757 | 0.000373 | 0.000771 | 0.000355 |
| 31 | 0.000835 | 0.00082 | 0.00038 | 0.000835 | 0.000362 |
| 32 | 0.000858 | 0.000842 | 0.000451 | 0.000858 | 0.00043 |
| 33 | 0.000957 | 0.000939 | 0.000475 | 0.000957 | 0.000453 |
| 34 | 0.000989 | 0.000971 | 0.000564 | 0.000989 | 0.000537 |
| 35 | 0.0011 | 0.001062 | 0.000564 | 0.0011 | 0.000526 |
| 36 | 0.001155 | 0.001115 | 0.000646 | 0.001155 | 0.000602 |
| 37 | 0.001351 | 0.001304 | 0.000725 | 0.001351 | 0.000676 |
| 38 | 0.001317 | 0.001271 | 0.000754 | 0.001317 | 0.000704 |
| 39 | 0.001457 | 0.001406 | 0.000827 | 0.001457 | 0.000771 |
| 40 | 0.001605 | 0.001557 | 0.000885 | 0.001605 | 0.000814 |
| 41 | 0.001699 | 0.001648 | 0.000962 | 0.001699 | 0.000885 |
| 42 | 0.001847 | 0.001792 | 0.001056 | 0.001847 | 0.000971 |
| 43 | 0.002014 | 0.001954 | 0.001187 | 0.002014 | 0.001092 |
| 44 | 0.002208 | 0.002143 | 0.001288 | 0.002209 | 0.001185 |
| 45 | 0.002459 | 0.002403 | 0.001438 | 0.002467 | 0.001272 |
| 46 | 0.002637 | 0.002577 | 0.001574 | 0.002645 | 0.001393 |
| 47 | 0.002734 | 0.002672 | 0.00169 | 0.002742 | 0.001495 |
| 48 | 0.00295 | 0.002883 | 0.001905 | 0.002959 | 0.001686 |
| 49 | 0.003286 | 0.003212 | 0.001987 | 0.003296 | 0.001759 |
| 50 | 0.003555 | 0.003453 | 0.002143 | 0.003577 | 0.001936 |
| 51 | 0.003797 | 0.003689 | 0.002352 | 0.003821 | 0.002125 |
| 52 | 0.00405 | 0.003934 | 0.002476 | 0.004075 | 0.002237 |
| 53 | 0.004375 | 0.00425 | 0.002651 | 0.004402 | 0.002395 |
| 54 | 0.004691 | 0.004557 | 0.002843 | 0.00472 | 0.002569 |
| 55 | 0.004977 | 0.004856 | 0.003146 | 0.005044 | 0.002932 |
| 56 | 0.005516 | 0.005382 | 0.003485 | 0.00559 | 0.003248 |
| 57 | 0.005977 | 0.005832 | 0.003764 | 0.006057 | 0.003508 |
| 58 | 0.006603 | 0.006443 | 0.004185 | 0.006692 | 0.0039 |
| 59 | 0.00714 | 0.006967 | 0.004511 | 0.007236 | 0.004204 |
| 60 | 0.007732 | 0.007579 | 0.005035 | 0.00791 | 0.004825 |
| 61 | 0.008439 | 0.008272 | 0.005409 | 0.008634 | 0.005183 |
| 62 | 0.009382 | 0.009197 | 0.006191 | 0.009598 | 0.005932 |
| 63 | 0.010312 | 0.010107 | 0.006543 | 0.010549 | 0.00627 |
| 64 | 0.010917 | 0.0107 | 0.007081 | 0.011168 | 0.006786 |
| 65 | 0.012036 | 0.011948 | 0.007807 | 0.012455 | 0.007637 |
| 66 | 0.013381 | 0.013284 | 0.008439 | 0.013848 | 0.008255 |
| 67 | 0.014279 | 0.014175 | 0.009194 | 0.014776 | 0.008993 |
| 68 | 0.015791 | 0.015676 | 0.010202 | 0.016342 | 0.00998 |
| 69 | 0.017338 | 0.017212 | 0.011026 | 0.017942 | 0.010785 |
| 70 | 0.01838 | 0.018501 | 0.012448 | 0.01923 | 0.012186 |
| 71 | 0.019868 | 0.019998 | 0.013164 | 0.020786 | 0.012887 |
| 72 | 0.021766 | 0.021909 | 0.014962 | 0.022772 | 0.014647 |
| 73 | 0.024648 | 0.024811 | 0.01669 | 0.025788 | 0.016338 |
| 74 | 0.027493 | 0.027674 | 0.019006 | 0.028764 | 0.018606 |
| 75 | 0.030757 | 0.031188 | 0.021082 | 0.032291 | 0.02091 |
| 76 | 0.034023 | 0.0345 | 0.02363 | 0.03572 | 0.023437 |
| 77 | 0.038218 | 0.038754 | 0.027041 | 0.040125 | 0.02682 |
| 78 | 0.043029 | 0.043632 | 0.030433 | 0.045175 | 0.030184 |
| 79 | 0.047858 | 0.048528 | 0.034591 | 0.050245 | 0.034308 |
| 80 | 0.053245 | 0.054366 | 0.038499 | 0.056127 | 0.038341 |
| 81 | 0.058828 | 0.060066 | 0.04386 | 0.062012 | 0.04368 |
| 82 | 0.065625 | 0.067006 | 0.048788 | 0.069177 | 0.048587 |
| 83 | 0.073486 | 0.075033 | 0.055551 | 0.077463 | 0.055322 |
| 84 | 0.082789 | 0.084533 | 0.063314 | 0.087271 | 0.063053 |
| 85 | 0.092316 | 0.094986 | 0.072035 | 0.097463 | 0.072081 |
| 86 | 0.104386 | 0.107405 | 0.082557 | 0.110206 | 0.082609 |
| 87 | 0.116307 | 0.119671 | 0.09369 | 0.122791 | 0.093749 |
| 88 | 0.130367 | 0.134138 | 0.105294 | 0.137635 | 0.105361 |
| 89 | 0.146333 | 0.150566 | 0.118784 | 0.154492 | 0.118859 |
| 90 | 0.155648 | 0.16048 | 0.134685 | 0.163468 | 0.134244 |
| 91 | 0.174533 | 0.179951 | 0.151436 | 0.183302 | 0.15094 |
| 92 | 0.191235 | 0.197172 | 0.168794 | 0.200844 | 0.168241 |
| 93 | 0.212287 | 0.218877 | 0.187095 | 0.222953 | 0.186481 |
| 94 | 0.232875 | 0.240105 | 0.205158 | 0.244575 | 0.204485 |
| 95 | 0.256052 | 0.264001 | 0.227223 | 0.268917 | 0.226478 |
| 96 | 0.276562 | 0.285148 | 0.249868 | 0.290457 | 0.249049 |
| 97 | 0.298988 | 0.308271 | 0.274312 | 0.31401 | 0.273413 |
| 98 | 0.319416 | 0.329333 | 0.295739 | 0.335464 | 0.29477 |
| 99 | 0.3522 | 0.363134 | 0.315736 | 0.369895 | 0.314701 |
| 100 | 0.371778 | 0.38332 | 0.346166 | 0.390457 | 0.345031 |

# Appendix D: Model Parameters and their Distributions

Table 13: Model parameters, their mean values, 95% confidence intervals and distributions for PSA

| Parameter | Mean | 95% CI Lower | 95% CI Upper | Distribution for PSA | Ref. |
| --- | --- | --- | --- | --- | --- |
| METS conversion vigorous PA | 6.15 | 4.4 | 7.9 | Normal | [5] |
| METS conversion moderate PA | 4.35 | 2.8 | 5.9 | Normal | [5] |
| METS conversion light PA | 2.55 | 2.1 | 3 | Uniform | [5] |
| METS conversion >100 steps per minute | 3.5 | 3 | 4 | Uniform | [4] |
| Mean change total stepping time | 0.01 | -0.2 | 0.22 | Normal | * |
| Mean change 100spm time stepping | 0.09 | -0.04 | 0.23 | Normal | * |
| Mean change 100spm time stepping EX | 0.15 | 0.02 | 0.27 | Normal | * |
| PA change 3 month duration coefficient | 1173 | 844 | 1501 | Normal | [38] |
| PA change 12 month duration coefficient | 677 | 365 | 989 | Normal | [38] |
| HR BC mortality 2.5-8.9 METS | 0.68 | 0.56 | 0.8 | Lognormal | [17] |
| HR BC mortality ≥3 METS | 0.63 | 0.55 | 0.71 | Lognormal | [17] |
| HR BC mortality ≥7.5 METS | 0.59 | 0.48 | 0.71 | Lognormal | [17] |
| HR BC mortality ≥14.9 METS | 0.62 | 0.41 | 0.82 | Lognormal | [17] |
| HR OC mortality BC patients 2.5-8.9 METS | 0.69 | 0.59 | 0.79 | Lognormal | [17] |
| HR OC mortality BC patients ≥3 METS | 0.61 | 0.55 | 0.67 | Lognormal | [17] |
| HR OC mortality BC patients ≥7.5 METS | 0.59 | 0.5 | 0.68 | Lognormal | [17] |
| HR OC mortality BC patients ≥14.9 METS | 0.53 | 0.42 | 0.64 | Lognormal | [17] |
| HR PC mortality <3.5 METS | 1.13 | 0.77 | 1.66 | Lognormal | [23] |
| HR PC mortality 8.75-17.5 METS | 0.81 | 0.58 | 1.15 | Lognormal | [23] |
| HR PC mortality ≥17.5 METS | 0.69 | 0.49 | 0.95 | Lognormal | [23] |
| HR CRC mortality +5 METS | 0.86 | 0.81 | 0.92 | Lognormal | [26] |
| HR CRC mortality +10 METS | 0.75 | 0.65 | 0.85 | Lognormal | [26] |
| HR CRC mortality +15 METS | 0.65 | 0.53 | 0.79 | Lognormal | [26] |
| HR CRC mortality high METS | 0.61 | 0.4 | 0.92 | Lognormal | [26] |
| HR OC mortality CRC patients +5 METS | 0.85 | 0.81 | 0.9 | Lognormal | [26] |
| HR OC mortality CRC patients +10 METS | 0.72 | 0.65 | 0.8 | Lognormal | [26] |
| HR OC mortality CRC patients +15 METS | 0.62 | 0.53 | 0.72 | Lognormal | [26] |
| HR OC mortality CRC patients high METS | 0.58 | 0.48 | 0.7 | Lognormal | [26] |
| Utility age decrement | 0.00432 | 0.00404 | 0.0046 | Normal | [34] |
| Utility CRC Yr1 stage 1-3 | 0.87 | 0.74 | 0.99 | Normal | [31] |
| Utility CRC Yr1 stage 4 | 0.68 | 0.54 | 0.81 | Normal | [31] |
| Utility CRC Yr2+ stage 1-3 | 0.92 | 0.8 | 1.04 | Normal | [31] |
| Utility CRC Yr2+ stage 4 | 0.73 | 0.61 | 0.86 | Normal | [31] |
| Utility PC Yr1 all stages | 0.775 | 0.655 | 0.895 | Normal | [32] |
| Utility PC Yr2+ stage 1-3 | 0.95 | 0.93 | 1 | Normal | [32] |
| Utility PC Yr2+ stage 4 | 0.6 | 0.24 | 0.86 | Normal | [32] |
| Utility BC Yr1 stage 1-3 | 0.696 | 0.634 | 0.747 | Normal | [33] |
| Utility BC Yr1 stage 4 | 0.685 | 0.62 | 0.735 | Normal | [33] |
| Utility BC Yr2+ stage 1-3 | 0.779 | 0.7 | 0.849 | Normal | [33] |
| Utility BC Yr2+ stage 4 | 0.685 | 0.62 | 0.735 | Normal | [33] |
| APPROACH intervention cost | £62.52 | £51 | £75 | Gamma | * [37] |
| Cost CRC treatment stage 1-2 Age <64 Yr1 | £18,178 | £14,791 | £21,910 | Gamma | [36] |
| Cost CRC treatment stage 1-2 Age <64 Yr2 | £4,457 | £3,626 | £5,372 | Gamma | [36] |
| Cost CRC treatment stage 1-2 Age <64 Yr3 | £3,742 | £3,044 | £4,510 | Gamma | [36] |
| Cost CRC treatment stage 1-2 Age <64 Yr4 | £2,947 | £2,397 | £3,552 | Gamma | [36] |
| Cost CRC treatment stage 1-2 Age <64 Yr5 | £2,676 | £2,177 | £3,225 | Gamma | [36] |
| Cost CRC treatment stage 1-2 Age <64 Yr6 | £1,909 | £1,553 | £2,301 | Gamma | [36] |
| Cost CRC treatment stage 1-2 Age <64 Yr7 | £1,975 | £1,607 | £2,380 | Gamma | [36] |
| Cost CRC treatment stage 1-2 Age <64 Yr8 | £1,831 | £1,490 | £2,207 | Gamma | [36] |
| Cost CRC treatment stage 1-2 Age <64 Yr9 | £1,613 | £1,312 | £1,944 | Gamma | [36] |
| Cost CRC treatment stage 1-2 Age 65+ Yr1 | £17,307 | £14,081 | £20,860 | Gamma | [36] |
| Cost CRC treatment stage 1-2 Age 65+ Yr2 | £4,412 | £3,590 | £5,318 | Gamma | [36] |
| Cost CRC treatment stage 1-2 Age 65+ Yr3 | £3,699 | £3,010 | £4,458 | Gamma | [36] |
| Cost CRC treatment stage 1-2 Age 65+ Yr4 | £3,170 | £2,579 | £3,820 | Gamma | [36] |
| Cost CRC treatment stage 1-2 Age 65+ Yr5 | £3,209 | £2,611 | £3,867 | Gamma | [36] |
| Cost CRC treatment stage 1-2 Age 65+ Yr6 | £3,237 | £2,634 | £3,901 | Gamma | [36] |
| Cost CRC treatment stage 1-2 Age 65+ Yr7 | £2,992 | £2,434 | £3,606 | Gamma | [36] |
| Cost CRC treatment stage 1-2 Age 65+ Yr8 | £3,256 | £2,649 | £3,925 | Gamma | [36] |
| Cost CRC treatment stage 1-2 Age 65+ Yr9 | £2,810 | £2,286 | £3,387 | Gamma | [36] |
| Cost CRC treatment stage 3-4 Age <64 Yr1 | £23,391 | £19,032 | £28,193 | Gamma | [36] |
| Cost CRC treatment stage 3-4 Age <64 Yr2 | £7,823 | £6,365 | £9,429 | Gamma | [36] |
| Cost CRC treatment stage 3-4 Age <64 Yr3 | £5,424 | £4,413 | £6,537 | Gamma | [36] |
| Cost CRC treatment stage 3-4 Age <64 Yr4 | £4,474 | £3,640 | £5,393 | Gamma | [36] |
| Cost CRC treatment stage 3-4 Age <64 Yr5 | £3,262 | £2,654 | £3,932 | Gamma | [36] |
| Cost CRC treatment stage 3-4 Age <64 Yr6 | £2,770 | £2,254 | £3,338 | Gamma | [36] |
| Cost CRC treatment stage 3-4 Age <64 Yr7 | £3,188 | £2,594 | £3,842 | Gamma | [36] |
| Cost CRC treatment stage 3-4 Age <64 Yr8 | £2,500 | £2,034 | £3,014 | Gamma | [36] |
| Cost CRC treatment stage 3-4 Age <64 Yr9 | £1,795 | £1,460 | £2,163 | Gamma | [36] |
| Cost CRC treatment stage 3-4 Age 65+ Yr1 | £18,788 | £15,287 | £22,645 | Gamma | [36] |
| Cost CRC treatment stage 3-4 Age 65+ Yr2 | £6,270 | £5,102 | £7,557 | Gamma | [36] |
| Cost CRC treatment stage 3-4 Age 65+ Yr3 | £4,956 | £4,032 | £5,973 | Gamma | [36] |
| Cost CRC treatment stage 3-4 Age 65+ Yr4 | £3,990 | £3,247 | £4,809 | Gamma | [36] |
| Cost CRC treatment stage 3-4 Age 65+ Yr5 | £3,766 | £3,064 | £4,539 | Gamma | [36] |
| Cost CRC treatment stage 3-4 Age 65+ Yr6 | £3,601 | £2,930 | £4,341 | Gamma | [36] |
| Cost CRC treatment stage 3-4 Age 65+ Yr7 | £2,485 | £2,022 | £2,995 | Gamma | [36] |
| Cost CRC treatment stage 3-4 Age 65+ Yr8 | £3,076 | £2,503 | £3,707 | Gamma | [36] |
| Cost CRC treatment stage 3-4 Age 65+ Yr9 | £2,504 | £2,037 | £3,018 | Gamma | [36] |
| Cost BC treatment stage 1-2 Age <64 Yr1 | £13,101 | £10,659 | £15,790 | Gamma | [36] |
| Cost BC treatment stage 1-2 Age <64 Yr2 | £4,093 | £3,330 | £4,933 | Gamma | [36] |
| Cost BC treatment stage 1-2 Age <64 Yr3 | £2,381 | £1,937 | £2,870 | Gamma | [36] |
| Cost BC treatment stage 1-2 Age <64 Yr4 | £1,984 | £1,614 | £2,391 | Gamma | [36] |
| Cost BC treatment stage 1-2 Age <64 Yr5 | £1,971 | £1,604 | £2,376 | Gamma | [36] |
| Cost BC treatment stage 1-2 Age <64 Yr6 | £1,886 | £1,535 | £2,273 | Gamma | [36] |
| Cost BC treatment stage 1-2 Age <64 Yr7 | £1,699 | £1,383 | £2,048 | Gamma | [36] |
| Cost BC treatment stage 1-2 Age <64 Yr8 | £1,678 | £1,365 | £2,022 | Gamma | [36] |
| Cost BC treatment stage 1-2 Age <64 Yr9 | £1,559 | £1,269 | £1,879 | Gamma | [36] |
| Cost BC treatment stage 1-2 Age 65+ Yr1 | £9,262 | £7,536 | £11,163 | Gamma | [36] |
| Cost BC treatment stage 1-2 Age 65+ Yr2 | £3,083 | £2,509 | £3,716 | Gamma | [36] |
| Cost BC treatment stage 1-2 Age 65+ Yr3 | £2,628 | £2,139 | £3,168 | Gamma | [36] |
| Cost BC treatment stage 1-2 Age 65+ Yr4 | £2,719 | £2,212 | £3,277 | Gamma | [36] |
| Cost BC treatment stage 1-2 Age 65+ Yr5 | £2,532 | £2,060 | £3,052 | Gamma | [36] |
| Cost BC treatment stage 1-2 Age 65+ Yr6 | £2,650 | £2,156 | £3,194 | Gamma | [36] |
| Cost BC treatment stage 1-2 Age 65+ Yr7 | £2,515 | £2,046 | £3,031 | Gamma | [36] |
| Cost BC treatment stage 1-2 Age 65+ Yr8 | £2,602 | £2,117 | £3,136 | Gamma | [36] |
| Cost BC treatment stage 1-2 Age 65+ Yr9 | £2,687 | £2,186 | £3,239 | Gamma | [36] |
| Cost BC treatment stage 3-4 Age <64 Yr1 | £16,233 | £13,208 | £19,565 | Gamma | [36] |
| Cost BC treatment stage 3-4 Age <64 Yr2 | £7,053 | £5,738 | £8,500 | Gamma | [36] |
| Cost BC treatment stage 3-4 Age <64 Yr3 | £4,611 | £3,751 | £5,557 | Gamma | [36] |
| Cost BC treatment stage 3-4 Age <64 Yr4 | £3,574 | £2,908 | £4,308 | Gamma | [36] |
| Cost BC treatment stage 3-4 Age <64 Yr5 | £3,464 | £2,818 | £4,175 | Gamma | [36] |
| Cost BC treatment stage 3-4 Age <64 Yr6 | £3,225 | £2,624 | £3,887 | Gamma | [36] |
| Cost BC treatment stage 3-4 Age <64 Yr7 | £3,192 | £2,597 | £3,847 | Gamma | [36] |
| Cost BC treatment stage 3-4 Age <64 Yr8 | £3,120 | £2,538 | £3,760 | Gamma | [36] |
| Cost BC treatment stage 3-4 Age <64 Yr9 | £2,253 | £1,833 | £2,715 | Gamma | [36] |
| Cost BC treatment stage 3-4 Age 65+ Yr1 | £10,733 | £8,733 | £12,937 | Gamma | [36] |
| Cost BC treatment stage 3-4 Age 65+ Yr2 | £4,450 | £3,621 | £5,363 | Gamma | [36] |
| Cost BC treatment stage 3-4 Age 65+ Yr3 | £3,865 | £3,144 | £4,658 | Gamma | [36] |
| Cost BC treatment stage 3-4 Age 65+ Yr4 | £3,565 | £2,900 | £4,297 | Gamma | [36] |
| Cost BC treatment stage 3-4 Age 65+ Yr5 | £3,605 | £2,933 | £4,345 | Gamma | [36] |
| Cost BC treatment stage 3-4 Age 65+ Yr6 | £3,393 | £2,761 | £4,089 | Gamma | [36] |
| Cost BC treatment stage 3-4 Age 65+ Yr7 | £3,539 | £2,880 | £4,266 | Gamma | [36] |
| Cost BC treatment stage 3-4 Age 65+ Yr8 | £2,992 | £2,434 | £3,606 | Gamma | [36] |
| Cost BC treatment stage 3-4 Age 65+ Yr9 | £3,574 | £2,908 | £4,308 | Gamma | [36] |
| Cost PC treatment Age <64 Yr1 | £6,304 | £5,129 | £7,598 | Gamma | [36] |
| Cost PC treatment Age <64 Yr2 | £2,396 | £1,949 | £2,887 | Gamma | [36] |
| Cost PC treatment Age <64 Yr3 | £2,349 | £1,911 | £2,832 | Gamma | [36] |
| Cost PC treatment Age <64 Yr4 | £1,809 | £1,472 | £2,181 | Gamma | [36] |
| Cost PC treatment Age <64 Yr5 | £1,901 | £1,546 | £2,291 | Gamma | [36] |
| Cost PC treatment Age <64 Yr6 | £1,931 | £1,571 | £2,328 | Gamma | [36] |
| Cost PC treatment Age <64 Yr7 | £1,724 | £1,403 | £2,078 | Gamma | [36] |
| Cost PC treatment Age <64 Yr8 | £1,830 | £1,489 | £2,206 | Gamma | [36] |
| Cost PC treatment Age <64 Yr9 | £1,769 | £1,439 | £2,132 | Gamma | [36] |
| Cost PC treatment Age 65+ Yr1 | £5,729 | £4,661 | £6,905 | Gamma | [36] |
| Cost PC treatment Age 65+ Yr2 | £3,298 | £2,683 | £3,975 | Gamma | [36] |
| Cost PC treatment Age 65+ Yr3 | £3,167 | £2,577 | £3,818 | Gamma | [36] |
| Cost PC treatment Age 65+ Yr4 | £3,083 | £2,509 | £3,716 | Gamma | [36] |
| Cost PC treatment Age 65+ Yr5 | £3,161 | £2,572 | £3,810 | Gamma | [36] |
| Cost PC treatment Age 65+ Yr6 | £3,092 | £2,516 | £3,726 | Gamma | [36] |
| Cost PC treatment Age 65+ Yr7 | £4,596 | £3,740 | £5,540 | Gamma | [36] |
| Cost PC treatment Age 65+ Yr8 | £3,392 | £2,760 | £4,088 | Gamma | [36] |
| Cost PC treatment Age 65+ Yr9 | £3,165 | £2,575 | £3,815 | Gamma | [36] |
| * APPROACH pilot data; CI confidence interval; PSA probabilistic sensitivity analysis; METS metabolic equivalents; PA physical activity; EX 2 extreme values excluded; HR hazard ratio; BC breast cancer; PC prostate cancer; CRC colorectal cancer; OC other cause; Yr year | | | | | |
