# Supplementary material for: The feasibility and acceptability of an app‐based intervention with brief behavioural support (APPROACH) to promote brisk walking in people diagnosed with breast, prostate and colorectal cancer in the UK
Source: Cancer Med. 2024 Mar 26;13(6):e7124. doi: 10.1002/cam4.7124 (PMC10964176; doi:10.1002/cam4.7124)
Supplement: Supplementary file 1 — Data S1. [file CAM4-13-e7124-s001.zip › Supporting information APPROACH feasibility_05.03.24_CLEAN.docx]

## Supporting information

### The Digital Behaviour Change Intervention Scale used to assess intervention engagement

**When you first used the app to track your walking how strongly do you remember experiencing (response scale 1-7, end and middle points anchored as not at all, moderately and extremely)**

Interest

Intrigue

Focus

Inattention

Distraction

Enjoyment

Annoyance

Pleasure

**When you first used the app to track your walking how long (in minutes) do you roughly think that you spent on the app that day?**

**When you first used the app to track your walking which of the app’s components do you remember visiting (tick all that apply)?**

- Setting or reviewing targets for how many daily minutes of brisk walking you’ll aim for (1, 2 or 3 Active 10s)
- Setting or reviewing your goals/reasons for why you’re doing Active 10
- Setting a daily activity reminder within the app
- Viewing your “Rewards” for achieving walking targets
- Viewing “today’s walks” (today’s feedback about the amount of walking/brisk walking you’ve done)
- Viewing “my walk’s” (reviewing the amount of walking/brisk walking you have done over the past days, weeks and months)
- Reading the help section (e.g. tips and FAQs)
- Using the links to
- Government advice
- Weight management advice
- Mental health support
- The Health Unlocked Active10 Community
- Physical Activity guidelines
- The Couch to 5K App

**We are also interested in the last (most recent) time you used the app.**

**When you last used the app how strongly do you remember experiencing (response scale 1-7, end and middle points anchored as not at all, moderately and extremely)**

Interest

Intrigue

Focus

Inattention

Distraction

Enjoyment

Annoyance

Pleasure

**When you last used the app how long (in minutes) do you roughly think that you spent on the app that day?**

**When you last used the app to track your walking which of the app’s components do you remember visiting (tick all that apply)?**

- Setting or reviewing targets for how many daily minutes of brisk walking you’ll aim for (1, 2 or 3 Active 10s)
- Setting or reviewing your goals/reasons for why you’re doing Active 10
- Setting a daily activity reminder within the app
- Viewing your “Rewards” for achieving walking targets
- Viewing “today’s walks” (today’s feedback about the amount of walking/brisk walking you’ve done)
- Viewing “my walk’s” (reviewing the amount of walking/brisk walking you have done over the past days, weeks and months)
- Reading the help section (e.g. tips and FAQs)
- Using the links to

o Government advice

o Weight management advice

o Mental health support

o The Health Unlocked Active10 Community

o Physical Activity guidelines

o The Couch to 5K App

### Reasons for declining to participate

| Supplementary Table 1. Reasons given for declining to participate in the study (N=42)^†^ | | | | |
| --- | --- | --- | --- | --- |
|  | ***N*** | ***Breast***  ***(n=15)*** | ***Colorectal***  ***(n=13)*** | ***Prostate***  ***(n=14)*** |
| No reason given | 12 | 5 | 3 | 4 |
| Felt that the study would be ‘too much’ currently | 7 (1 metastatic^‡^) | 2 | 4 | 1^‡^ |
| Too much on – treatment specifically | 6 (2 metastatic^‡^) | 4^‡^ | 0 | 2 |
| Too busy currently | 4 (1 metastatic^‡^) | 1 | 2^‡^ | 1 |
| Struggling with health/side effects | 4 (1 metastatic^‡^) | 2^‡^ | 1 | 1 |
| Too much on with treatment & had issue with giving online consent | 1 |  |  | 1 |
| Too much on currently; struggling with breathlessness & put off by website aspect | 1 |  | 1 |  |
| Too busy & doesn’t want to keep thinking about experience | 1 |  | 1 |  |
| Doesn’t want to keep thinking about experience | 1 | 1 |  |  |
| Not in good place mental health wise | 1 |  | 1 |  |
| Put off by activPAL & recent bad weather | 1 |  |  | 1 |
| Computer issues – has one but on way out | 1 |  |  | 1 |
| Doesn’t carry phone on person & doesn’t feel right person for the study | 1 |  |  | 1 |
| Wants to maintain own health & fitness activities | 1 |  |  | 1 |
| †Nine eligible individuals did not respond to the PIS (6.1%) and 4 (2.7%) were not chased further as we had met our target. | | | | |

### Completion rates table

| Supplementary Table 2. Assessment completion rates at baseline and follow up | | | |  |
| --- | --- | --- | --- | --- |
| **Assessment** | **Total n(%)** | **Intervention group n(%)** | **Control group n(%)** |  |
| Baseline questionnaire | 90/90 (100) | 44/44 (100) | 46/46 (100) |  |
| Baseline anthropometrics | 90/90 (100) | 44/44 (100) | 46/46 (100) |  |
| Baseline activPAL | 85/90 (94) | 42/44 (98) | 43/46 (98) |  |
| Follow-up questionnaire | 85/90 (94)^†^ | 41/44 (93) ^†‡^ | 44/46 (96)^§^ |  |
| Follow-up anthropometrics | 80/90 (89) | 38/44 (86)^‡^ | 42/46 (91)^§^ |  |
| Follow up activPAL | 84/90 (93) | 41/44 (93)^‡^ | 43/46 (93)^§^ |  |
| ^†^One follow-up questionnaire was only partially completed. ^‡^Two participants withdrew from the intervention group and therefore no follow up data were available. ^§^One participant in the control group was lost to follow up (deceased) and therefore no follow up data were available. One participant in the control group withdrew due to frustration with wearing the activPAL and therefore no follow up data were available. | | | | |

### Use of other apps to support physical activity

| Supplementary Table 3. Names of the apps reported by participants to support their physical activity during the study^†^ | |
| --- | --- |
| **App name** | **N** |
| **Control group** |  |
| Fitbit | 3 |
| Zwift | 1 |
| Kinomap | 1 |
| Pedometer | 1 |
| Samsung Health | 1 |
| Walk tracker^‡^ | 1 |
| Apple watch and health | 1 |
| **Intervention Group^§^** |  |
| Heroband 111 | 1 |
| Map my walk | 1 |
| Youtube | 1 |
| Google fit | 1 |
| Fitbit | 1 |
| Strava | 1 |
| Slimming world | 1 |
| Huawei Health | 1 |
| Samsung Health | 1 |
| ^†^Participants could report use of >1 app so numbers will not add up. ^‡^This participant specified ‘My walk tracker’ without further explanation but is assumed that it may part of their phone health app, e.g., Apple Health, Samsung Health. ^§^Excluding reporting of Active 10 use. | |

### Detailed results of the Digital Behaviour Change Intervention Scale

| Supplementary Table 4. Intervention participant use of the different key components of the Active 10 app | | | | | |
| --- | --- | --- | --- | --- | --- |
|  | | **Number of participants who ticked using each component at first use and last use of the app (%)^†^** | | | |
| **App component** | | **First use** | | **Last use** | |
| Setting or reviewing targets | | 35 (85.4) | | 18 (43.9) | |
| Setting or reviewing goals for why doing Active 10 | | 21 (51.2) | | 9 (22.0) | |
| Setting reminder within app | | 12 (29.3) | | 4 (9.8) | |
| Viewing rewards | | 31 (75.6) | | 19 (46.3) | |
| Viewing Today’s walks | | 34 (82.9) | | 34 (82.9) | |
| Viewing My walks | | 33 (80.5) | | 30 (73.2) | |
| ^†^% calculated out of the 41 participants who completed the intervention feedback section of the T1 questionnaire. Two participants withdrew several weeks after randomisation, and one did not complete this intervention feedback section of the questionnaire. Two participants reported not downloading the app and weren’t shown any further questions on app use. | | | | | |

### Trial experience interviews

| Supplementary Table 5. Illustrative quotes and frequency counts about acceptability of randomisation^†^ | | |  |
| --- | --- | --- | --- |
| **Feedback** | **n** | **Supporting quotes *(sex, age [in years], experimental group)*** | |
| Gratitude in the intervention group | 27 | “I guess I felt pleased to be part of it and felt it would be beneficial to me and I think it was” *(Female, 60, intervention)*  “I was glad because I don’t know that I would have done it myself” *(Female, 40, intervention)* |  |
| Indifference about randomisation | 23 | “I’m just one cog in this whole thing and it didn’t matter to me which way or the other way I was going” *(Female, 47, control)*  “I was okay with it, as I say I had no issues with it at all” *(Male, 73, intervention)* |  |
| Disappointment but understanding in the role of the control group | 14 | “Well I think disappointed, yes, because I was looking forward to seeing what was going to be on the app and how it was going to work” *(Female, 61, control)*  “I was disappointed to start with…But that was like five seconds…And then I understood. It’s like, you know, both are required for the study” *(Male, 64, control)* | |
| Not understanding or knowing about randomisation | 6 | “I didn’t really understand between the groups was, so I just stayed with you know what I call the standard army issue one…Well was the other one going into meetings or sitting down with people in a circle and all saying “I’ve got cancer” and blah de blah de blah” *(Male, 69, intervention)*  “Well I didn’t know what group I was in. I haven’t been told what group I’m in. I still don’t know what group I’m in.” *(Female, 66, control)* |  |
| ^†^Two participants were not asked about the acceptability of randomisation. | | |  |

| Supplementary Table 6. Illustrative quotes and frequency counts about the acceptability of study assessments | | |
| --- | --- | --- |
| **Feedback** | **n** | **Supporting quotes *(sex, age [in years], experimental group)*** |
| **Wearing the ActivPAL^†^** |  |  |
| Satisfaction with wearing the activPAL | 53 | “Once you’ve got it on you do tend to forget about it” *(Female, 61, control)*  “Didn’t bother me at all, not one bit. Just put it in and forgot about it really” *(Female, 62, intervention)*  “Fine, yes. Absolutely fine, yes, no problems at all” *(Female, 47, control)* |
| Discomfort wearing the activPAL | 14 | “A little bit of discomfort on a certain time because it kept pulling hairs on my legs” *(Male, 60, intervention)*  “It was itching me sometimes, so that was bothering me a little bit” *(Female, 56, intervention)*  “a little bit of red and soreness” *(Male, 41, control)* |
| ActivPAL fell off during the wear-period | 4 | “so on a couple of occasions it fell off” *(Male, 41, control)*  “the little guy bailed out three and a half days into the seven day period” *(Male, 73, control)* |
| More instructions about the activPAL are needed | 3 | “It was the peeling back those bits – don’t understand how that fitted with what I was doing” “Seeing it done in action to replicate it, then it’s easier to do**”** *(Female, 48, control)*  “I wouldn’t have known how to put it on you see if I didn’t have this other sticky tape” *(Female, 67, intervention)*  “I wondered whether I was a bit confused by what comes away and what doesn’t” *(Male, 76, intervention)* |
| **Completing body measurements^‡^** |  |  |
| Satisfaction with completion of body measurements | 58 | “Yes, yes, it was no problem doing them at home” *(Female, 59, control)*  “That was fine, yeah, didn’t bother me at all” *(Male, 69, intervention)* |
| Issues with completion of body measurements | 3 | “You know, I’m looking at it and thinking, and then you tighten it up a bit and then you slacken off a bit and you wonder what is the exact measurement” *(Male, 76 intervention)*  “It wouldn’t take the numbers and that for the weight” *(Male, 58, intervention)*  “Yes I didn’t understand why I was doing that” *(Male, 57, control)* |
| **Completion of study questionnaires^§^** |  |  |
| Satisfaction with the length of the questionnaires | 55 | “So, yes I didn’t find it a problem at all anyway doing that” *(Female, 58, control)*  “I did have to find like twenty minutes to be able to do it but no that was fine” *(Female, 41, intervention)*  “That was okay, I had no problem, 10 minutes is not a big issue” *(Male, 73, intervention)* |
| Dissatisfaction with the length/relevance of the questionnaires | 15 | “The answers you were looking for I think you could’ve achieved in less questions” *(Female, 62, control)*  “Yes it was quite draining that, wasn’t it” *(Male, 74, intervention)*  “Yeah a lot of them were like over and over, sort of saying the same question in a different way, but it just wasn’t relevant to me” *(Female, 62, intervention)* |
| Emotions induced by the questionnaires | 1 | “We’ve just got to get on with it and I felt like it started making me think about it all again” *(Female, 67, intervention)* |
| ^†^Two participants did not wear the activPAL. ^‡^11 participants were not asked about completing the body measurements. ^§^One participant reported not remembering the questionnaire completion. | | |

| Supplementary Table 7. Illustrative quotes and frequency counts about the timing of being approached to take part in the study^†^ | | |
| --- | --- | --- |
| **Feedback** | **N** | **Supporting quotes *(sex, age [in years], experimental group)*** |
| Satisfied with timing of being approached | 60 | “Yes it was fine for me. If I could have helped earlier, I would have” *(Female, 66, control)*  “I don’t think it matters, it’s down to a person, how a person reacts to it” *(Male, 69, intervention)*  “For me it was good because it was a distraction. It was something to do, something I had control over” *(Female, 62, control)* |
| Being approached after treatment is preferable | 6 | “Well it was a bit awkward because I was still undergoing radiotherapy, so initially I couldn’t do much at all because I was sort of all day at the hospital *(Male, 74 intervention)*  “Yes I would probably say maybe a few weeks after, two or three weeks afterwards to try and build your fitness levels up and that again” *(Male, 59, intervention)* |
| Mixed feelings about the timing of being approached | 3 | “Yes and no…I thought if I had given a choice of which week to wear or something it might have been better because then next week I could’ve worn it whole week so that is another thing” *(Female, 49, control)*  “Well yes and no…I wanted to take part but it was, because of the hormone treatment…I couldn’t process or play golf regular, like go for walks or anything like that” *(Male, 75, control)*  “Yes and no, I think I was still a bit depressed…at the same time I thought, well if it’s going to help me get going and as I say get out of this hole, start doing things I thought it was fine, a bit of both” *(Female, 67, intervention)* |
| ^†^Three participants were not asked about how they felt about the timing of being approached. | | |

| Supplementary Table 8. Illustrative quotes and frequency counts about participant willingness to link data with Hospital Episode Statistics and the National Cancer Registration and Analysis Service^†^ | | |
| --- | --- | --- |
| **Feedback** | **n** | **Supporting quotes *(sex, age [in years], experimental group)*** |
| Willingness to consent to linking their data | 71 | “I’d rather give the information, if it helps somebody else and helps you along the way long term no, fine do it” *(Female, 58, control)*  “yeah, and that don’t worry me at all. I’m hoping it might help people in the future so” *(Male, 69, intervention)*  “No I can’t remember but it would certainly have been okay, I would have agreed to that” *(Male, 68, control)* |
| ^†^One participant did not remember giving consent for this and did not explain further. | | |

### Cost effectiveness model

| Supplementary Table 9. Summary of incremental per person scenario analysis results^†^ | | | | | |
| --- | --- | --- | --- | --- | --- |
| **Scenario** | **Costs** | **QALYs** | **INMB** | **ICER** | **Prob. CE** |
| Basecase | £69.02 | 0.0019 | -£31.17 | £36,475 | 0.37 |
| No extreme values PA effectiveness | £72.18 | 0.0028 | -£15.35 | £25,403 | 0.45 |
| Upper bound PA effectiveness | £102.01 | 0.0120 | £138.05 | £8,499 | 1.00 |
| Lower bound PA effectiveness | £28.95 | -0.0098 | -£225.45 | -£2,947 | 0.00 |
| No treatment costs | £62.74 | 0.0019 | -£24.89 | £33,156 | 0.42 |
| Treatment costs 99% in intervention | -£72.62 | 0.0019 | £110.47 | -£38,380 | 0.92 |
| 1 year duration of effect | £64.49 | 0.0004 | -£56.58 | £163,026 | 0.00 |
| 5 year duration of effect | £70.81 | 0.0029 | -£13.72 | £24,805 | 0.46 |
| 7 year duration of effect | £71.54 | 0.0034 | -£3.56 | £21,048 | 0.49 |
| 10 year duration of effect | £71.89 | 0.0041 | £11.03 | £17,339 | 0.53 |
| Discount rate 1.5% | £69.38 | 0.0023 | -£23.95 | £30,541 | 0.42 |
| Discount rate 5% | £68.77 | 0.0017 | -£35.20 | £40,974 | 0.32 |
| Population baseline PA halved | £71.53 | 0.0027 | -£16.87 | £26,175 | 0.47 |
| Population baseline PA doubled | £65.56 | 0.0009 | -£48.08 | £74,994 | 0.11 |
| Population age <65 | £66.60 | 0.0014 | -£38.50 | £47,397 | 0.30 |
| Population age ≥65 | £73.85 | 0.0027 | -£19.95 | £27,400 | 0.44 |
| Population all colorectal cancer | £76.94 | 0.0031 | -£14.64 | £24,699 | 0.46 |
| Population all breast cancer (female) | £69.26 | 0.0021 | -£27.01 | £32,790 | 0.40 |
| Population all prostate cancer (male) | £64.50 | 0.0008 | -£49.41 | £85,457 | 0.12 |
| Population all cancer stage 4 | £87.66 | 0.0047 | £7.02 | £18,518 | 0.52 |
| Population all cancer stage 1 or 2 | £65.53 | 0.0011 | -£42.96 | £58,063 | 0.19 |
| Abbreviations: QALY=quality-adjusted life year; INMB=incremental net monetary benefit; ICER=incremental cost-effectiveness ratio; prob.CE=probability cost effective; PA=physical activity.  ^†^Net monetary benefit and probability cost-effective represent the £20,000 per QALY threshold. | | | | | |
